# The dysadherin/carbonic anhydrase 9 axis shapes an acidic tumor microenvironment to promote colorectal cancer progression

**DOI:** 10.1038/s41392-025-02543-x

**Published:** 2026-01-15

**Authors:** Choong-Jae Lee, Hyeon-Ji Yun, Tae-Young Jang, So-El Jeon, Yeong-Hoon Cho, Da-Ye Lim, Eun-Ju Han, Sun-Young Kong, Jeong-Seok Nam

**Affiliations:** 1 Department of Life Sciences, Gwangju Institute of Science and Technology, Gwangju, Republic of Korea; 2 Targeted Therapy Branch, Research Institute, National Cancer Center, Goyang, Republic of Korea; 3 Cancer Biomedical Science, National Cancer Center, Goyang, Republic of Korea; 4 Department of Laboratory Medicine, National Cancer Center, Goyang, Republic of Korea

**Keywords:** Cancer microenvironment, Gastrointestinal cancer

## Abstract

The tumor microenvironment (TME) plays a central role in cancer progression and metastasis. A key feature of the TME is extracellular acidity, which promotes disease progression, immune evasion, and drug resistance. Tumor acidity is increasingly recognized as a critical factor in cancer development and a negative prognostic indicator. Here, we demonstrate that the membrane glycoprotein dysadherin promotes colorectal cancer (CRC) malignancy by modulating TME acidity. Comprehensive bioinformatics and pathological analyses of CRC patient samples revealed that increased tumor acidity is a hallmark of CRC progression and strongly correlates with high expression of dysadherin. Functional studies confirmed that dysadherin enhances malignant traits, particularly under acidic conditions. Mechanistically, dysadherin activates the integrin/FAK/STAT3 signaling pathway, leading to the upregulation of carbonic anhydrase 9 (CA9). CA9 facilitates proton export, contributing to extracellular acidification while maintaining intracellular pH homeostasis, thereby enabling cancer cells to survive and thrive in acidic environments. In a murine liver metastasis model, dysadherin deletion impaired cellular adaptation to the acidic TME and markedly attenuated metastatic colonization, whereas restoring CA9 expression effectively rescued metastatic potential. Overall, our findings identify the dysadherin/CA9 axis as a potential therapeutic target in CRC and provide new insights into how tumors exploit acidosis to drive malignant development and progression.

## Introduction

Colorectal cancer (CRC) is among the most common and deadliest cancers worldwide. As it is often diagnosed at advanced stages, this disease has limited treatment options and a poor prognosis. While CRC development is influenced primarily by genetic mutations in oncogenes or tumor suppressor genes, the tumor microenvironment (TME) also plays a critical role in cancer progression.^1^ During tumor progression, the TME undergoes substantial alterations in cellular components, including the activation of immune cells and fibroblasts, as well as noncellular changes such as hypoxia and nutrient deprivation.^2–4^ These harsh conditions drive gene expression changes, epithelial–mesenchymal transition (EMT), angiogenesis, and alterations in tumor metabolism.^5–9^ Collectively, these TME-driven changes contribute to tumor growth, dissemination, and treatment resistance, making the TME an important area of study for understanding CRC malignancy.

Solid tumors face hostile conditions such as hypoxia and nutrient scarcity due to rapid growth and high metabolic demands.^10,11^ To adapt and survive, tumor cells undergo metabolic reprogramming, shifting their energy production toward glycolysis, which generates lactic acid and creates an acidic TME, a hallmark of solid tumors.^12–15^ Acidity can trigger the proapoptotic signaling pathway and disrupt protein function by altering amino acid ionization, ultimately leading to cell death. However, tumor cells develop adaptive mechanisms to neutralize the intracellular pH via proton transporters, allowing them to survive and thrive in an acidic TME.^16,17^ Tumor acidity is associated with disease progression, immune evasion, and therapeutic resistance through multiple interconnected mechanisms.^18–22^ It stabilizes and activates HIF-1α, leading to the upregulation of angiogenic factors, and enhancing glycolytic metabolism, thereby maintaining a hostile and immunosuppressive TME. In addition, the acidic microenvironment activates proteolytic enzymes, promoting extracellular matrix (ECM) remodeling and local invasion. Clinical evidence further supports tumor acidity as a negative prognostic marker, as it is correlated with poor outcomes in several cancer types.^21,22^ Accordingly, pH-targeted therapies are under investigation, and a deeper understanding of tumor acidity may inform more effective treatment strategies.

Dysadherin, a cell membrane glycoprotein with an FXYD motif, promotes aggressive tumor characteristics and is often overexpressed in various cancers, including colorectal, breast, liver, and pancreatic tumors, but is rarely expressed in normal tissues.^23–27^ Clinical studies have linked high dysadherin expression with recurrence and a poor prognosis.^24,27,28^ A recent study confirmed that genetic depletion of dysadherin attenuated intestinal tumorigenesis and progression in both *Apc*^Min/+^ mice and azoxymethane (AOM)/dextran sodium sulfate (DSS)-treated mice.^27^ In addition, dysadherin expression is heterogeneous within tumors, and highly dysadherin-expressing cells exhibit malignant phenotypes, as shown by single-cell RNA sequencing (scRNA-seq) analysis.^29^ Functional studies involving dysadherin overexpression (OE) and gene silencing in cancer cells confirmed that dysadherin promotes cancer cell growth, survival, migration, invasion, and self-renewal.^25–27,29,30^ A recent study further demonstrated that dysadherin plays a crucial role in tumor-TME interactions, contributing to tumor malignancy.^31^ Additionally, our previous research demonstrated that dysadherin promotes cancer cell adhesion to fibronectin, thereby triggering integrin/focal adhesion kinase (FAK) signaling.^27^ Notably, integrin/FAK signaling has been shown to support cancer cell survival under adverse conditions, such as TME-induced stress during tumor progression.^32^ These findings underscore the role of dysadherin in tumor cell adaptation to hostile microenvironments and highlight the need for further mechanistic investigations.

In this study, we elucidated the role of dysadherin in facilitating CRC cell adaptation to an acidic TME and sustaining malignant phenotypes through carbonic anhydrase 9 (CA9) regulation via both clinical and functional analyses. Notably, activation of the dysadherin/CA9 axis neutralizes the intracellular pH, contributing to a decrease in acidity-induced cell death and an increase in cell proliferation and survival in CRC cells and xenograft mouse models. These findings provide key mechanistic insights into the role of dysadherin in tumor adaptation, potentially paving the way for new targeted therapeutic strategies for CRC.

## Results

### Tumor acidosis is positively linked to dysadherin expression and contributes to CRC progression

**Fig. 1:**
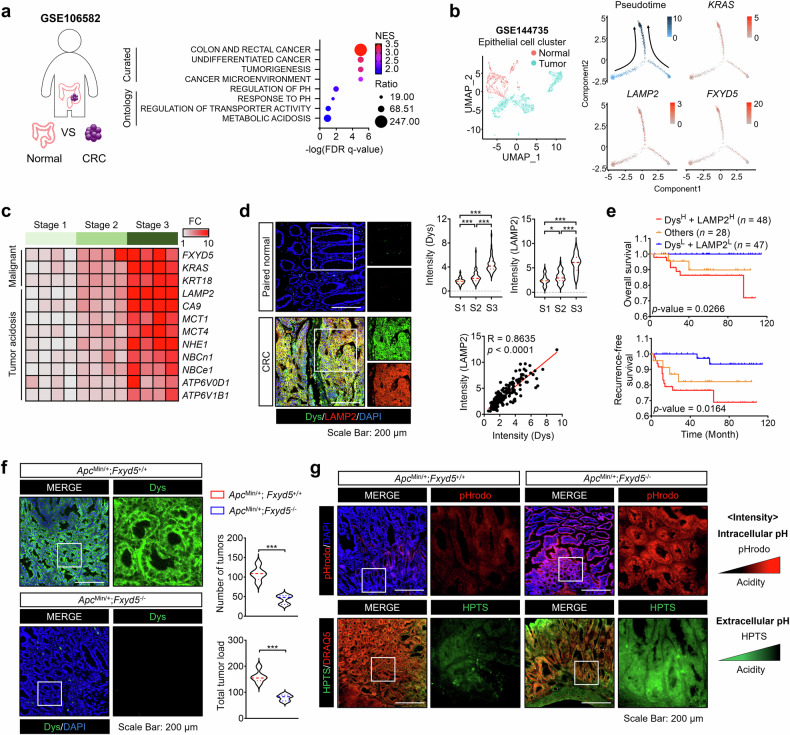
An acidic TME is positively associated with dysadherin expression and contributes to CRC progression. **a** A list of DEGs in tumor tissue compared with normal tissue was obtained from the GEO database (GSE106582) (*p* < 0.001). The gene list was analyzed via a curated gene set and the GO gene set of GSEA to determine the associated gene signatures. **b** A UMAP plot of the epithelial cell cluster from CRC patients (GSE144735) was generated. Trajectory analysis was performed via Monocle2, which orders cells on the basis of their malignant progression states. The expression patterns of malignancy-associated and tumor-acidosis–related genes were examined along the inferred trajectory. **c** Heatmap comparing the relative expression levels of malignancy and tumor-acidosis-related genes in CRC patient tissues (*n* = 4/group). **d** IF analysis of dysadherin and LAMP2 expression in CRC patient tissues, with samples grouped according to tumor stage. (S1, *n* = 45; S2, *n* = 62; S3, *n* = 61; total, *n* = 168). Graphs showing the intensity and correlation of dysadherin and LAMP2 protein expression. **e** Kaplan–Meier survival analysis was performed on patients with stage II and III CRC. Patients were divided into three groups on the basis of dysadherin and LAMP2 expression: high (*n* = 52), low (*n* = 45), and others (*n* = 26), for a total of 123 patients. Statistical significance was determined by log-rank tests. **f** IF analysis of dysadherin in the intestines of 24-week-old *Apc*^Min/+^ mice with or without dysadherin (*Fxyd5*) KO (*n* = 5/group). The upper violin plot shows the number of visible tumors per mouse. The lower violin plot shows the tumor load, which is calculated as a weighted sum that incorporates both tumor number and size: (small tumors × 1) + (medium tumors × 2) + (large tumors × 3). **g** pHrodo and HPTS staining of mouse intestinal tumors (*n* = 5/group). The data are shown as the means ± SEMs. *, **, and *** denote *p* < 0.05, *p* < 0.01, and *p* < 0.001, respectively. Statistical significance was assessed via unpaired two-tailed Student’s t-tests for two-group comparisons and one-way ANOVA with Dunnett’s multiple comparison test for analyses involving three groups. Dys dysadherin, S1 stage 1, S2 stage 2, S3 stage 3

Extracellular acidosis is a hallmark of the TME in solid cancers and promotes cancer survival, metastasis, and therapy resistance.^20^ To investigate its role in CRC, we analyzed gene expression profiles from normal and tumor tissues via public datasets (GSE106582 and GSE18105). Gene set enrichment analysis (GSEA) revealed significant enrichment of acidosis-related pathways, including “cellular response to acidic pH” and “metabolic acidosis”. Several key genes associated with extracellular acidification, such as *SLC4A4, SLC9A2*, and *SLC9A1*, were also consistently upregulated in tumors (Fig. 1a, Supplementary Fig. 1a, Table S1). Furthermore, we analyzed the scRNA-seq dataset (GSE144735) and stratified tumor cells into *dysadherin* (*FXYD5*) high and low expression groups on the basis of median gene expression levels. Differentially expressed gene (DEG) analysis followed by pathway enrichment analysis revealed significant enrichment of acidosis-related signatures in the dysadherin high expression group (Supplementary Fig. 1b). To further explore the relationship between acidosis and CRC progression, we conducted pseudotime trajectory analysis using tumor clusters from the GSE144735 dataset. As CRC cells progressed, as indicated by *KRAS* expression, the expression of tumor acidosis markers (*LAMP2, GLUT1*, and *LDHA)*, as well as *dysadherin*, also increased (Fig. 1b, Supplementary Fig. 1c). *Dysadherin* expression was strongly positively correlated with individual acidosis-related genes and a tumor acidosis signature across multiple datasets, including the GSE106582, GSE18105, GSE144735 and TCGA datasets (Supplementary Fig. 1d). Network analysis supported this link by connecting tumor acidosis signatures to dysadherin expression (Supplementary Fig. 1e). Moreover, both dysadherin and tumor acidosis-related genes were more highly expressed in patients with advanced disease stages (Fig. 1c). Consistently, analysis of CRC patient tissues (Table S2) revealed a stage-dependent increase in dysadherin and LAMP2 expression, along with a positive correlation between the two markers (Fig. 1d, Supplementary Fig. 1f, g). Notably, patients with high dysadherin or LAMP2 expression had short overall survival (OS) and recurrence-free survival (RFS) (Supplementary Fig. 1h). Combined analysis revealed that co-high expression of both markers was associated with significantly worse OS and RFS outcomes than low-expression was (Fig. 1e). These findings were corroborated by an independent open-source dataset (GSE106584) (Supplementary Fig. 1i). Collectively, these results indicate that an acidic TME is a key feature of CRC progression and is correlated with dysadherin expression.

To experimentally validate the relationship between tumor acidity and dysadherin in intestinal tumorigenesis, we utilized the dysadherin knockout (KO) *Apc*^Min/+^ mouse model (*Apc*^Min/+^; *Fxyd5*^−/^^−^) developed in a previous study.^27^ Dysadherin expression was primarily restricted to tumor epithelial cells, which is consistent with previous reports demonstrating its limited expression in nonmalignant epithelial tissues and other cell types^23,25,33^ (Supplementary Fig. 2a). Compared with those in control mice (*Apc*^Min/+^; *Fxyd5*^+/+^), the number of tumors and total tumor load in dysadherin KO (*Fxyd5*^*−/−*^) mice were significantly lower (Fig. 1f, Supplementary Fig. 2b, c). To assess cellular acidity during tumor progression, we used pHrodo staining to measure intracellular acidity and 8-Hydroxypyrene-1,3,6-trisulfonic acid (HPTS) staining for extracellular acidity. In situ calibration confirmed that the HPTS fluorescence intensity increased with higher extracellular pH (more alkaline conditions) under our imaging settings (Supplementary Fig. 2d). In dysadherin-deficient tumors and organoids, pHrodo fluorescence revealed increased intracellular acidity, whereas stronger HPTS fluorescence (450 nm excitation) reflected a more alkaline extracellular pH (Fig. 1g, Supplementary Fig. 2e, f). Notably, similar pH trends were observed in size-matched intestinal tumors and organoids, thereby minimizing the influence of tumor size–related hypoxia (Supplementary Fig. 2g, h). These findings suggest that dysadherin alters the acidity of the TME and drives CRC progression.

### Dysadherin promotes CRC tumorigenicity under acidic TME conditions

**Fig. 2:**
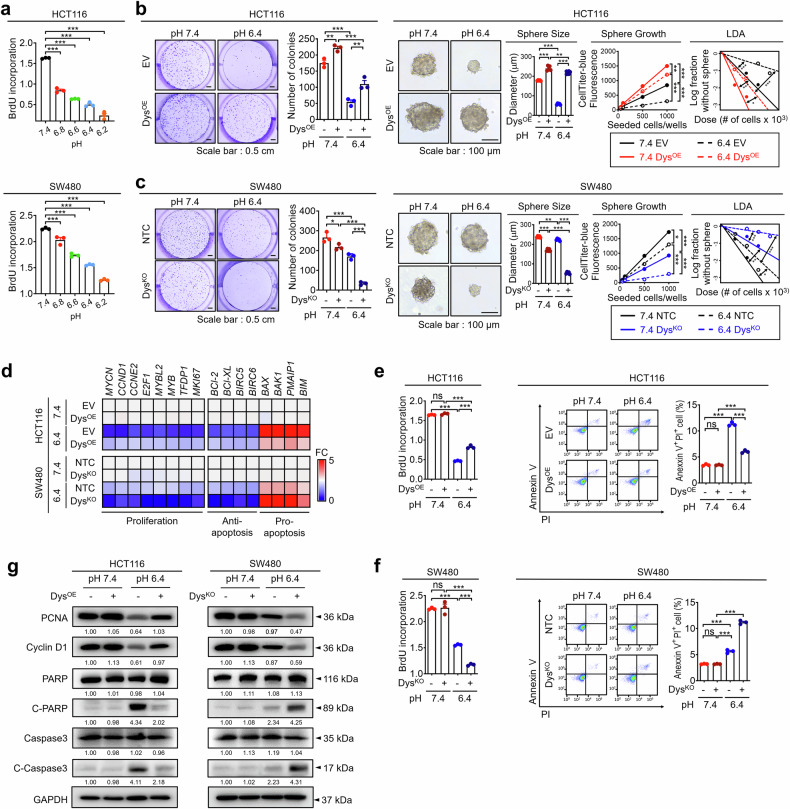
Dysadherin regulates the malignant phenotypes of CRC cells in the acidic TME. **a** Proliferation of CRC cells at various pH values was measured by a BrdU assay (*n* = 3/group). The survival potential and self-renewal properties of dysadherin-OE HCT116 cells (**b**) and dysadherin KO SW480 cells (**c**) were measured at pH 7.4 or 6.4 (*n* = 3/group). **d** Heatmap comparing the relative expression of genes related to proliferation and apoptosis in dysadherin OE HCT116 cells and dysadherin KO SW480 cells after incubation at pH 7.4 or 6.4 (*n* = 3/group). The proliferative capacity and apoptotic cell population were assessed after incubation at pH 7.4 or 6.4 (*n* = 3/group) in dysadherin OE HCT116 cells (**e**) or dysadherin KO SW480 cells (**f**). **g** Immunoblot analyses of apoptosis and proliferation markers after incubation at pH 7.4 or 6.4. Here, and throughout, the numbers under each image are the fold changes in band intensity relative to the initial condition, which were determined via ImageJ. The data are shown as the means ± SEMs. *, **, and *** denote *p* < 0.05, *p* < 0.01, and *p* < 0.001, respectively. Statistical significance was assessed via one-way ANOVA with Dunnett’s multiple comparison test for analyses involving three groups. NTC nontargeting control, KO knockout, EV empty vector, OE overexpression

To investigate the relationship between an acidic TME and CRC cell growth, we cultured CRC cells at various acidic pH values. CRC cell growth decreased under acidic conditions as the pH changed (Fig. 2a, Supplementary Fig. 3a). Human tumors typically have an extracellular pH of 6.4.^34^ We assessed how dysadherin affects CRC cell behavior at pH 6.4 using dysadherin-KO SW480 cells and OE HCT116 cells generated in a previous study^27^ (Supplementary Fig. 3b). In the clonogenic and tumor formation assays, a pH shift from pH 7.4 to 6.4 reduced CRC cell survival and tumor formation. Dysadherin OE increased both parameters under acidic conditions, whereas dysadherin KO decreased these factors under the same conditions (Fig. 2b, c). In addition, we investigated how dysadherin affects the intracellular and extracellular acidity of CRC cells. Dysadherin OE neutralized acidified intracellular regions and increased extracellular acidity, whereas dysadherin KO reduced both capacities under acidic conditions (Supplementary Fig. 3c, d). To further explore the biological role of dysadherin in CRC under acidic conditions, we assessed the gene expression profiles associated with proliferation and apoptosis. Under acidic conditions, dysadherin OE increased the expression of proliferation and antiapoptosis markers while modestly suppressing the expression of proapoptotic markers. Conversely, dysadherin KO had the opposite effect. (Fig. 2d). In the proliferation and apoptosis assays, acidic changes inhibited proliferation and increased the number of apoptotic cells. Dysadherin OE promoted proliferation and reduced the number of apoptotic cells under acidic conditions, whereas dysadherin KO had the opposite effect (Fig. 2e, f). Western blot analysis further supported these findings (Fig. 2g). Additionally, we employed a dysadherin-inhibitory peptide previously developed to block its tumor-promoting effects.^27^ Treatment with this peptide reduced cell proliferation under acidic conditions. Moreover, the peptide increased the intracellular acidity and decreased the extracellular acidity under these conditions (Supplementary Fig. 3e–g).

Cancer cells accumulate lactic acid through metabolic reprogramming, contributing to acidification of the TME.^35^ Thus, we verified the effect of dysadherin on the tumorigenic capacity of CRC cells under lactic acid-induced acidic conditions. A 20 mM lactic acid concentration decreased the pH to 6.5, mimicking acidic TME conditions (Supplementary Fig. 3h). Under these conditions, dysadherin OE increased CRC cell survival and proliferation while reducing apoptosis, whereas dysadherin KO reversed these effects (Supplementary Fig. 3i, j). Collectively, these findings suggest that dysadherin increases CRC cell survival and tumorigenicity under acidic conditions, supporting its role in tumor adaptation to the acidic TME.

### Dysadherin regulates CA9 expression to modulate tumor progression in an acidic TME

**Fig. 3:**
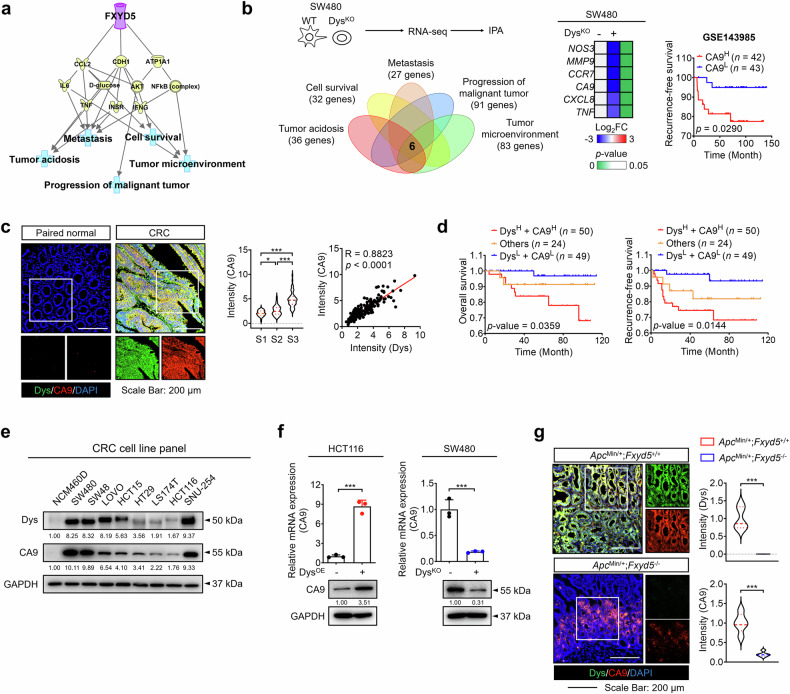
Dysadherin regulates the expression of CA9 to promote tumor progression within acidic tumors. **a** Network analysis via IPA revealed a potential link among dysadherin (*FXYD5*), the TME, tumor acidosis, cell survival, metastasis, and tumor progression. **b** IPA was performed on the mRNA sequencing data of NTC and dysadherin-KO SW480 cells. Six candidate genes related to the TME, tumor acidosis, cell survival, metastasis, and tumor progression were identified. Kaplan–Meier survival analysis of the GEO dataset (GSE143985) revealed that *CA9* was most strongly associated with poor patient survival. **c** IF analysis of dysadherin and CA9 expression in CRC patient tissues. Patients were divided into three groups according to tumor stage (S1, *n* = 45; S2, *n* = 62; S3, *n* = 61; total, *n* = 168). The graphs show the intensity of CA9 and the correlation between dysadherin and CA9. **d** Kaplan–Meier survival analysis was performed on patients with stage II and III CRC. Patients were divided into three groups on the basis of dysadherin and CA9 expression: high (*n* = 50), low (*n* = 49), and others (*n* = 24), for a total of 123 patients. Statistical significance was determined by log-rank tests. **e** Immunoblot analyses of dysadherin and CA9 in a panel of human CRC and normal colon cell lines. **f** qPCR and immunoblotting analysis of dysadherin and CA9 expression in dysadherin-OE HCT116 and dysadherin-KO SW480 cell lines. **g** IF analysis of dysadherin and CA9 in intestinal tumor tissue from 24-week-old *Apc*^Min/+^; *Fxyd*5^+/+^ and *Apc*^Min/+^; *Fxyd*5^−/−^ mice (*n* = 5/group). The data are shown as the means ± SEMs. *, **, and *** denote *p* < 0.05, *p* < 0.01, and *p* < 0.001, respectively. Statistical significance was assessed via unpaired two-tailed Student’s t-tests for two-group comparisons and one-way ANOVA with Dunnett’s multiple comparison test for analyses involving three groups

To gain further mechanistic insights into the function of dysadherin in the acidic TME, we first conducted network analysis via Ingenuity Pathway Analysis (IPA). Dysadherin was linked to the TME, tumor acidosis, cell survival, metastasis, and tumor progression (Fig. 3a). On the basis of RNA-seq data from dysadherin-KO SW480 cells,^27^ we identified six key central genes involved in these processes via IPA (Fig. 3b). To determine their prognostic value, we performed RFS analysis using the Gene Expression Omnibus (GEO) dataset (GSE143985). *CA9* showed the strongest association with poor patient prognosis, and its expression was significantly reduced in dysadherin-KO SW480 cells (Fig. 3b, Supplementary Fig. 4a). CA9 is known to catalyze the conversion of carbon dioxide and water to bicarbonate and hydrogen ions to produce HCO_3_^-^, which attenuates intracellular acidification.^36^ CRC patient tissue analysis confirmed that CA9 expression progressively increased with increasing CRC stage and was positively correlated with dysadherin expression (Fig. 3c, Supplementary Fig. 4b–d). In line with these findings, *CA9* expression showed a significantly positive correlation with *FXYD5*, as well as a consistently positive association with a tumor acidosis-related gene signature across multiple datasets. (Supplementary Fig. 4e). Additionally, high CA9 expression was associated with shorter RFS and OS (Supplementary Fig. 4f). Combined analysis revealed that patients with high levels of both dysadherin and CA9 presented significantly poorer RFS and OS than patients in the other groups did (Fig. 3d). These findings were further supported by analysis of an open-source dataset (GSE143985, TCGA) (Supplementary Fig. 4g).

Additionally, dysadherin and CA9 were expressed in CRC cells but not in normal colon cells (NCM460D). Densitometric analysis of the immunoblot bands confirmed a statistically significant positive correlation between their expression levels (*R* = 0.8422, *p* = 0.0005; Fig. 3e, Supplementary Fig. 4h). We also noted slight variation in the apparent molecular weight of dysadherin across different CRC cell lines (Fig. 3e). Since no alternative splicing of the *FXYD5* transcript was detected and no isoforms have been reported in public databases, this variation is likely attributable to differences in O-glycosylation, which is consistent with previous reports on the glycoprotein nature of dysadherin.^29,37^ When dysadherin expression was depleted, the expression of CA9 was reduced. Conversely, the upregulation of dysadherin notably increased the expression of CA9 (Fig. 3f). Further validation via dysadherin knockdown (KD) using the most effective siRNA sequence (#3) confirmed CA9 downregulation in SNU-254 cells (rectal cancer) and patient-derived primary cells (Table S3) (Supplementary Fig. 4i–k). In addition, Dysadherin KD by shRNA decreased CA9 expression in SK-Hep-1 liver cancer cells and MDA-MB-231 breast cancer cells. (Supplementary Fig. 4l). Furthermore, immunofluorescence (IF) analysis revealed that compared with control (*Apc*^Min*/+*^) mice, dysadherin-KO (*Fxyd5*^−/−^) mice presented lower CA9 expression in primary tumor lesions (Fig. 3g, Supplementary Fig. 4m). Taken together, our findings suggest that dysadherin expression regulates CA9 expression across multiple cancer types, highlighting its role in tumor adaptation to the acidic TME.

### Dysadherin induces CA9 expression through the integrin/FAK/STAT3 axis

**Fig. 4:**
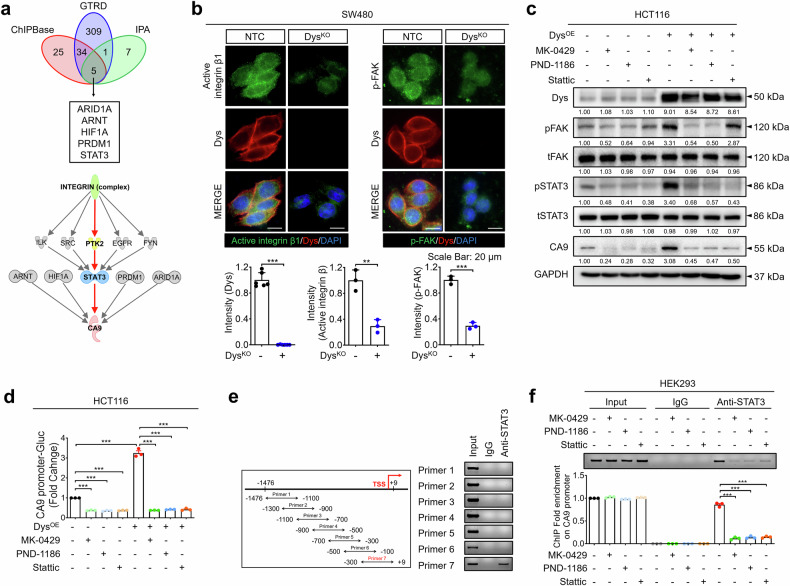
Dysadherin increases the transcriptional activity of CA9 by activating the integrin/FAK/STAT3 axis. **a** Venn diagram showing five potential transcription factors of CA9. Downstream analysis by IPA revealed that the integrin/FAK (*PTK2*) axis activated STAT3. **b** IF analysis of dysadherin, active integrin β1, and p-FAK in dysadherin KO SW480 cells. Antibody clones used for integrin β1 detection: 12G10, which recognizes specific conformational epitopes of human integrin β1. **c** Immunoblot analyses of dysadherin, p-FAK, t-FAK, p-STAT3, t-STAT3, and CA9 in dysadherin-OE HCT116 cells treated with or without 0.1 µM MK-0429, 1 µM PND-1186 or 1 µM Stattic. **d** The promoter activity of CA9 upon dysadherin OE with or without MK-0429, PND-1186 or Stattic treatment was tested via a luciferase reporter assay. The total transcript level was normalized to the β-galactosidase transcript level and is presented as the fold change with respect to that in nontreated HCT116 cells (*n* = 3/group). **e** Potential binding site for p-STAT3 in the CA9 promoter region identified by a ChIP assay. The CA9 promoter region was fragmented into 7 segments and immunoprecipitated with an anti-p-STAT3 antibody. **f** The binding affinity of p-STAT3 for the CA9 promoter with or without MK-0429, PND-1186 or Stattic was tested via a ChIP assay (*n* = 3/group). The data are shown as the means ± SEMs. *, **, and *** denote *p* < 0.05, *p* < 0.01, and *p* < 0.001, respectively. Statistical significance was assessed via unpaired two-tailed Student’s *t* tests for two-group comparisons and one-way ANOVA with Dunnett’s multiple comparison test for analyses involving three groups

To gain further mechanistic insight, we used three analytical tools (ChipBase, GTRD, and IPA) and identified five potential transcription factors for *CA9* (Fig. 4a). In a previous study, we demonstrated that dysadherin enhances cancer cell adhesion to fibronectin, thereby activating integrin/FAK signaling.^27^ IPA analysis identified STAT3 as a downstream effector of the integrin/FAK pathway^38^ (Fig. 4a). This dysadherin/FAK/STAT3 axis was further validated by IF and immunoblot analysis of dysadherin OE, KO, and KD CRC cells (Fig. 4b, Supplementary Fig. 5a). Although HIF-1α was also suggested by IPA to be a potential upstream regulator of CA9, its protein levels were unaffected by dysadherin expression (Supplementary Fig. 5a), suggesting that dysadherin regulates CA9 independently of HIF-1α. Furthermore, IF analysis revealed that *Fxyd5* KO attenuated integrin activation and reduced the expression of p-FAK, p-STAT3, and CA9 in tumors from *Apc*^Min*/+*^ mice (Supplementary Fig. 5b). To verify whether fibronectin stimulation regulates CA9 through this axis, we performed immunoblot and luciferase reporter assays following treatment with fibronectin, the integrin inhibitor MK-0429, the FAK inhibitor PND-1186, the STAT3 inhibitor Stattic, and a STAT3-targeting siRNA (siSTAT3 #1), which presented the highest knockdown efficiency (Supplementary Fig. 5c, d). We first determined the half-maximal inhibitory concentrations (IC_50_) of the indicated inhibitors to ensure minimal cytotoxicity and to select appropriate doses for subsequent mechanistic experiments (Supplementary Fig. 5c). Fibronectin stimulation increased p-FAK, p-STAT3, and CA9 expression in dysadherin-OE cells, but not in dysadherin-KO cells (Supplementary Fig. 5e). Dysadherin OE increased p-STAT3 and CA9 expression, both of which were significantly reduced by treatment with MK-0429, PND-1186, Stattic or siSTAT3 #1 (Fig. 4c, Supplementary Fig. 5f). In line with these results, dysadherin-induced CA9 promoter activity was abolished by the same inhibitors (Fig. 4d, Supplementary Fig. 5g). Chromatin immunoprecipitation (ChIP) assays further demonstrated that STAT3 directly binds to the CA9 promoter region, and this binding affinity decreased after treatment with MK-0429, PND-1186, and Stattic (Fig. 4e, f). IF and immunoblot analyses revealed that the dysadherin-inhibitory peptide significantly reduced dysadherin-mediated activation of the integrin/FAK/STAT3 axis and CA9 expression (Supplementary Fig. 5h, i). Functionally, treatment with these inhibitors increased the intracellular acidity and decreased the extracellular acidity under acidic conditions, thereby suppressing cell proliferation (Supplementary Fig. 5j–l). Collectively, these findings demonstrate that dysadherin promotes CA9 transcription through the fibronectin/integrin/FAK/STAT3 signaling axis. Additionally, exposure to acidic pH (6.4) increased both the mRNA and protein levels of dysadherin, suggesting that extracellular acidosis may transcriptionally upregulate dysadherin (Supplementary Fig. 5m, n). We also observed elevated expression of p-FAK, p-STAT3, and CA9 under acidic conditions (Supplementary Fig. 5n). Under both neutral and acidic pH conditions, dysadherin OE increased the expression of these markers. These findings indicate that the dysadherin/CA9 axis is activated in response to acidic stress, and that dysadherin is both necessary and sufficient to drive the integrin/FAK/STAT3/CA9 signaling pathway in this context.

### The dysadherin/CA9 axis facilitates CRC cell adaptation and malignancy in an acidic TME

**Fig. 5:**
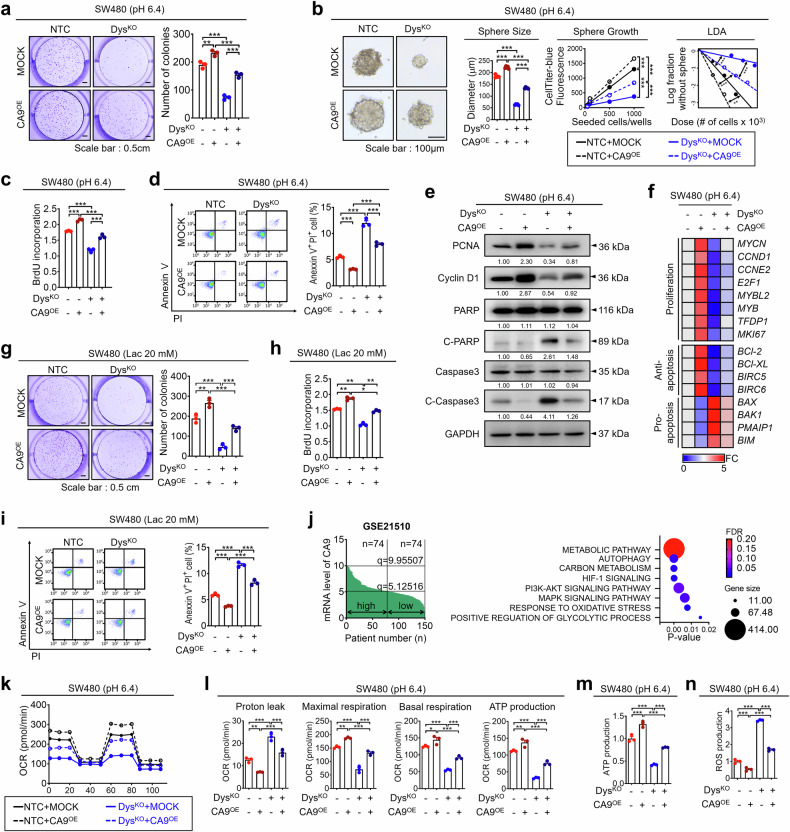
The dysadherin/CA9 axis promotes CRC cell adaptation and increases cell survival within an acidic TME. The survival potential (**a**), self-renewal properties (**b**), proliferative capacity (**c**), and apoptotic cell population (**d**) were assessed in NTC or dysadherin KO SW480 cells with CA9 OE at pH 6.4 (*n* = 3/group). Immunoblot analyses (**e**) and heatmaps comparing the relative expression (**f**) of apoptosis and proliferation markers in NTC or dysadherin KO SW480 cells with CA9 OE at pH 6.4 (*n* = 3/group). The survival potential (**g**), proliferative capacity (**h**), and apoptotic cell population (**i**) were assessed in NTC or dysadherin KO SW480 cells with CA9 OE under lactate-induced acidic conditions. **j** Left: CA9 expression levels in CRC patient samples from the GEO dataset GSE21510, with patients stratified into high and low groups on the basis of the median CA9 expression value. (CA9^high^, *n* = 74; CA9^low^, *n* = 74). Right: DAVID analysis of DEGs between the CA9-high and CA9-low cohorts. OCR profiles (**k**, **l**) and ATP production (**m**) were monitored in NTC or dysadherin KO SW480 cells with CA9 OE at pH 6.4 (*n* = 3/group). **n** Flow cytometric analysis of intracellular ROS production in NTC or dysadherin KO SW480 cells with CA9 OE at pH 6.4 (*n* = 3/group). The data are shown as the means ± SEMs. *, **, and *** denote *p* < 0.05, *p* < 0.01, and *p* < 0.001, respectively. Statistical significance was assessed via one-way ANOVA with Dunnett’s multiple comparison test for analyses involving three groups

To investigate the role of CA9 in CRC cells and patient-derived primary cells under various pH conditions, we first observed that CA9 manipulation had no effect on cell proliferation or apoptosis at pH 7.4. However, under acidic conditions, CA9 OE promoted proliferation and reduced apoptosis, whereas CA9 KD via the most effective siRNA sequence (#2) had the opposite effect (Supplementary Fig. 6a, b). Next, to validate the effect of the dysadherin/CA9 axis on CRC tumorigenicity under acidic conditions, we generated CA9 OE cell lines from dysadherin KO-SW480 cells (Supplementary Fig. 6c). Clonogenic and tumor formation assays revealed that dysadherin KO reduced cell survival and tumor formation under acidic conditions, whereas CA9 OE reversed these effects (Fig. 5a, b). We also assessed the intracellular and extracellular acidity. Dysadherin KO increased intracellular acidity and reduced extracellular acidity, whereas CA9 OE reversed these effects (Supplementary Fig. 6d, e). Proton exporters are essential for maintaining the intracellular pH by extruding protons across the plasma membrane.^39^ However, their expression remained unchanged upon subsequent modulation of the dysadherin/CA9 axis, suggesting that this pathway regulates pH independently of direct effects on proton exporter expression (Supplementary Fig. 6f). Proliferation and apoptosis assays confirmed that dysadherin KO decreased proliferation and increased apoptosis under acidic conditions, whereas CA9 OE reversed these effects (Fig. 5c, d). Conversely, dysadherin OE promoted proliferation and reduced apoptosis, effects that were reversed by CA9 KD (Supplementary Fig. 6g). Western blot and transcriptional analyses further supported these findings by demonstrating that dysadherin KO reduced the expression of proliferation and antiapoptotic markers while modestly increasing the expression of proapoptotic genes under acidic conditions. These alterations were partially reversed by CA9 OE (Fig. 5e, f). Similarly, under lactic acid-induced acidic conditions, dysadherin KO reduced survival and proliferation while increasing apoptosis. These effects were reversed by CA9 OE (Fig. 5g–i). To better model preclinical conditions, we cultured tumor spheroids of SW480 and patient-derived primary CRC cells in Matrigel.^40^ Dysadherin KO or KD reduced tumor spheroid growth under acidic conditions, whereas CA9 OE reversed this phenotype. Furthermore, dysadherin KO or KD increased intracellular acidity and decreased extracellular acidity, both of which were reversed by CA9 OE (Supplementary Fig. 6h). Importantly, these pH alterations were also observed in size-matched spheroids, confirming that these effects were independent of spheroid size.

To further explore the dysadherin/CA9 axis in CRC, we analyzed *CA9* expression in cancer (GSE21510) and stratified 156 CRC patient samples into high- and low-*CA9* expression groups on the basis of the median expression value (Fig. 5j). Using DEGs from the high *CA9* expression group, we performed a Database for Annotation, Visualization, and Integrated Discovery (DAVID) analysis, which revealed significant enrichment of energy metabolism pathway-related signatures, including those related to carbon metabolism, the oxidative stress response, and glycolysis (Fig. 5j). Several key genes associated with enhanced energy metabolism, such as *PKM*, *PFKP* and *IDH*, were consistently upregulated in high CA9-expressing tumors (Table S4). To investigate the role of the dysadherin/CA9 axis in metabolic regulation under acidic stress, we analyzed energy metabolism-related gene expression in SW480 cells cultured under neutral (pH 7.4) and acidic (pH 6.4) conditions. Acidic stress increased gene expression, which was suppressed by dysadherin KO under both conditions. This suppression was partially reversed by CA9 OE, suggesting that the dysadherin/CA9 axis promotes metabolic gene expression in response to acidic stress. (Supplementary Fig. 7a). We further performed bioenergetic profiling of CRC cells under neutral and acidic conditions. Under acidic stress (pH 6.4), dysadherin KO reduced the oxygen consumption rate (OCR), extracellular acidification rate (ECAR), and ATP production, whereas CA9 OE reversed these changes (Fig. 5k–m, Supplementary Fig. 7b). At neutral pH, modest decreases in the ECAR and OCR were also observed in dysadherin-KO cells, likely reflecting cell survival-related pathways^26^ (Supplementary Fig. 7c). In contrast, CA9 modulation had little effect on the OCR or ECAR under neutral conditions (Supplementary Fig. 7c, d). These results suggest that CA9 contributes to metabolic reprogramming specifically under acidic stress, thereby facilitating CRC cell adaptation and survival. Furthermore, we examined whether glycolytic activity contributes to the dysadherin/CA9 axis. Treatment with 2-DG markedly reduced the ECAR but had no effect on dysadherin OE-induced increases in p-FAK, p-STAT3, or CA9 levels. In contrast, inhibition of FAK or STAT3 suppressed CA9 expression and reversed the increase in the ECAR associated with dysadherin OE under acidic conditions (Supplementary Fig. 7e). These results indicate that CA9 regulation by dysadherin is dependent on FAK/STAT3 signaling and occurs independently of glycolysis. Additionally, FACS analysis revealed that dysadherin KO increased intracellular reactive oxygen species (ROS) levels under acidic conditions, whereas the reintroduction of CA9 mitigated this effect (Fig. 5n, Supplementary Fig. 7f). Mitochondrial ROS showed a similar trend (Supplementary Fig. 7g). An acidic TME has been reported to activate cancer-associated fibroblasts (CAFs), which promote angiogenesis, ECM remodeling, and immunosuppression, ultimately contributing to tumor progression.^41,42^ To determine whether the dysadherin/CA9 axis influences CAF activation, we examined a CAF-associated gene signature comprising genes involved in angiogenesis, ECM remodeling, and immunosuppression. This gene signature was significantly upregulated when CAFs were cultured under acidic conditions (Supplementary Fig. 7h). Similarly, conditioned media (CM) from dysadherin-OE HCT116 cells also induced the expression of this gene signature. In contrast, these changes were reversed when CAFs were treated with CM collected from CA9 KD/dysadherin-OE HCT116 cells (Supplementary Fig. 7i). Furthermore, to evaluate the role of the dysadherin/CA9 axis in tumor immune evasion, we assessed T-cell responses in a coculture system using CM from HCT116 cells. Given that acidic conditions impair T-cell activation and function,^43,44^ we hypothesized that dysadherin-driven acidosis contributes to immune suppression. CM from dysadherin-OE HCT116 cells suppressed T-cell responses, as evidenced by reduced CD69 and granzyme B expression and increased PD-1 levels. These immunosuppressive effects were restored when T cells were treated with CM from CA9 KD/dysadherin OE HCT116 cells (Supplementary Fig. 7j, k). Taken together, our findings suggest that the dysadherin/CA9 axis facilitates CRC cell adaptation to an acidic TME by promoting metabolic reprogramming, activating CAFs, and enabling immune evasion, thereby contributing to enhanced tumor malignancy.

### The dysadherin/CA9 axis drives CRC adaptation to the acidic TME and promotes metastasis

**Fig. 6:**
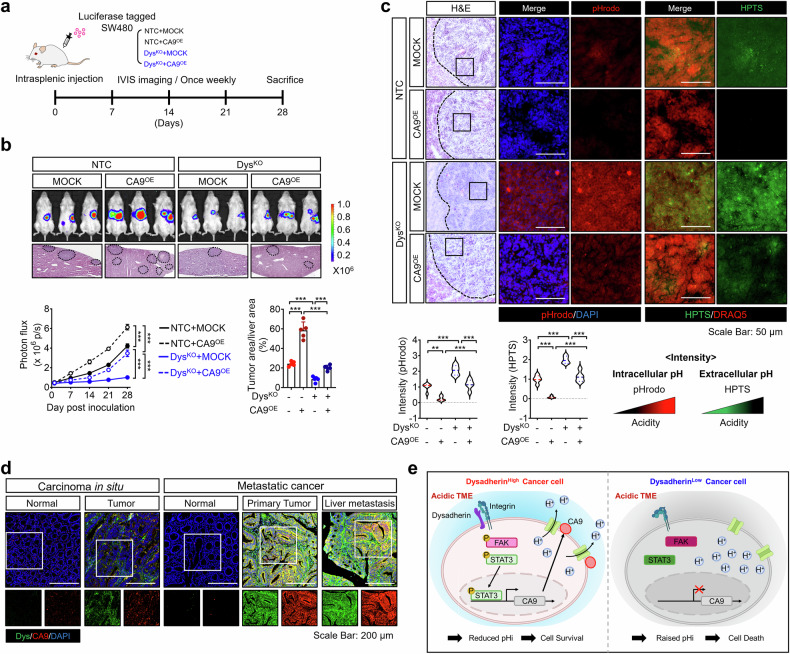
The dysadherin/CA9 axis facilitates metastasis by promoting CRC cell adaptation to the acidic TME. **a** Experimental scheme of the intrasplenic injection mouse model. Splenectomy was performed at the time of tumor cell injection. **b** Top: Representative bioluminescence images of mice bearing luciferase-labeled SW480 cells, comparing NTC controls with dysadherin KO SW480 cells overexpressing CA9 (*n* = 5/group). Middle: Hematoxylin and eosin-stained liver sections from mice bearing metastatic lesions. Bottom: Quantitative plots depicting photon flux and the metastatic nodule area corresponding to the images shown. **c** pHrodo and HTPS staining of metastatic nodules in the mouse liver (*n* = 5/group). The graphs show the intensities of pHrodo and HPTS in the indicated groups. **d** IF analysis of dysadherin and CA9 expression in the carcinoma in situ and metastatic cancer tissues of CRC patients (*n* = 10/group). **e** Schematic illustration summarizing the study, highlighting the contribution of the dysadherin/CA9 axis to CRC progression in acidic conditions. Created with BioRender.com. The data are presented as the means ± SEMs. *, **, and *** denote *p* < 0.05, *p* < 0.01, and *p* < 0.001, respectively. Statistical significance was assessed via two-way ANOVA with Bonferroni’s multiple comparison test or one-way ANOVA with Dunnett’s multiple comparison test for analyses involving more than three groups

To investigate the role of the dysadherin/CA9 axis in CRC metastatic potential, we employed an intrasplenic injection mouse model, a widely used approach, to analyze the key steps of metastasis and distant organ colonization (Fig. 6a). In vivo imaging and histological analyses revealed that dysadherin KO significantly reduced the incidence and burden of hepatic metastasis in CRC cells, whereas these changes were reversed by CA9 OE (Fig. 6b). To functionally support these in vivo findings, we evaluated cell motility and invasiveness under neutral and acidic stress. Wound healing and transwell assays, which were performed with or without Matrigel-coated membranes, revealed that dysadherin OE increased CRC cell migration and invasion under acidic conditions, whereas dysadherin KO impaired both properties (Supplementary Fig. 8a). Only modest increases in motility were observed at neutral pH, likely reflecting pH-independent mechanisms such as cytoskeletal remodeling or reduced cell–cell adhesion.^26,29^ Importantly, CA9 OE rescued the impaired migratory and invasive phenotypes of dysadherin-KO cells under acidic conditions (Supplementary Fig. 8b), whereas CA9 modulation had little effect at pH 7.4. In line with these observations, IF staining revealed that dysadherin KO increased intracellular acidity and reduced extracellular acidity in metastatic lesions, while CA9 OE restored extracellular acidity and reduced intracellular acidity (Fig. 6c). Consistent with the in vitro findings, proton exporter expression remained unchanged (Supplementary Fig. 9a). Moreover, transcriptional profiling revealed that dysadherin KO downregulated the expression of metastasis- and proliferation-related genes while increasing the expression of apoptosis-related genes. These changes were reversed by CA9 OE (Supplementary Fig. 9b). Additionally, we confirmed the presence of the dysadherin/CA9 axis in this model, which revealed that dysadherin KO attenuated integrin activation and reduced the expression of p-FAK, p-STAT3 and CA9, whereas CA9 OE reversed these alterations (Supplementary Fig. 9c). These results support the idea that CA9 may enhance integrin signaling and promote dysadherin expression through acidification, forming a reinforcing feedback loop between dysadherin and CA9. Moreover, dysadherin KO reduced the levels of α-SMA and collagen I in metastatic lesions, whereas CA9 overexpression restored their levels (Supplementary Fig. 9d).

We also examined dysadherin and CA9 expression in carcinoma in situ and in metastatic CRC, including both primary tumors and liver metastases (Table S5). Both proteins were more highly expressed in metastatic CRC than in carcinoma in situ (Fig. 6d, Supplementary Fig. 9e). Together, these findings confirm that high dysadherin expression is positively associated with CA9 and CRC metastasis. Overall, our results suggest that the dysadherin/CA9 axis promotes adaptation to acidic microenvironments, thereby enhancing tumor growth and metastatic potential (Fig. 6e, graphic summary).

## Discussion

Dysadherin (FXYD5) is a multifunctional protein involved in tumor-promoting processes, including the regulation of Na⁺/K⁺-ATPase activity, the induction of EMT, the modulation of immune responses, and the enhancement of fibronectin-mediated adhesion, which activates integrin/FAK signaling to support migration and survival.^26,29,31,33^ Our study reveals a previously unrecognized role of dysadherin in enabling CRC adaptation to an acidic TME. Integrated genomic and biological analyses identified tumor acidity as a hallmark of CRC progression, which is positively correlated with dysadherin expression. Mechanistically, dysadherin enhances survival and proliferation under acidic stress by upregulating CA9 through the integrin/FAK/STAT3 pathway. In addition to its catalytic role in extracellular CO₂ hydration, CA9-mediated acidification can influence integrin clustering, ECM remodeling, and related signaling, potentially explaining its ability to restore FAK/STAT3 activation in dysadherin-deficient cells. This newly defined dysadherin/CA9 axis highlights how tumors exploit acidosis to drive malignancy, further extending the functional significance of dysadherin in CRC progression.

Solid tumors frequently exhibit an acidic TME due to metabolic reprogramming during progression.^15,16^ In response to acidic conditions, highly malignant tumors develop adaptive mechanisms for survival. Clinical studies have shown that high expression of acidosis-related markers in tumors is linked to poor prognosis, underscoring tumor acidity as an appealing target for diagnostic and therapeutic strategies.^1,15,21,22^ In this study, trajectory analysis via single-cell genomic data from CRC patients revealed that tumor acidosis, dysadherin expression, and CRC progression are closely linked, collectively contributing to cancer progression (Fig. 1b). We found that the expression of dysadherin and acidosis-related genes in tumors increased with CRC progression, indicating a largely consistent positive correlation in patient tissues (Fig. 1b–d, Supplementary Fig. 1d, f). Additionally, CRC patients with high dysadherin and LAMP2 expression exhibited poorer prognoses (Fig. 1e, Supplementary Fig. 1h). Functional analyses of CRC cells with either dysadherin overexpression or silencing confirmed that high dysadherin levels increase the incidence of malignant phenotypes, especially under acidic conditions (Fig. 2b–f). Taken together, these findings establish tumor acidity as a hallmark of CRC progression and highlight the critical role of dysadherin.

Dysadherin is expressed predominantly in malignant epithelial cells, with minimal expression in normal or stromal compartments, as supported by both previous studies and our immunofluorescence data (Supplementary Fig. 2a).^23,25,33^ To further assess the stromal contribution, coculture of MC38 cells with T cells from WT or *Fxyd5*^−/−^ mice resulted in no differences in tumor growth, whereas ex vivo T-cells from both type of mice exhibited similar activation and cytokine production, which is in line with previous observations^27^ (Supplementary Fig. 10a–-c). These results reinforce the notion that FXYD5 primarily acts as a tumor cell–intrinsic mediator of adaptation to acidic stress. Notably, acidic stress may upregulate dysadherin via the activation of pH-responsive transcription factors such as HIF-1α and NF-κB, for which putative binding sites were identified in the FXYD5 promoter region (Supplementary Fig. 11).^45,46^ Furthermore, the strong positive correlation between FXYD5 and multiple acidosis-associated genes supports its broader role in facilitating tumor adaptation to acidic microenvironments.

Cancer cells undergo extensive molecular and phenotypic changes in response to adverse environments, allowing adaptation and survival. CA9 is a well-known responder to hostile conditions, and its expression is induced by hypoxia and acidosis.^36^ However, the precise mechanisms underlying CA9 regulation remain incompletely understood. In this study, we provide new mechanistic insights into CA9 regulation in malignant tumors. We identified CA9 as a dysadherin target that is upregulated in malignant tumors and is positively correlated with CRC stage. Patients with high CA9 and dysadherin expression had a worse prognosis (Fig. 3b–d, Supplementary Fig. 4f, g). Additionally, we identified STAT3, HIF1A, PRDM1, ARNT and ARID1A as transcriptional regulators of CA9 via bioinformatic analyses. Notably, dysadherin promotes oncogenic phenotypes by activating integrin/FAK signaling, which in turn triggers downstream effectors.^27,47^ IPA revealed that among the five transcription factors, STAT3 is preferentially activated as a downstream mediator of integrin/FAK signaling. Therefore, we demonstrated that dysadherin induces CA9 transcriptional activation via STAT3 signaling (Fig. 4a). We also confirmed this signaling axis in a xenograft model (Supplementary Fig. 9c). Interestingly, CA9 OE reversed the reduction in p-FAK and p-STAT3 levels observed under dysadherin KO conditions. These findings suggest that CA9 may modulate the integrin/FAK/STAT3 signaling pathway in a dysadherin-independent manner, possibly through membrane acidification–mediated integrin activation^48^ or direct protein–protein interactions. We also confirmed the dysadherin/integrin/FAK/STAT3/CA9 axis via the use of a dysadherin-inhibitory peptide and pharmacologic inhibitors of integrin, FAK, and STAT3 (Fig. 4b–f, Supplementary Fig. 5h, i). For the first time, our study establishes a mechanistic link between dysadherin expression and increased STAT3 activation, leading to CA9 upregulation. Moreover, treatment with these inhibitors disrupts CRC cell adaptation to acidic stress by interfering with tumor acidity regulation, suggesting a novel acid-modulating role for anticancer drugs that target integrin, FAK, and STAT3. These findings expand the therapeutic potential of these agents beyond their conventional applications.

Tumor cells maintain the intracellular pH in an acidic TME by exporting protons through multiple transporters and exchange.^16^ CA9 converts CO_2_ into bicarbonate and protons; bicarbonate buffers the intracellular acidity, whereas protons acidify the TME.^36^ Our data showed that the dysadherin/CA9 axis promotes CRC survival under acidic stress. The correlations among FXYD5, LAMP2, and CA9 suggest coordinated regulation of pH homeostasis. Broader profiling of monocarboxylate transporters (MCTs), Na^+^/H^+^ exchangers (NHEs), Na^+^/HCO_3_^−^ cotransporters (NBCs), and vacuolar-type ATPase (V-ATPase) subunits revealed that only CA9 was consistently induced by FXYD5 (Supplementary Fig. 6f, 9a). Thus, CA9 has emerged as a central downstream effector of dysadherin. However, auxiliary regulators, including lysosomal pathways, may contribute in a context-dependent manner through transporter trafficking or posttranscriptional control. Overall, the FXYD5/CA9 axis appears to be dominant but likely operates within a broader, partially redundant network sustaining TME acidity during CRC progression. Proton dynamics intrinsically and extrinsically influence cellular function. Intracellular pH neutralization alters metabolic pathways, as shown by bioinformatics and experimental analyses, indicating that CA9 upregulation drives metabolic reprogramming (Fig. 5j–n). In parallel, extracellular acidification stimulates CAFs, promoting angiogenesis, ECM remodeling, and immunosuppression (Supplementary Fig. 7h, i).^49–53^ These findings highlight a broader oncogenic role for CA9 beyond pH regulation, suggesting that a deeper investigation into the extrinsic effects of CA9 could provide novel insights into CRC malignancy in acidic TMEs.

Despite the strengths of this study, several limitations should be acknowledged. First, we employed a global Fxyd5-KO model in *Apc*^Min/+^ mice to study the role of dysadherin in vivo. Although this model clearly demonstrated the impact of dysadherin depletion on tumor acidity and progression, it does not distinguish tumor cell–intrinsic effects from those mediated by stromal or immune cells. Our additional T-cell functional assays and coculture experiments suggest that dysadherin does not markedly affect T-cell–mediated antitumor activity (Supplementary Fig. 10a–c), but definitive confirmation will require future studies using tissue- or immune cell–specific KO models. Second, although we demonstrated the functional significance of the dysadherin/CA9 axis in vitro and in vivo, therapeutic validation in colorectal cancer models remains limited. To address this partially, we conducted experiments using a dysadherin-inhibitory peptide in 3D tumor spheroids, which better recapitulates features of the TME. Treatment with this peptide reduced CA9 expression, increased intracellular acidity, and suppressed spheroid growth under acidic conditions (Supplementary Fig. 12). Together, these findings support the translational potential of targeting the dysadherin/CA9 axis and highlight the need for further in vivo validation, including in orthotopic or patient-derived xenograft (PDX) models and evaluation of its potential to overcome acidity-associated therapy resistance.

Overall, our study demonstrated that tumor cells with a highly active dysadherin/CA9 axis exploit acidosis to drive malignancy. We identified dysadherin as a key regulator of tumor adaptation to adverse microenvironments during malignant progression. These findings may lead to new therapeutic approaches that target the dysadherin/CA9 axis to more effectively inhibit CRC progression.

## Materials and methods

### Ethical approval

In vivo experiments were conducted with prior approval from the Institutional Animal Care and Use Committee (IACUC) at the Gwangju Institute of Science and Technology (GIST; No. GIST2021-065). The evaluation of dysadherin, CA9, and LAMP2 expression in CRC patient samples was conducted under prior approval from the GIST Institutional Review Board (No. 20200108-BR-50-07-02, No. 20210115-BR-58-03-02). Biospecimens and associated data were obtained from the Biobank of Chonnam National University Hwasun Hospital, which is part of the Korea Biobank Network. Experiments involving human PBMCs were conducted in accordance with the guidelines and approval of the Institutional Review Board of the National Cancer Center (IRB No. NCC2022-0198). All procedures involving human tissues adhered to the principles of the Declaration of Helsinki, and written informed consent was obtained from all participants prior to inclusion in the study. All in vitro experiments were repeated at least three times independently. Exclusion criteria were not applied in this study; thus, outliers were included in all experimental results.

### Animal models

*Fxyd5*^−/−^ mice were generated as previously described.^27^ Using CRISPR/Cas9 gene-editing technology, three guide RNAs (gRNA sequences: AGGCTGCTAGGCATCTCGGGGGG, TCTTCCTGGGCTCG GTCACGTGG, and CCCCGATGAGCGATACAGAGACA) were designed to target introns 1 and 7 of the Fxyd5 gene, enabling the deletion of exons 2–7, which encompass the ATG start codon and most of the coding region. We developed a murine model by crossing female *Fxyd5*^−/−^ mice with male *Apc*^Min/+^ mice on a C57BL/6 J background. *Apc*^Min/+^ mice were obtained from Jackson Laboratory (Bar Harbor, ME, USA), and *Fxyd5*^*-/-*^ mice were obtained from Vitalstar (Beijing, China). Intestinal tumors were categorized into three groups on the basis of their diameter: small (<3 mm), medium (≥3 mm and <5 mm), and large (≥5 mm). The tumor load was calculated via a weighted scoring method as previously described^27,54^:

Tumor load = (number of small-sized tumors × 1) + (number of medium-sized tumors × 2) + (number of large-sized tumors × 3).

A splenic injection model was employed to recapitulate the physiological process of metastatic dissemination and colonization in distant organs.^55^ For this model, cells were labeled with luciferase (pCMV-luc) and injected into the spleens of NSG mice (NOD. Cg-Prkdc^scid^ Il2rg^tm1Wjl^/SzJ, #005557, Jackson Laboratory, Bar Harbor, Maine, U.S), followed by splenectomy (1 × 10^6^ cells/mouse). The surviving cells that grow in the liver then contribute to liver metastasis formation. We monitored liver metastatic progression weekly for 28 days by measuring luciferase activity via the IVIS Lumina III in vivo imaging system (PerkinElmer, Waltham, MA, USA). At the end of the experiment, the mice were sacrificed, and their livers were excised for assessment of metastasis.

### Cell lines

The human CRC cell lines HCT116 (Cat# 10247, RRID: CVCL_0291), SW480 (Cat# 10228, RRID: CVCL_0546), LoVo (Cat# 10229, RRID: CVCL_0399), HCT15 (Cat# 10225, RRID: CVCL_0292), LS174T (Cat# 10188, RRID: CVCL_1384), HT29 (Cat# 30038, RRID: CVCL_0320), SNU-254 (Cat# 00254.1, RRID: CVCL_5041), and human T lymphocyte Jurkat cell (Cat# 40152) were purchased from the Korean Cell Line Bank (KCLB, Seoul, Republic of Korea). The SW48 cell line (Cat# CCL-231, RRID: CVCL_1724) was purchased from the American Type Culture Collection (ATCC, Rockville, MD, USA). The normal colon cell line NCM460D (Cat# NCM460D, RRID: CVCL_IS47) was purchased from INCELL (San Antonio, TX, USA). The MC38 murine intestinal tumor cell line (C57BL/6 background; RRID: CVCL_B288) was purchased from Kerafast (Boston, MA, USA). All the cell lines were cultured according to the supplier’s instructions. Additionally, we used dysadherin OE HCT116 cells, KO SW480 cells, KD MDA-MB-231 cells, and KO SK-HEP-1 cells, which were established in a previous study.^26,27,56^ All the cells were cultured following the supplier’s instructions. All experiments were performed within 20 passages from the first thaw, and the cells were routinely tested for mycoplasma contamination using an e-MycoTM mycoplasma detection kit (iNtron Biotechnology, Seongnam, Republic of Korea).

### Acidic culture media

For acidic conditions, pH-adjusted RPMI 1640 medium was obtained from Welgene (Gyeongsan, Republic of Korea). The pH 7.4 media was supplemented with L-glutamine and 25 mM 4-(2-hydroxyethyl)-1-piperazineethanesulfonic acid (HEPES) without NaHCO_3_. The pH 6.4 media was supplemented with L-glutamine and 20 mM 2-(N-morpholino) ethane sulfonic acid (MES) without NaHCO_3_. For various pH values, the pH of the media was adjusted to 6.8, 6.6, or 6.2 using 1 N HCl or 12 N NaOH.

For lactic acid-induced acidic conditions, RPMI 1640 was supplemented with L-(+)-lactic acid (Sigma‒Aldrich, St. Louis, Missouri, USA), and the pH was measured using an InLab Routine Pro pH sensor electrode (Mettler Toledo, Columbus, Ohio, US). The cells treated with pH-adjusted media were incubated at 37 °C and 5% CO_2_ for 24 h.

### pHrodo staining

A total of 8.0 × 10^3^ cells were seeded on 96-well plates and incubated at 37 °C and 5% CO_2_ for 24 h. After 24 h, the cells were treated with pH-adjusted media supplemented with 5% fetal bovine serum (FBS) and incubated at 37 °C in a CO_2_-free incubator for 24 h. The intracellular pH was measured using pHrodo™ Red AM Intracellular pH Indicator Dye (Invitrogen, Carlsbad, MA, USA). In accordance with the supplier’s instructions, the cells were incubated with pHrodo™ Red, and the excitation/emission wavelengths were read at 560/585 nm using a Varioskan LUX Multimode Microplate Reader (Thermo Fisher Scientific, Waltham, MA, USA).

### HPTS assay

A total of 8.0 × 10^3^ cells were seeded on 96-well plates and incubated at 37 °C and 5% CO_2_ for 24 h. After 24 h, the cells were treated with pH-adjusted media supplemented with 5% FBS and incubated at 37 °C in a CO_2_-free incubator. The extracellular pH was measured using HPTS (Sigma‒Aldrich). In accordance with the supplier’s instructions, the cell cultured media mixed with 50 μM HPTS, and the excitation/emission wavelengths were read at 450/510 nm using a Varioskan LUX Multimode Microplate Reader (Thermo Fisher Scientific).

### Fluorescence imaging and pH measurement in tumor tissues

Frozen tumor sections (10 μm) were stained with HPTS (100 μM) for extracellular pH and DRAQ5 (5 μM) for nuclear staining. Imaging was performed on a Leica SP8 microscope using sequential scanning and spectral separation (HPTS: Ex 450 nm/Em 500–550 nm; DRAQ5: Ex 647 nm/Em 665–750 nm). No bleed-through was observed in single-stain controls.

For pH calibration, sections were incubated in HEPES-buffered solutions (pH 6.2–7.4) with 150 mM NaCl and 5 mM glucose. Under these imaging conditions, HPTS fluorescence intensity increased with extracellular pH, confirming the probe’s sensitivity for assessing extracellular pH in our experimental setup.^57^

### Statistical analyses

The data are presented as the means ± standard errors of the means (SEMs). Statistical comparisons between two groups were performed via Student’s *t* test or two-way ANOVA followed by Bonferroni’s post hoc multiple comparison test. For comparisons among three or more groups, one-way ANOVA with Dunnett’s multiple comparison test or two-way ANOVA with Bonferroni post hoc correction was performed via GraphPad Prism (GraphPad Software, Inc., San Diego, CA, USA). The log-rank test was used for Kaplan‒Meier analysis via GraphPad Prism. Pearson’s correlation coefficient (R) was used to assess the linear relationship. The number of biological replicates for each experiment is indicated in the corresponding figure legends. Statistical significance is denoted by asterisks, where *, **, and *** represent *p* < 0.05, *p* < 0.01, and *p* < 0.001, respectively.

### Reporting summary

Further information on research design is available in the Nature Research Reporting Summary linked to this article.

## Supplementary information

Supplementary materials

Unprocessed Data

Supplementary table 1

Supplementary table 4

FACS gating strategy

Reporting summary

## Data Availability

All the data supporting the conclusions of this study are provided in the main figures and supplementary materials. The raw sequencing datasets generated and analyzed in this work are publicly available in the Gene Expression Omnibus (GEO, https://www.ncbi.nlm.nih.gov/geo/) under accession numbers GSE106582, GSE144735, GSE106584, GSE143985, GSE21510, and GSE18105.
