# Supplementary material for: The dysadherin/carbonic anhydrase 9 axis shapes an acidic tumor microenvironment to promote colorectal cancer progression
Source: Signal Transduct Target Ther. 2026 Jan 15;11:19. doi: 10.1038/s41392-025-02543-x (PMC12804794; doi:10.1038/s41392-025-02543-x)

Figure 2g - 1

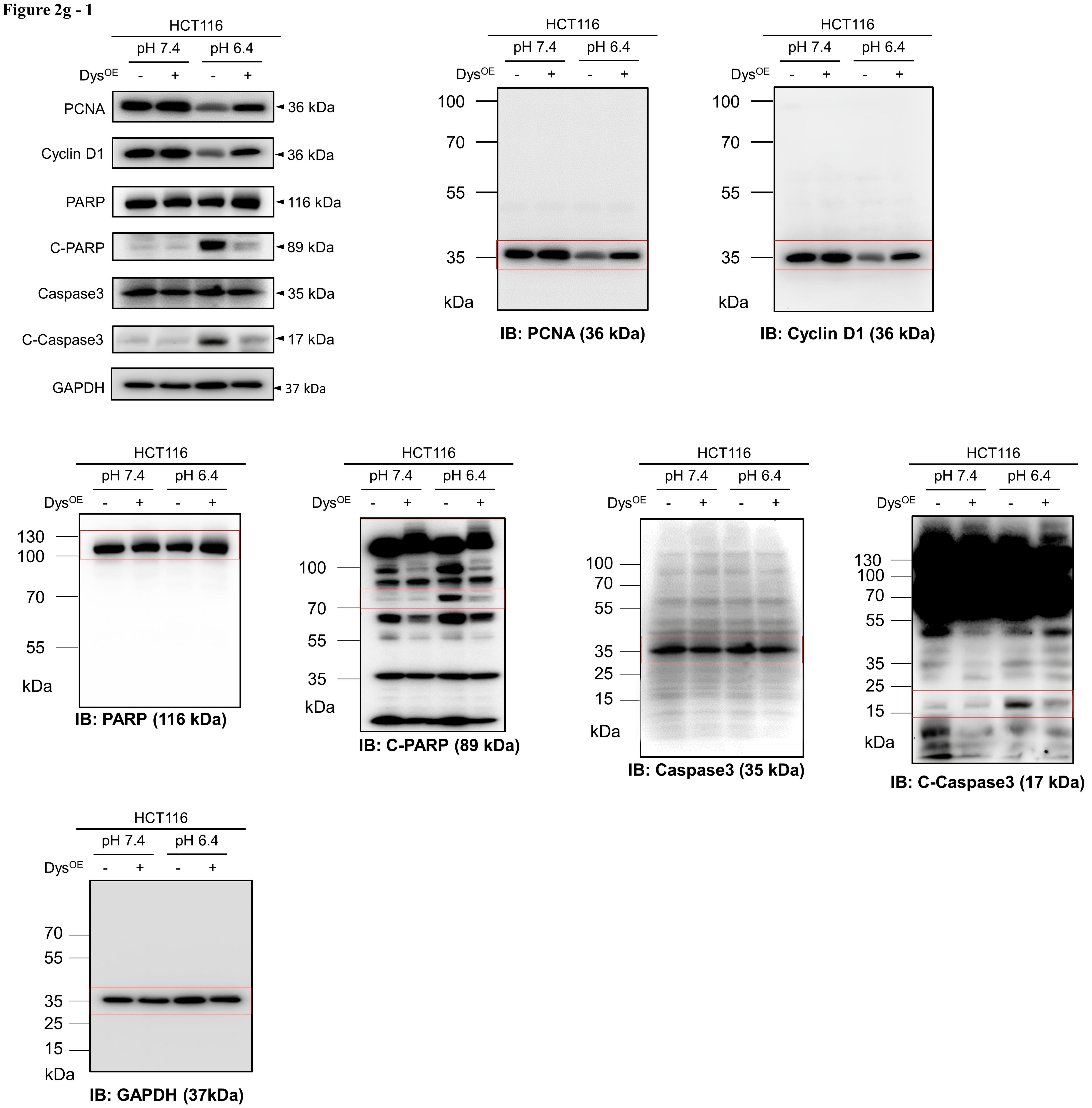
HCT116
pH 7.4
pH 6.4
Dys^{OE} - + - +
PCNA
36 kDa
Cyclin D1
36 kDa
PARP
116 kDa
C-PARP
89 kDa
Caspase3
35 kDa
C-Caspase3
17 kDa
GAPDH
37 kDa
100 70 55 35 kDa
IB: PCNA (36 kDa)
IB: Cyclin D1 (36 kDa)
130 100 70 55 kDa
IB: PARP (116 kDa)
100 70 55 35 kDa
IB: C-PARP (89 kDa)
100 70 55 35 25 15 kDa
IB: Caspase3 (35 kDa)
130 100 70 55 35 25 15 kDa
IB: C-Caspase3 (17 kDa)
70 55 35 25 15 kDa
IB: GAPDH (37kDa)

Figure 2g - 2

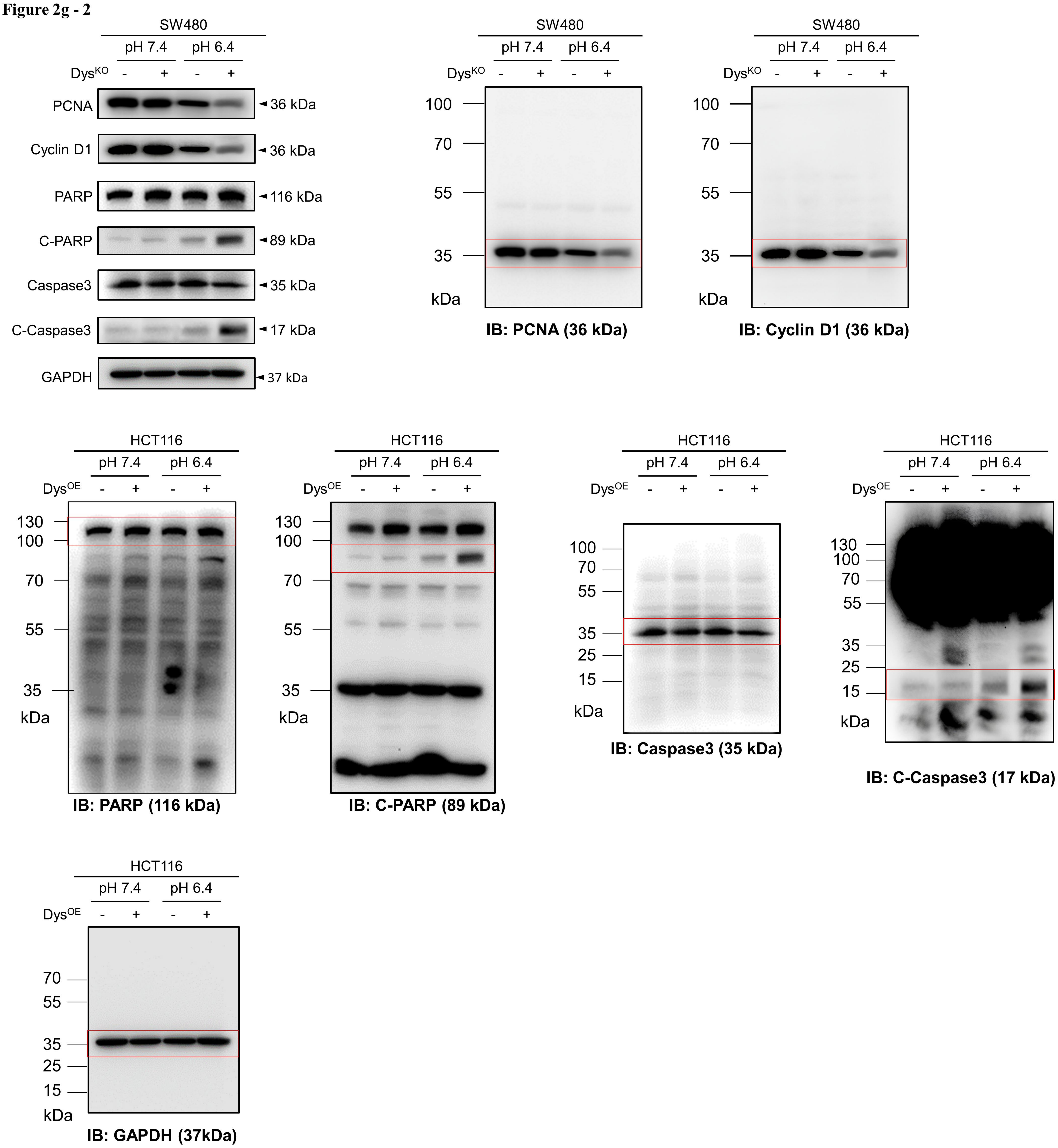
SW480
pH 7.4
pH 6.4
Dys^KO
- + - +
PCNA
36 kDa
Cyclin D1
36 kDa
PARP
116 kDa
C-PARP
89 kDa
Caspase3
35 kDa
C-Caspase3
17 kDa
GAPDH
37 kDa
100
70
55
35
kDa
IB: PCNA (36 kDa)
IB: Cyclin D1 (36 kDa)
HCT116
Dys^OE
130
25
15
IB: PARP (116 kDa)
IB: C-PARP (89 kDa)
IB: Caspase3 (35 kDa)
IB: C-Caspase3 (17 kDa)
IB: GAPDH (37kDa)

Supplementary Figure 2b - 1

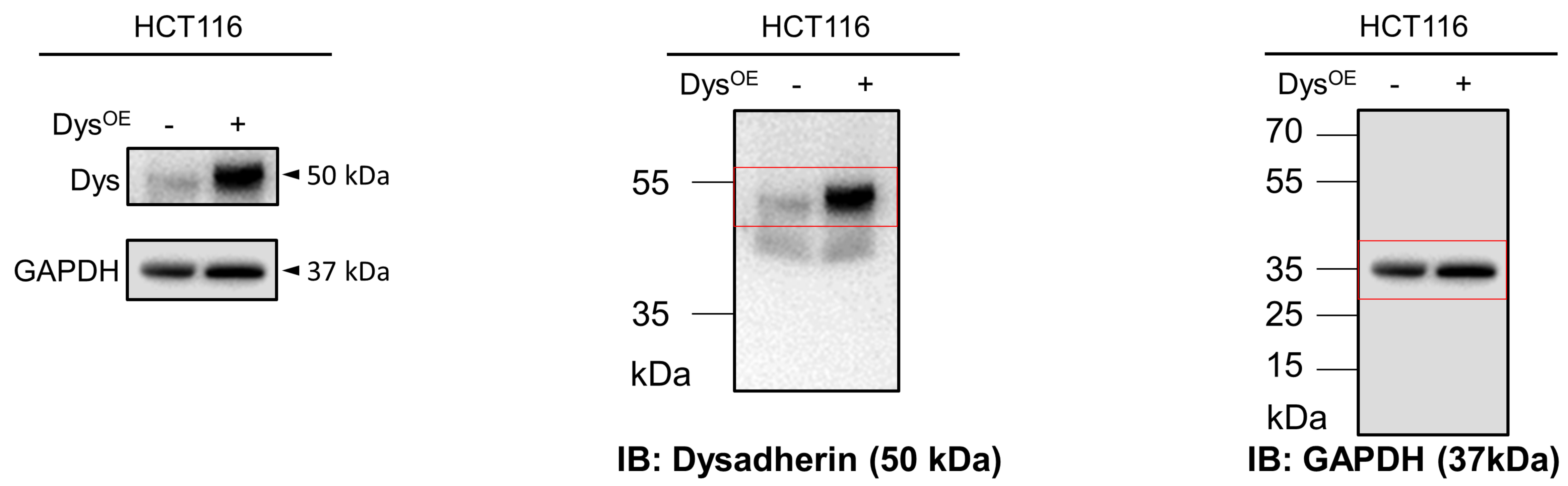

Supplementary Figure 2b - 2

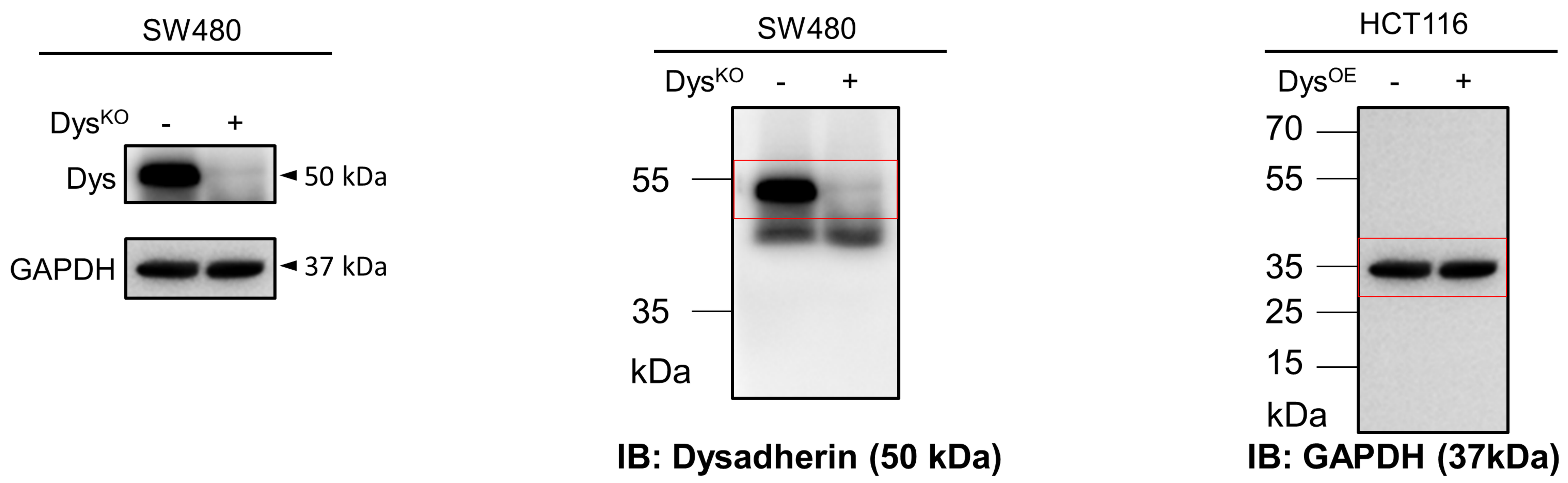

Figure 3e

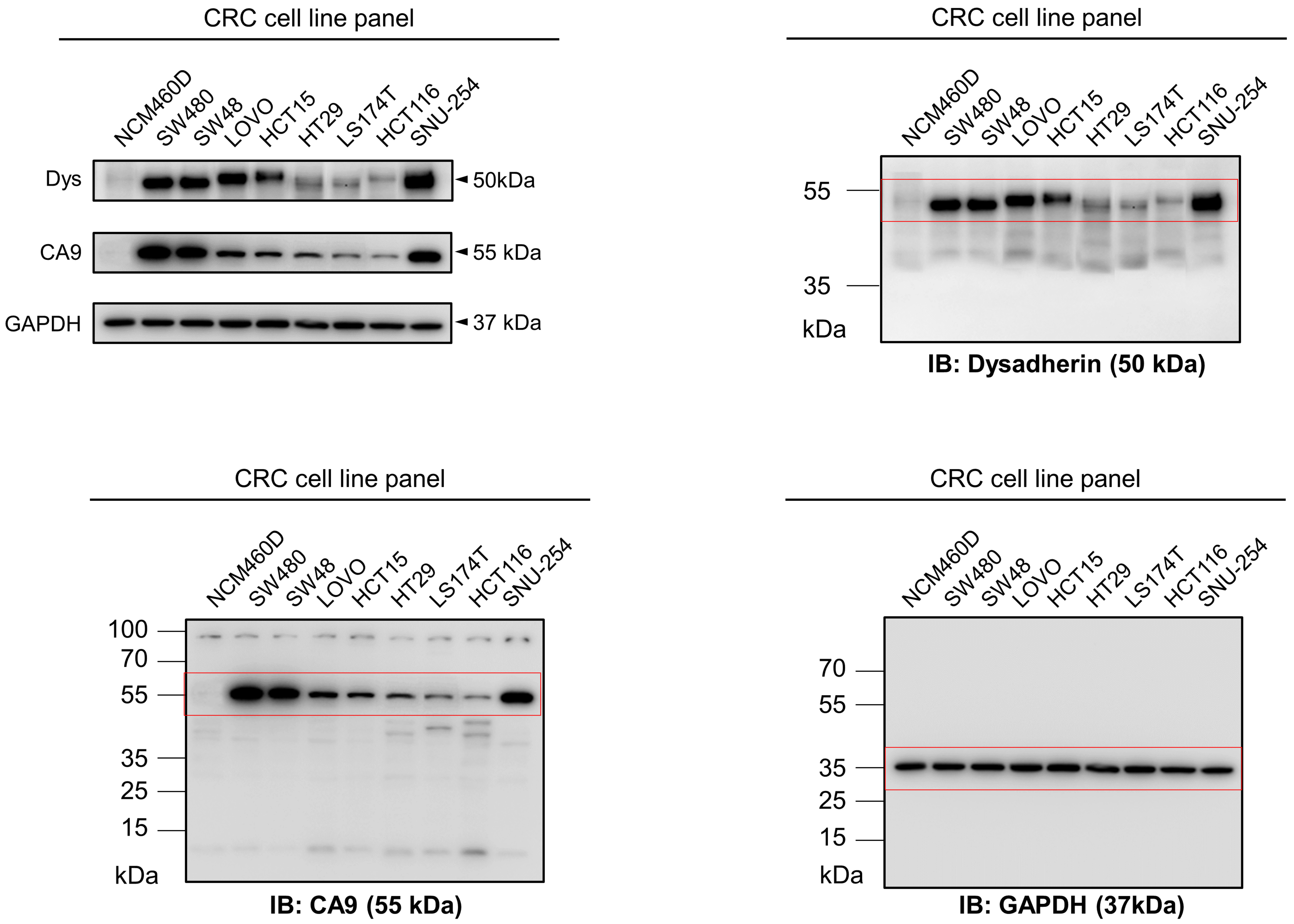
CRC cell line panel
NCM460D
SW480
SW48
LOVO
HCT15
HT29
LS174T
HCT116
SNU-254
Dys
◄ 50kDa
CA9
◄ 55 kDa
GAPDH
◄ 37 kDa
55
35
kDa
IB: Dysadherin (50 kDa)
100
70
55
35
25
15
kDa
IB: CA9 (55 kDa)
70
55
35
25
15
kDa
IB: GAPDH (37kDa)

Figure 4c

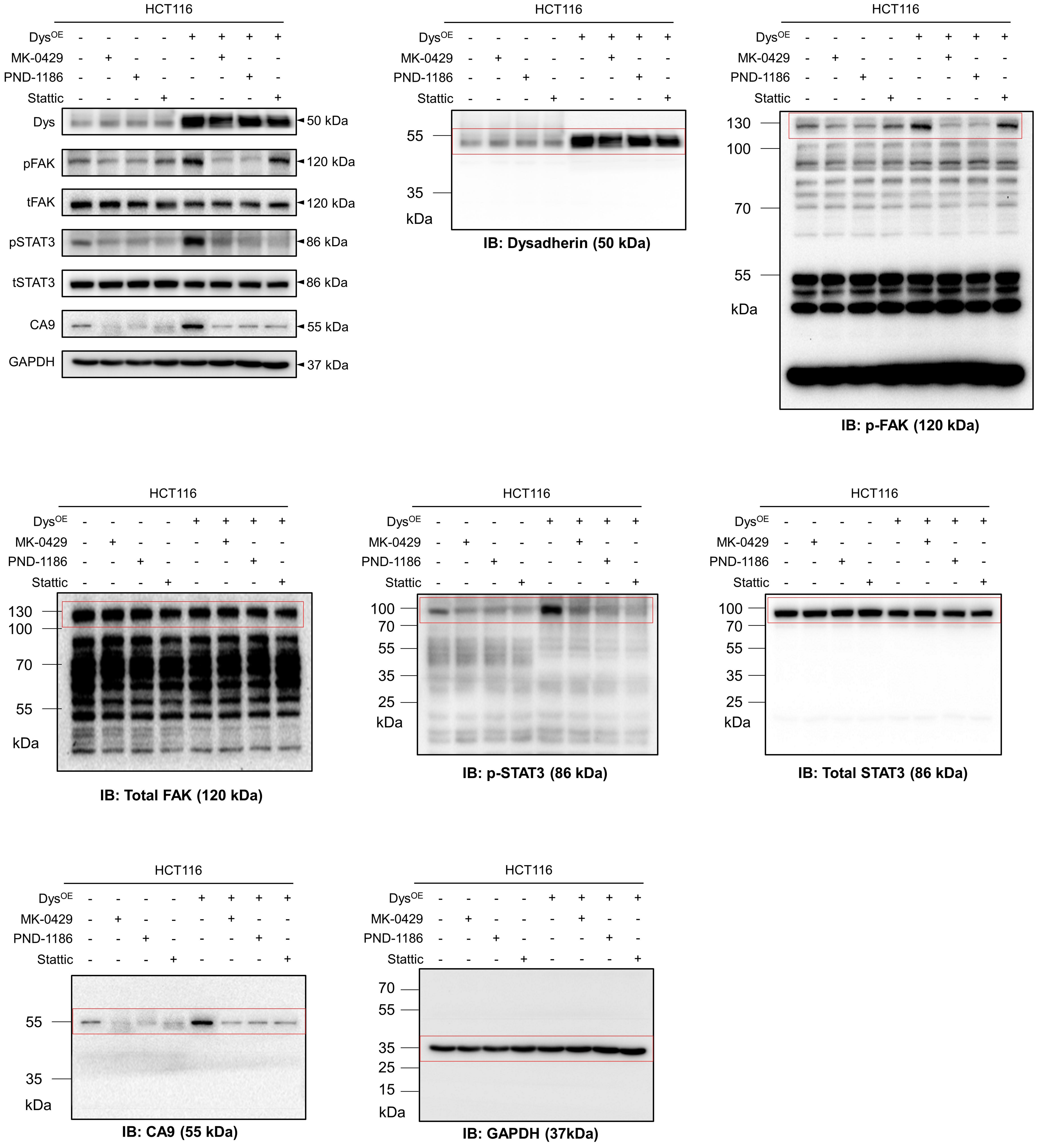
HCT116
DysOE
MK-0429
PND-1186
Stattic
Dys
pFAK
tFAK
pSTAT3
tSTAT3
CA9
GAPDH
50 kDa
120 kDa
120 kDa
86 kDa
86 kDa
55 kDa
37 kDa
IB: Dysadherin (50 kDa)
IB: p-FAK (120 kDa)
IB: Total FAK (120 kDa)
IB: p-STAT3 (86 kDa)
IB: Total STAT3 (86 kDa)
IB: CA9 (55 kDa)
IB: GAPDH (37kDa)
kDa

Supplementary Figure 4a - 1

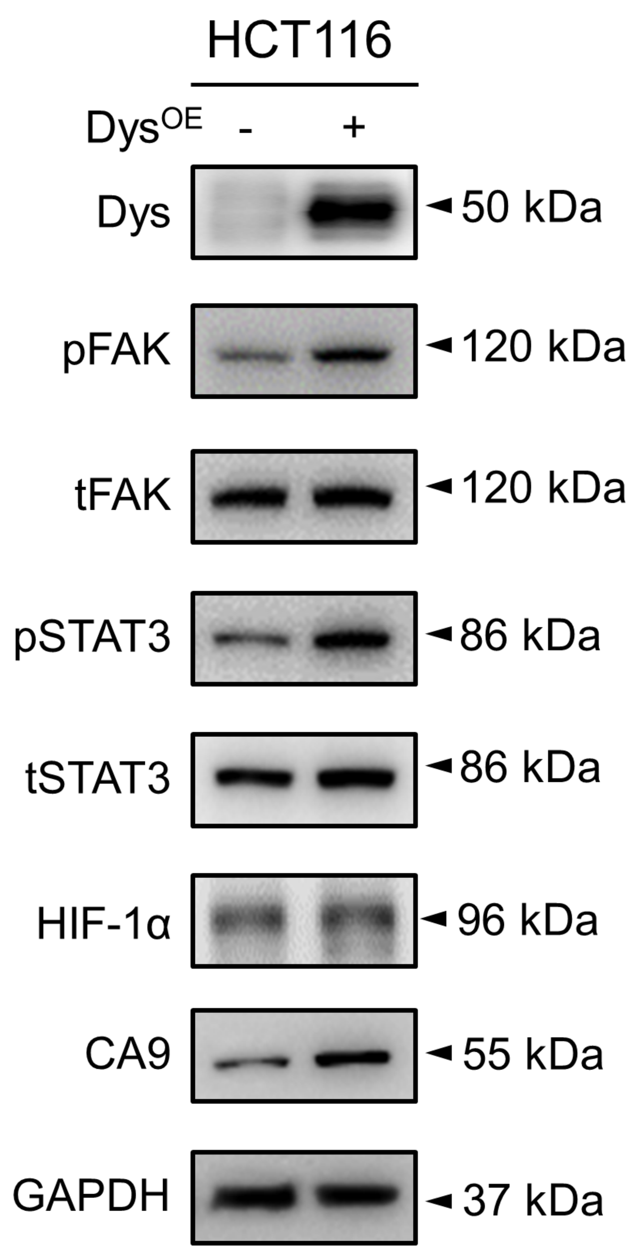

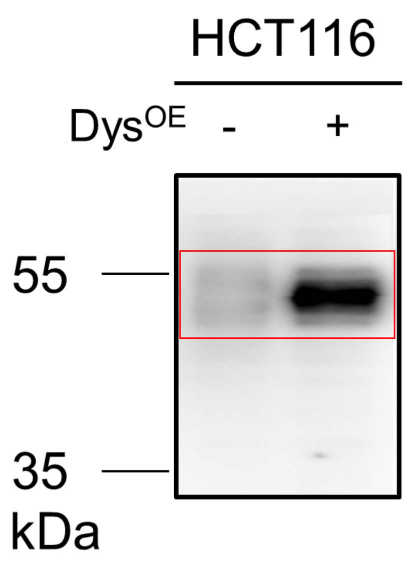

IB: Dysadherin (50 kDa)

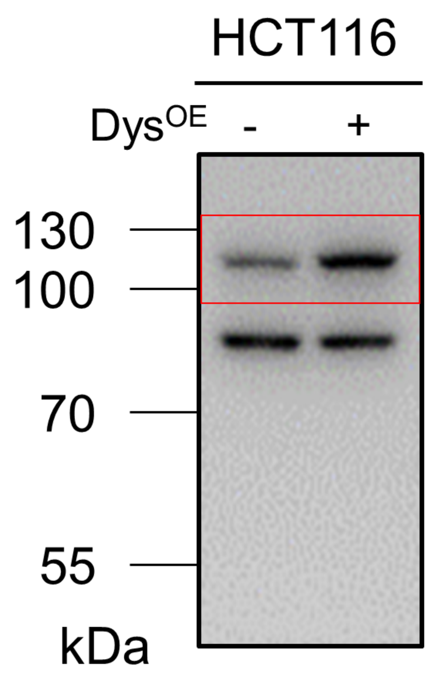

IB: p-FAK (120 kDa)

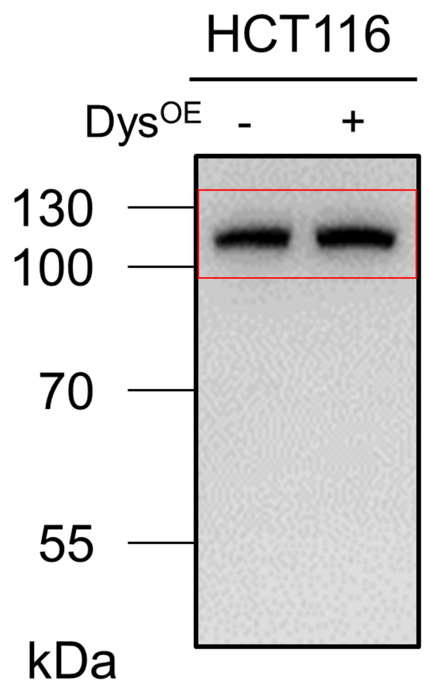

IB: Total FAK (120 kDa)

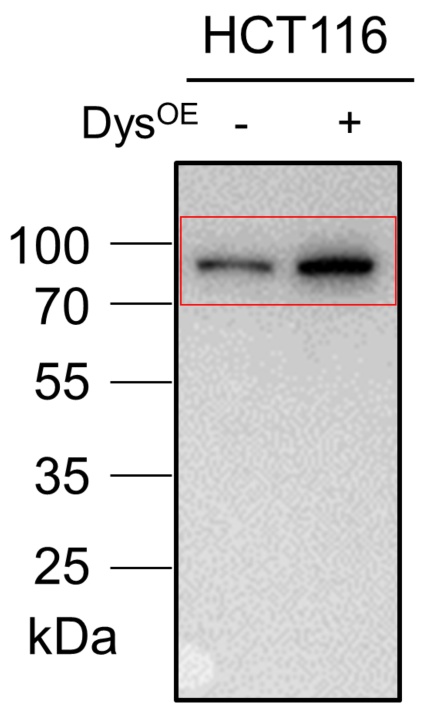

IB: p-STAT3 (86 kDa)

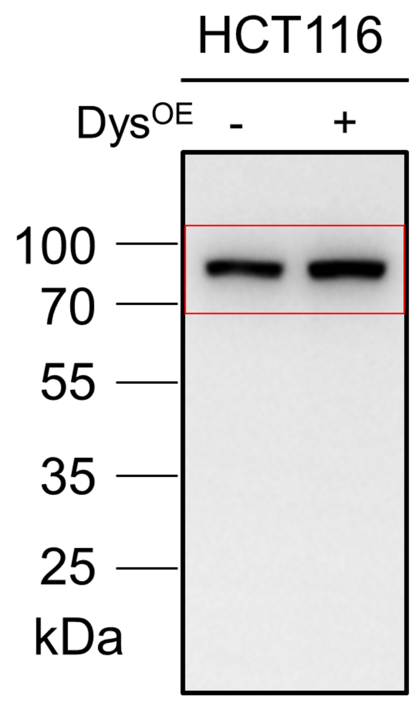

IB: Total STAT3 (86 kDa)

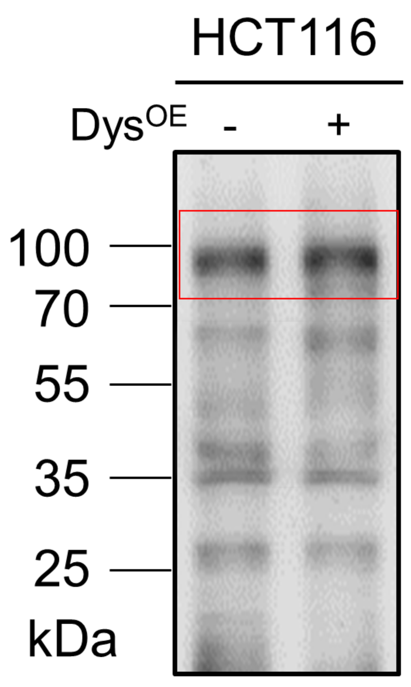

IB: HIF-1α (96 kDa)

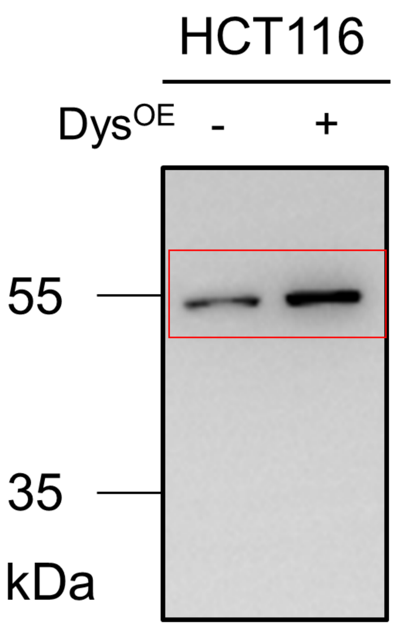

IB: CA9 (55 kDa)

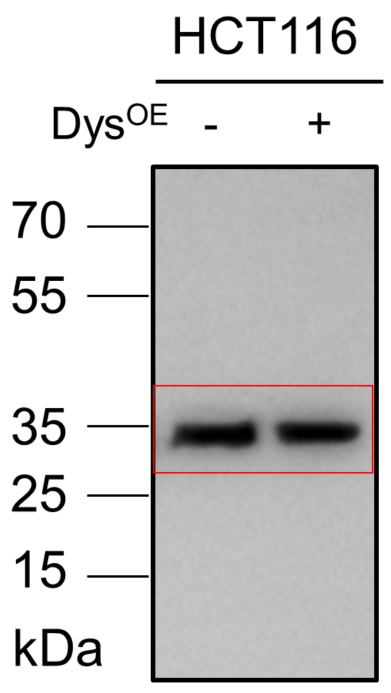

IB: GAPDH (37kDa)

Supplementary Figure 4a - 2

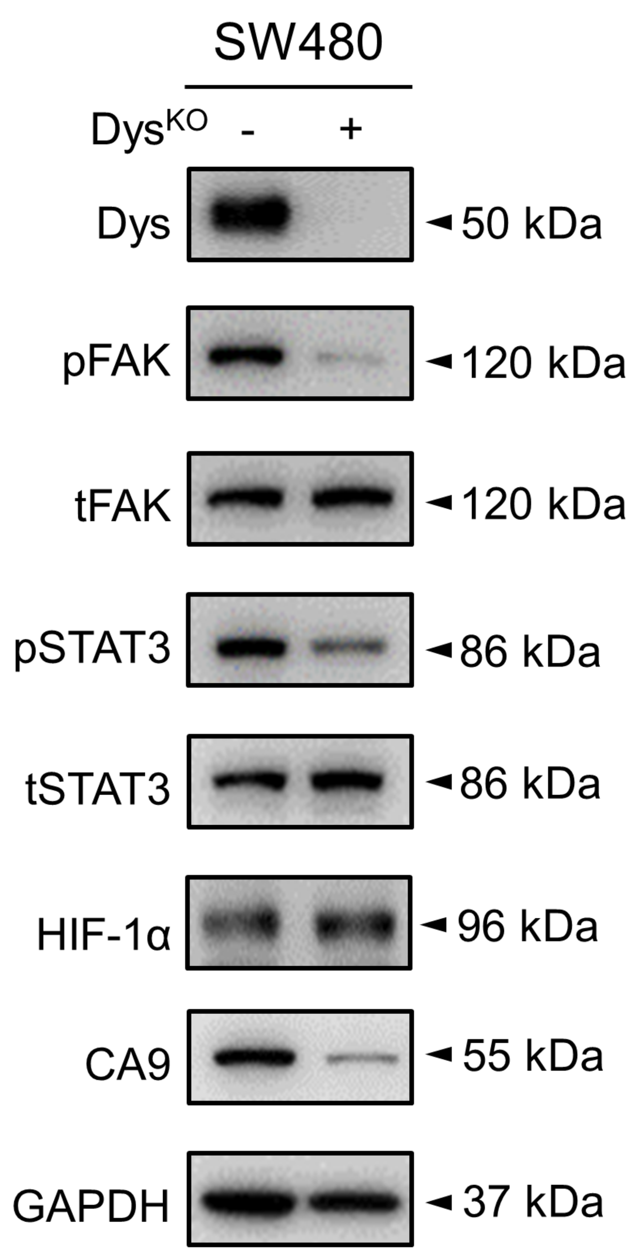

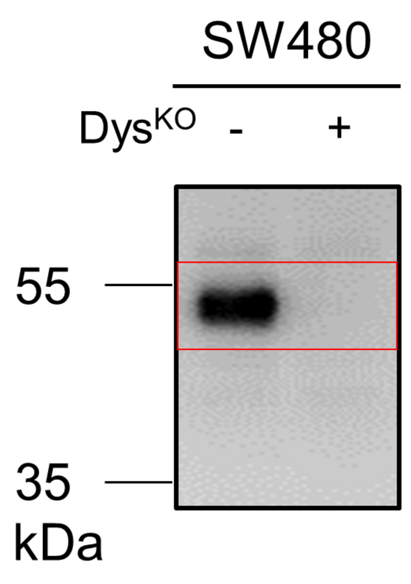

IB: Dysadherin (50 kDa)

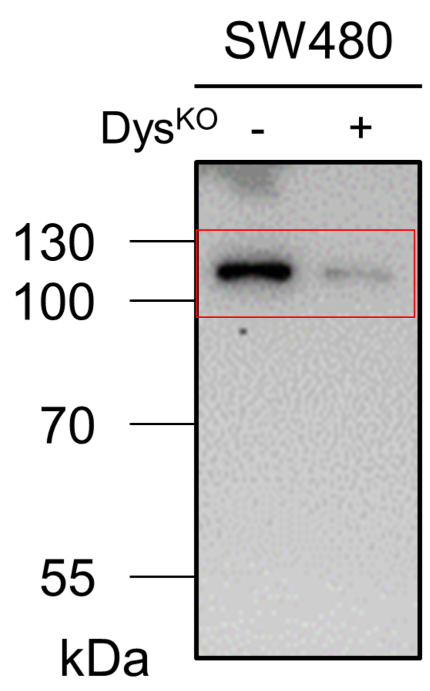

IB: p-FAK (120 kDa)

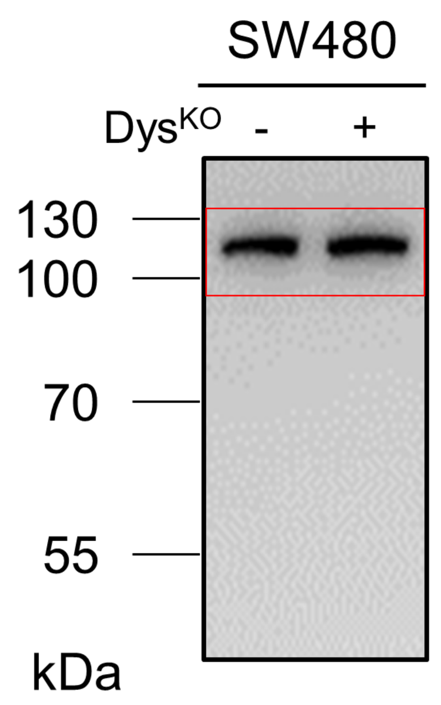

IB: Total FAK (120 kDa)

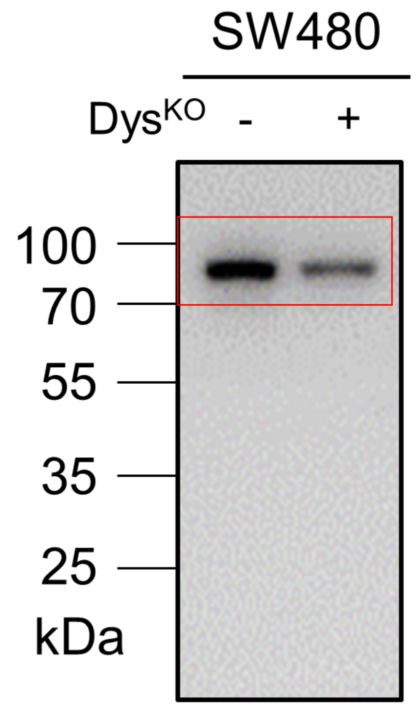

IB: p-STAT3 (86 kDa)

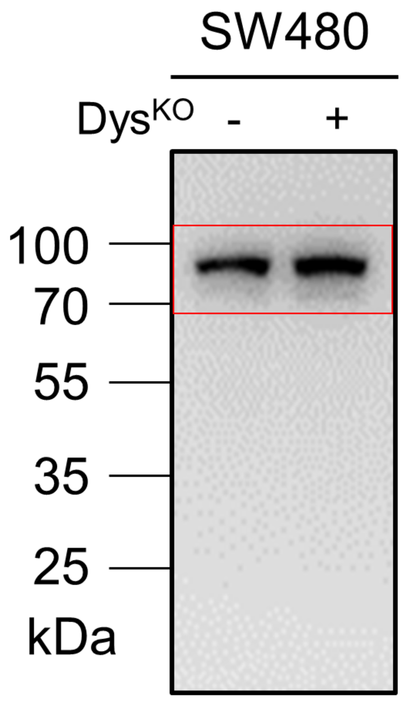

IB: Total STAT3 (86 kDa)

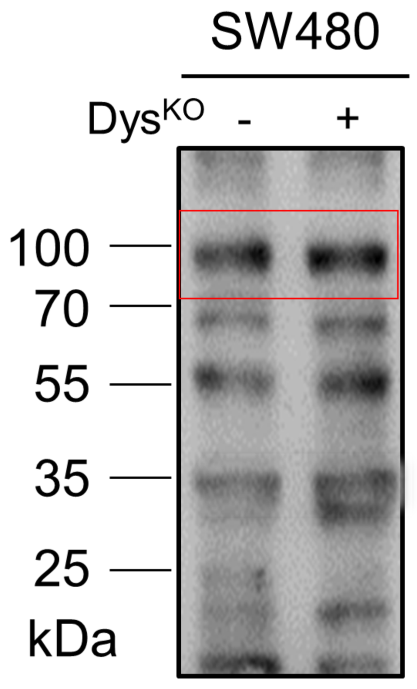

IB: HIF-1α (96 kDa)

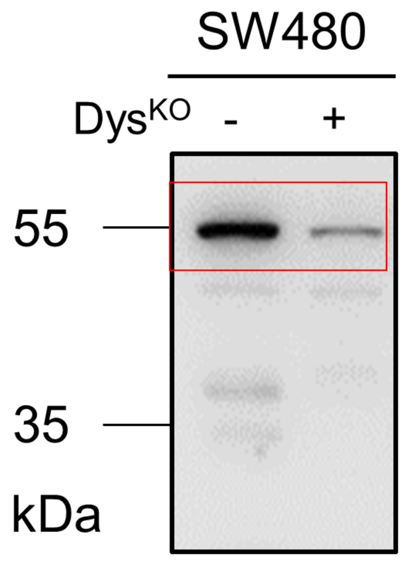

IB: CA9 (55 kDa)

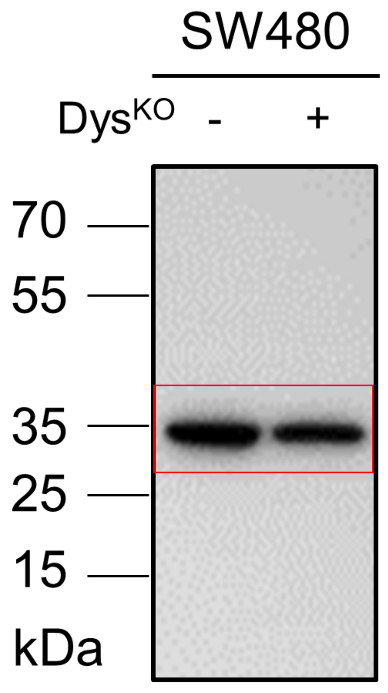

IB: GAPDH (37kDa)

Supplementary Figure 4a - 3

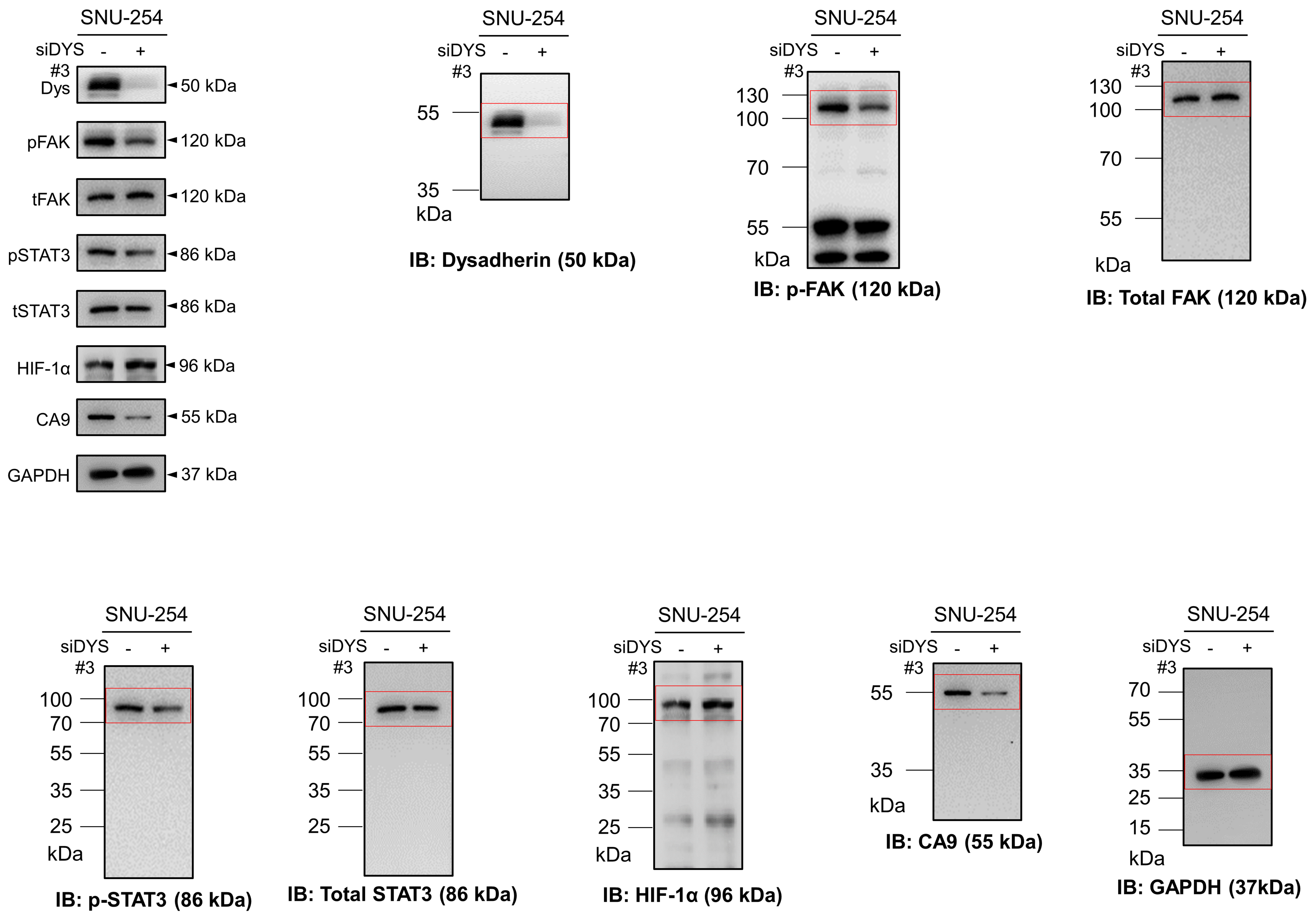

Supplementary Figure 4f

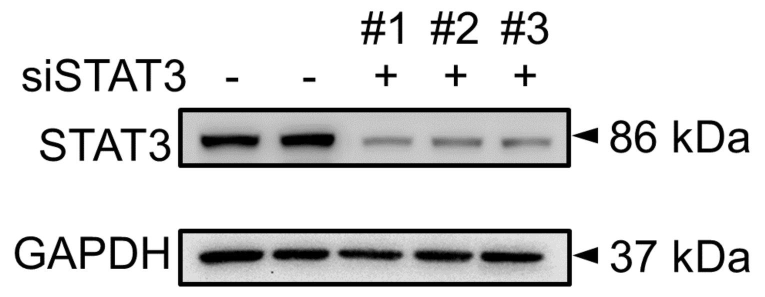

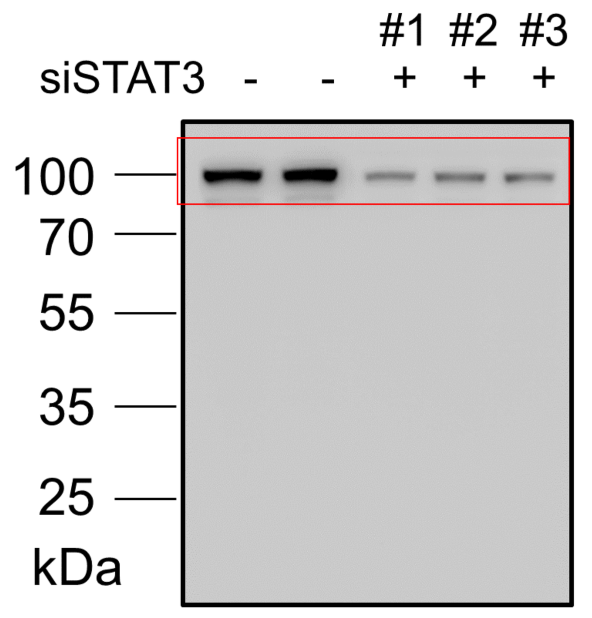

IB: Total STAT3 (86 kDa)

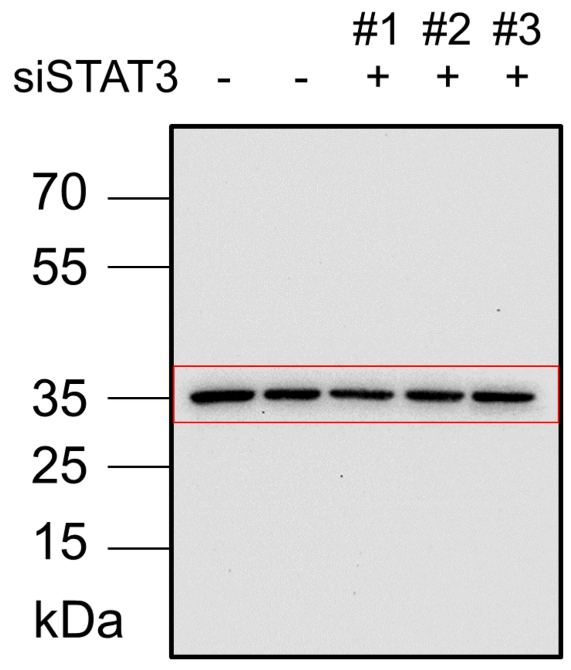

IB: GAPDH (37kDa)

Supplementary Figure 4e - 1

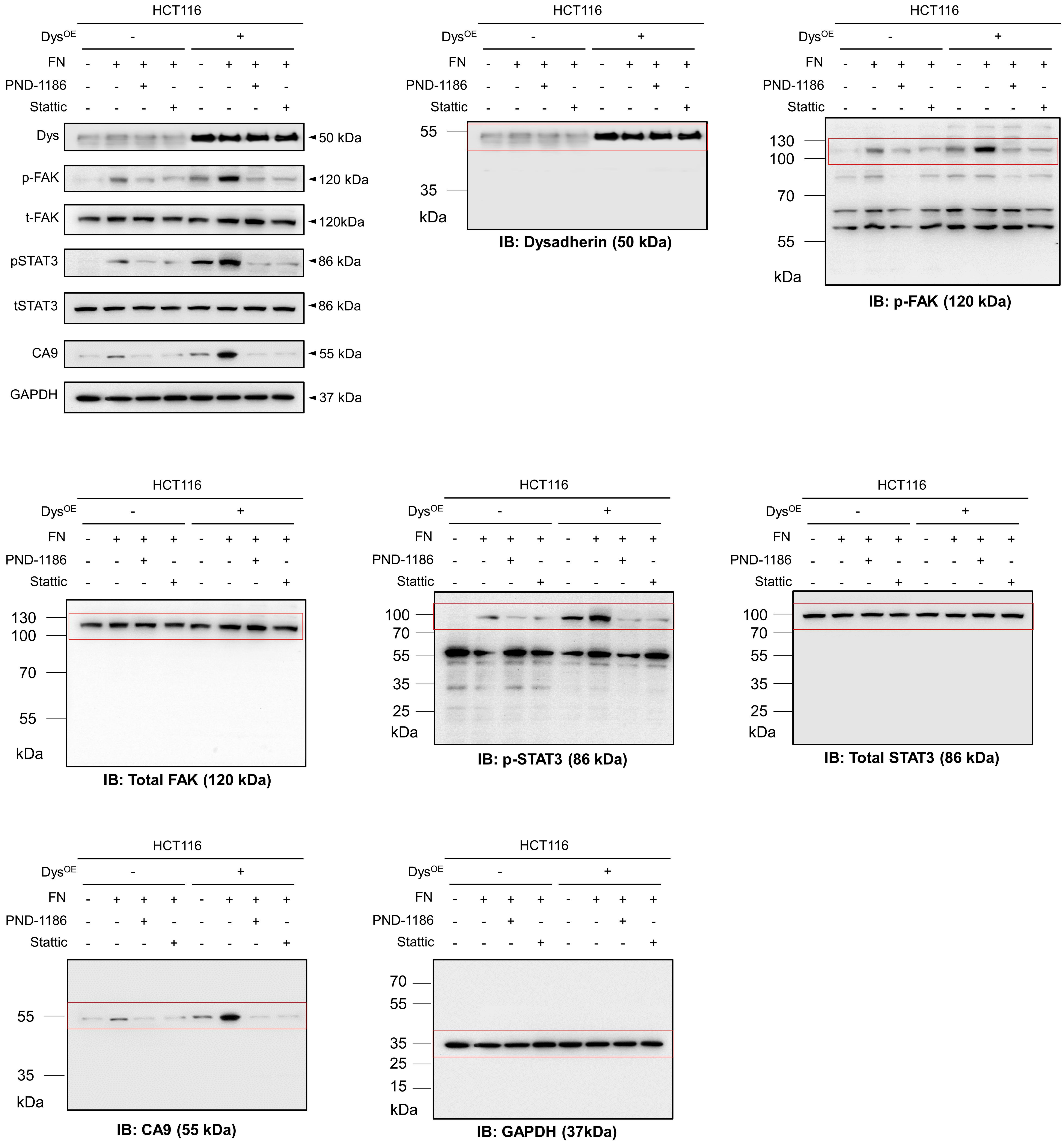

Supplementary Figure 4e - 2

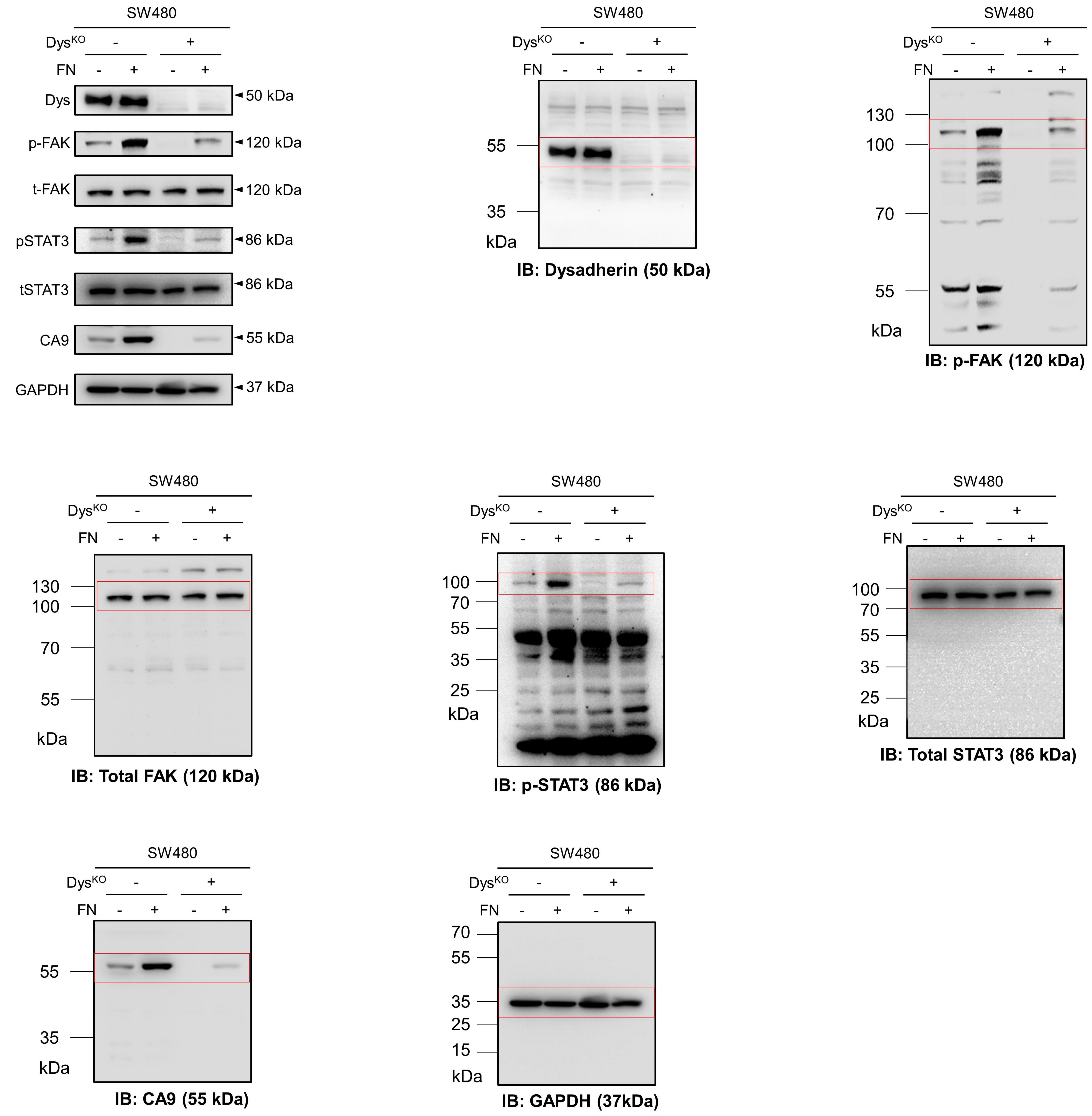

Supplementary Figure 4f

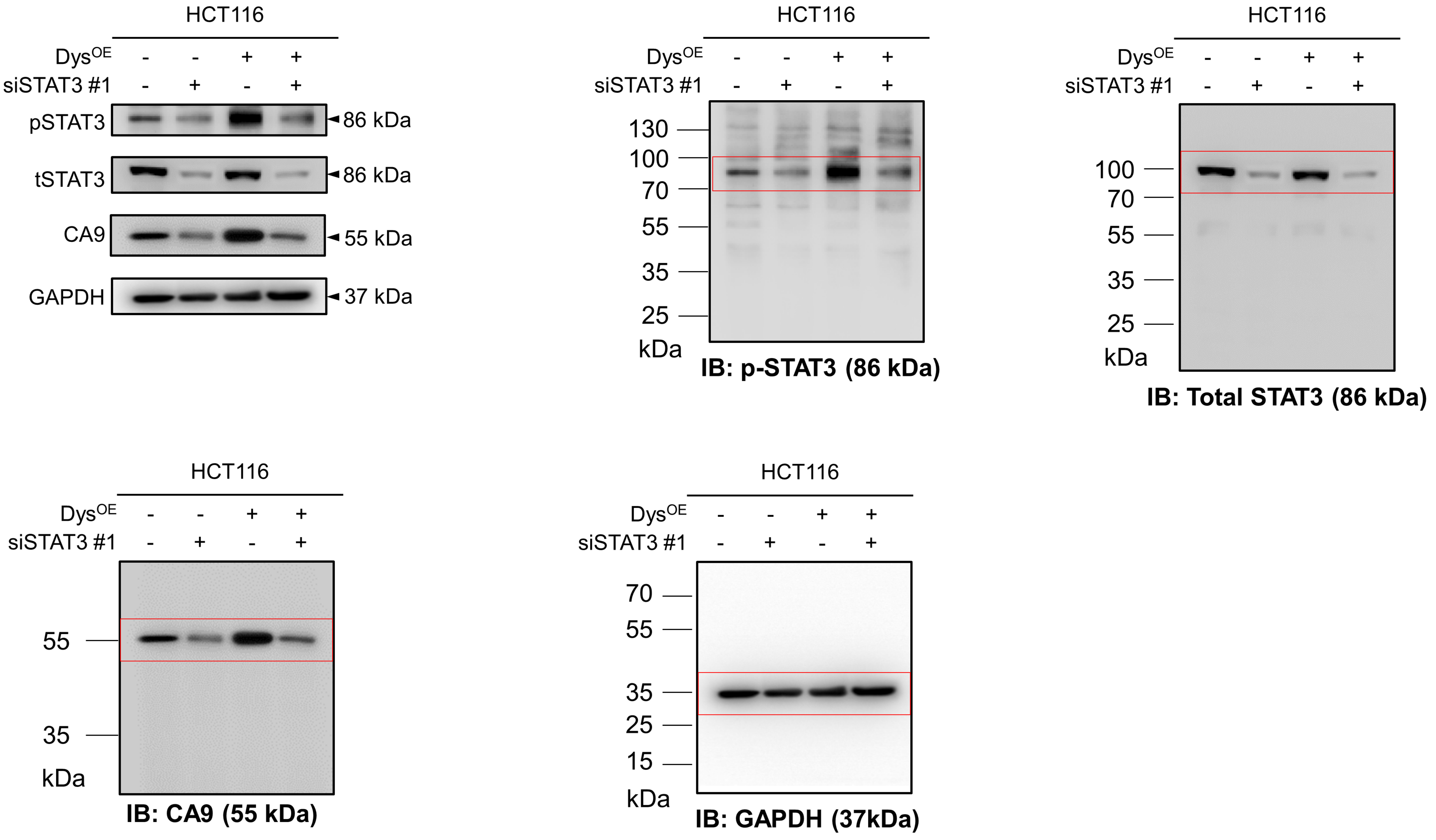

Supplementary Figure 4i

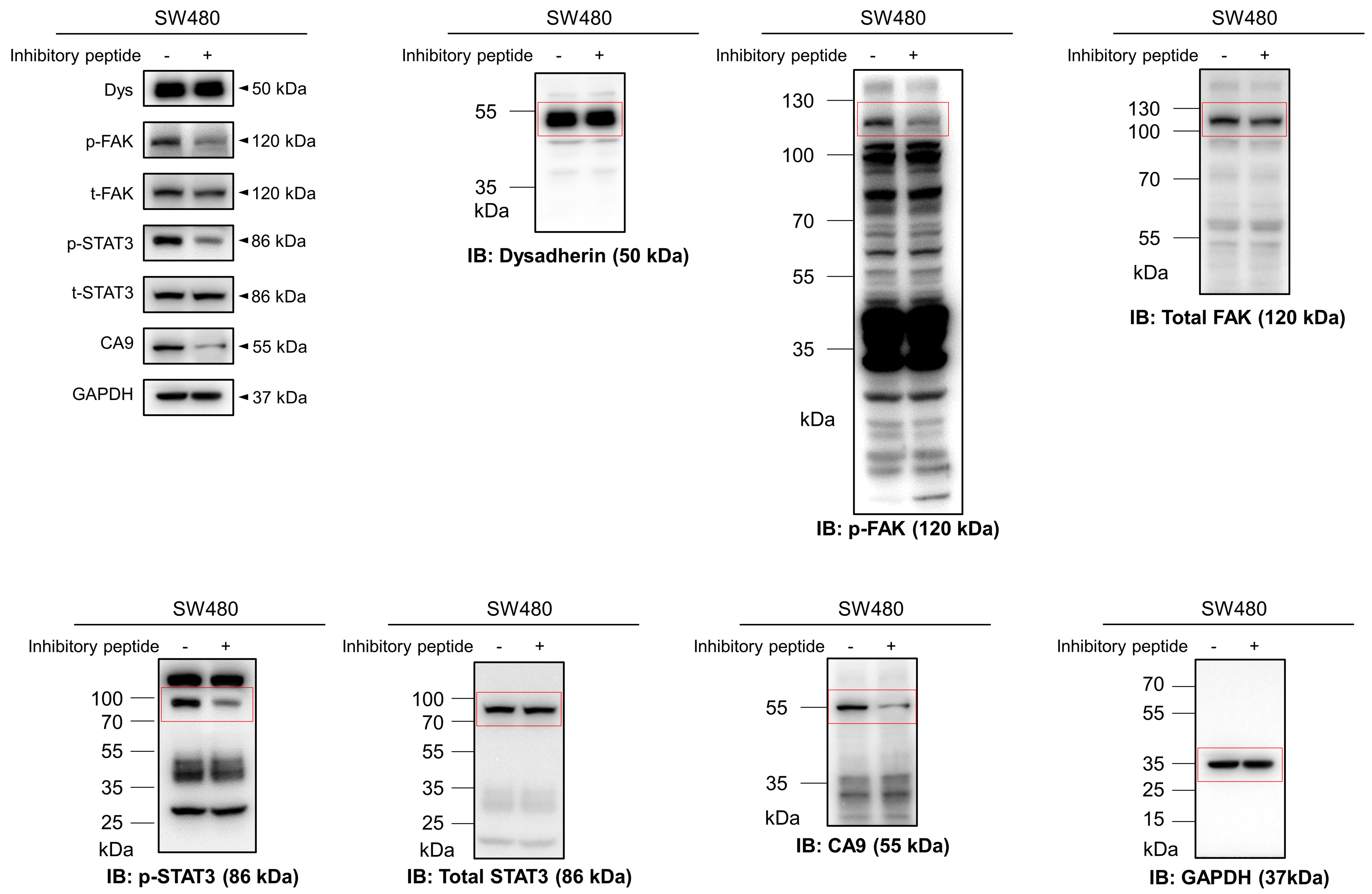

Supplementary Figure 4n

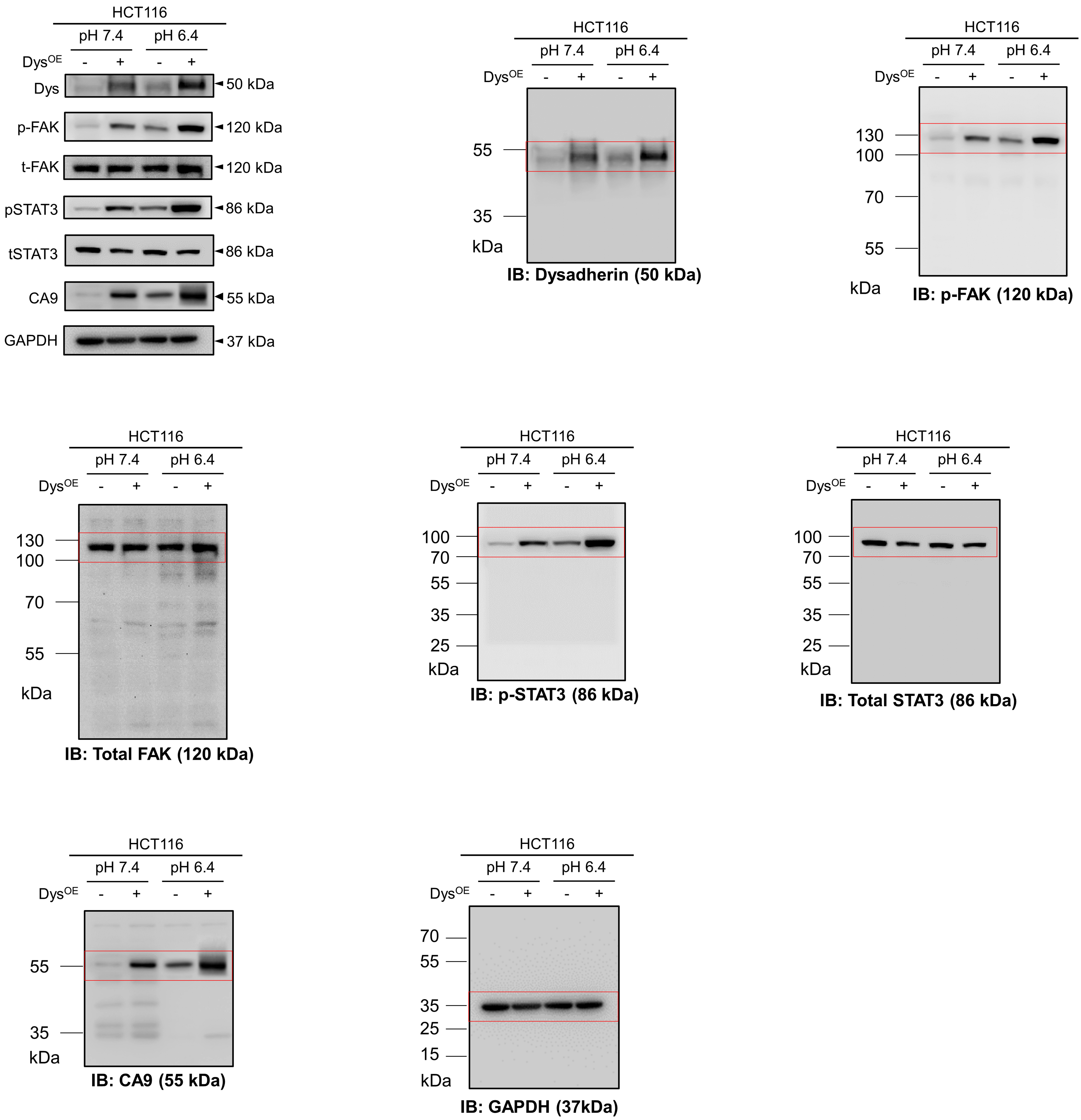

Figure 5e

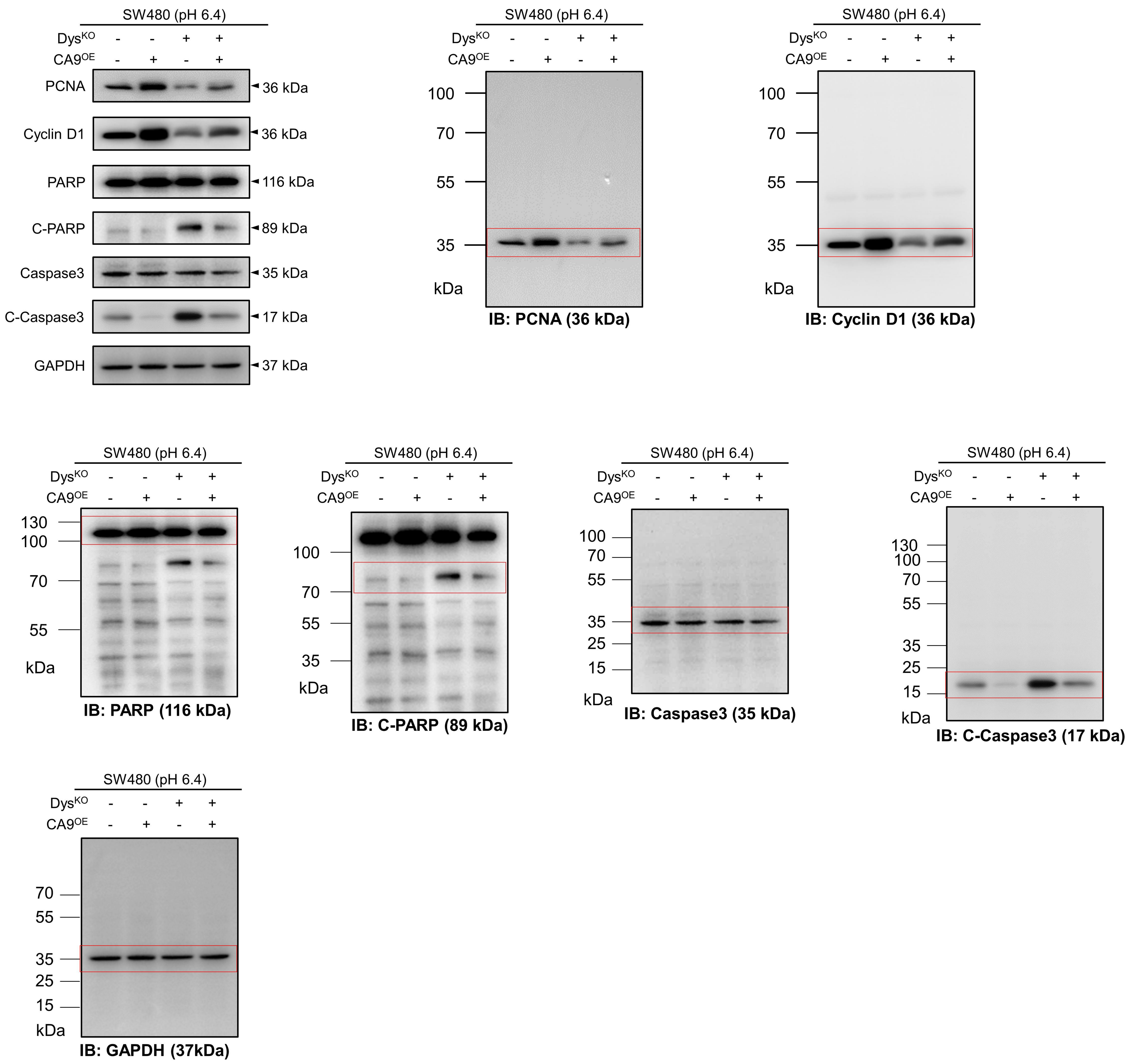
SW480 (pH 6.4)
Dys^KO - - + +
CA9^OE - + - +
PCNA
36 kDa
Cyclin D1
36 kDa
PARP
116 kDa
C-PARP
89 kDa
Caspase3
35 kDa
C-Caspase3
17 kDa
GAPDH
37 kDa
100
70
55
35
kDa
IB: PCNA (36 kDa)
IB: Cyclin D1 (36 kDa)
130
15
25
IB: PARP (116 kDa)
IB: C-PARP (89 kDa)
IB: Caspase3 (35 kDa)
IB: C-Caspase3 (17 kDa)
IB: GAPDH (37kDa)

Supplementary Figure 5a

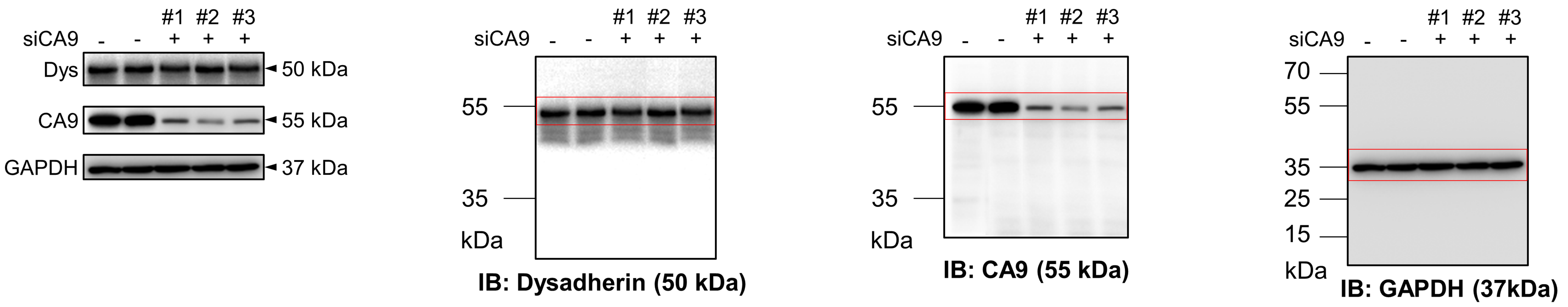

Supplementary Figure 5c

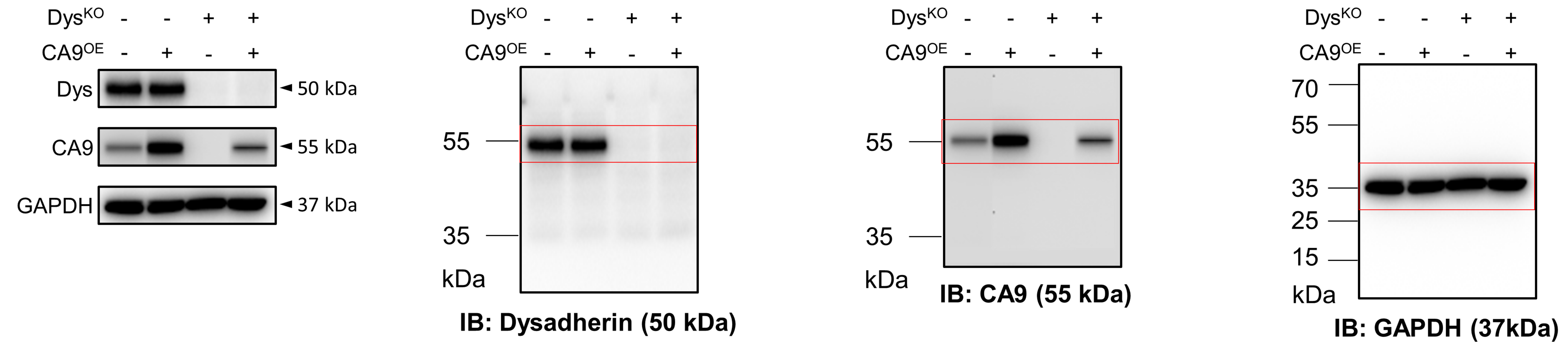

Supplementary Figure 5l

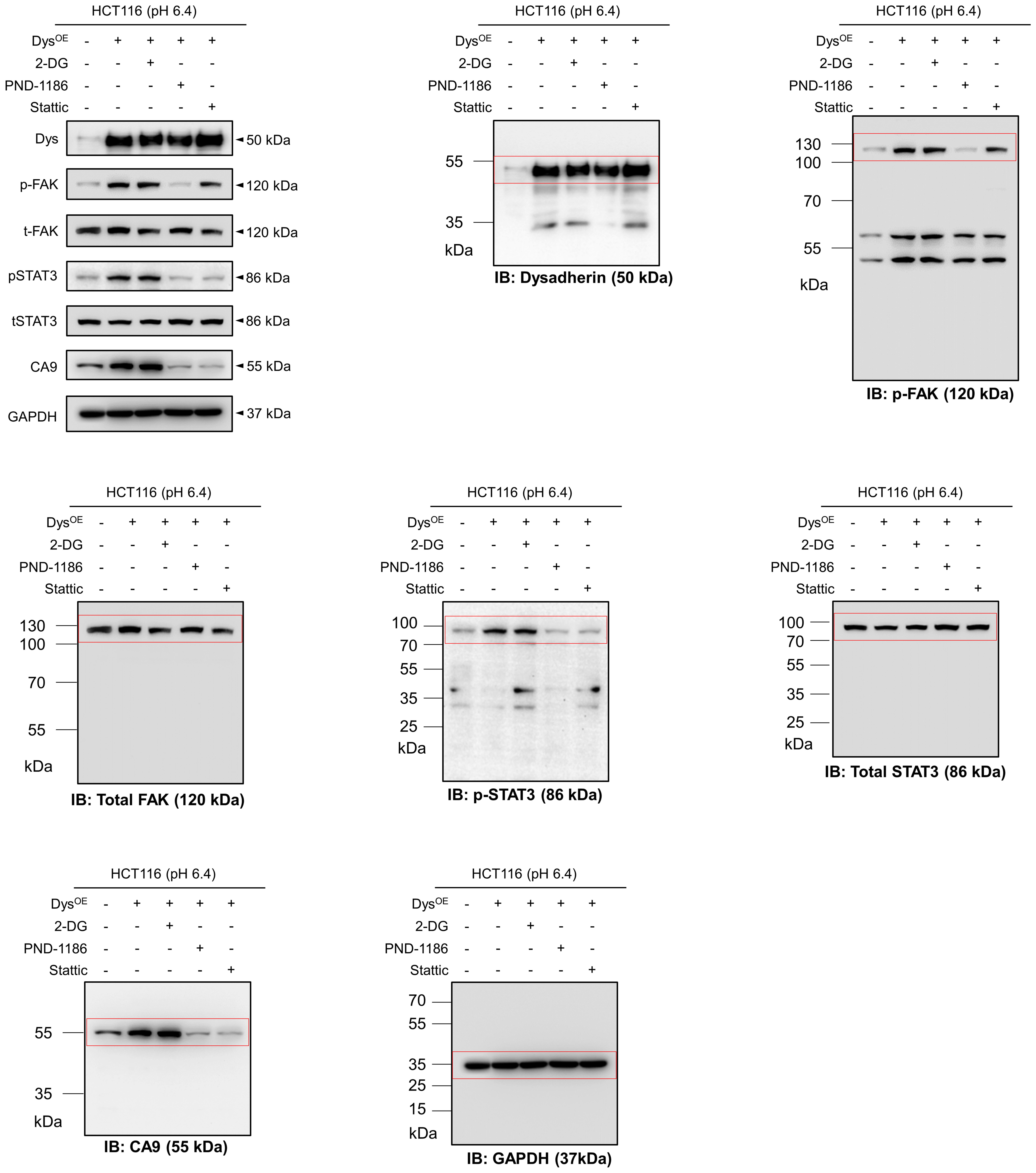

Fig 2b

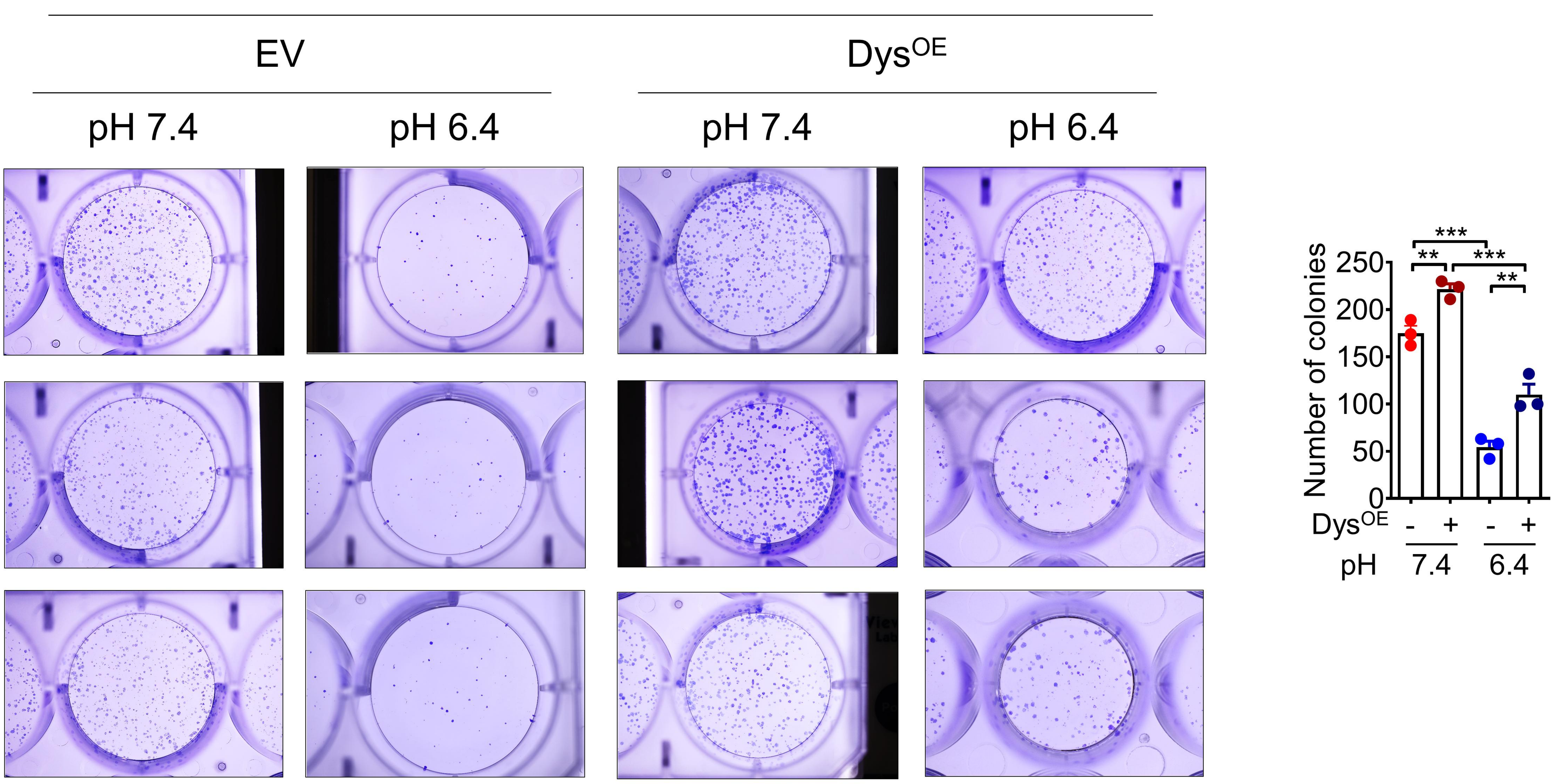
HCT116
EV
Dys^OE
pH 7.4
pH 6.4
pH 7.4
pH 6.4
Number of colonies
250
200
150
100
50
0
***
**
***
**
Dys^OE
- + - +
pH
7.4
6.4

Fig 2c

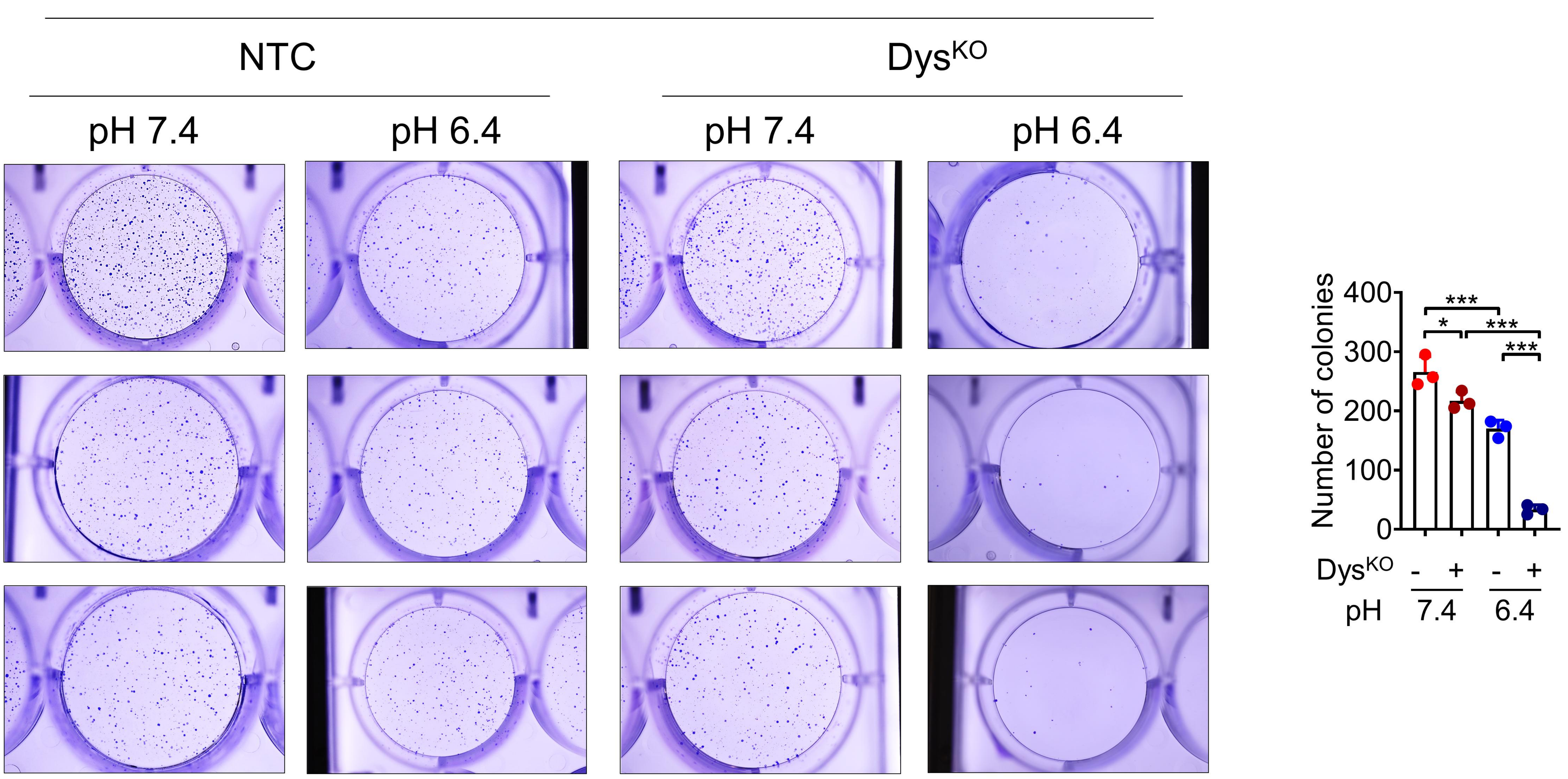
SW480
NTC
Dys^KO
pH 7.4
pH 6.4
pH 7.4
pH 6.4
Number of colonies
400
300
200
100
0
***
*
***
***
Dys^KO
- + - +
pH
7.4
6.4

Sup 3i

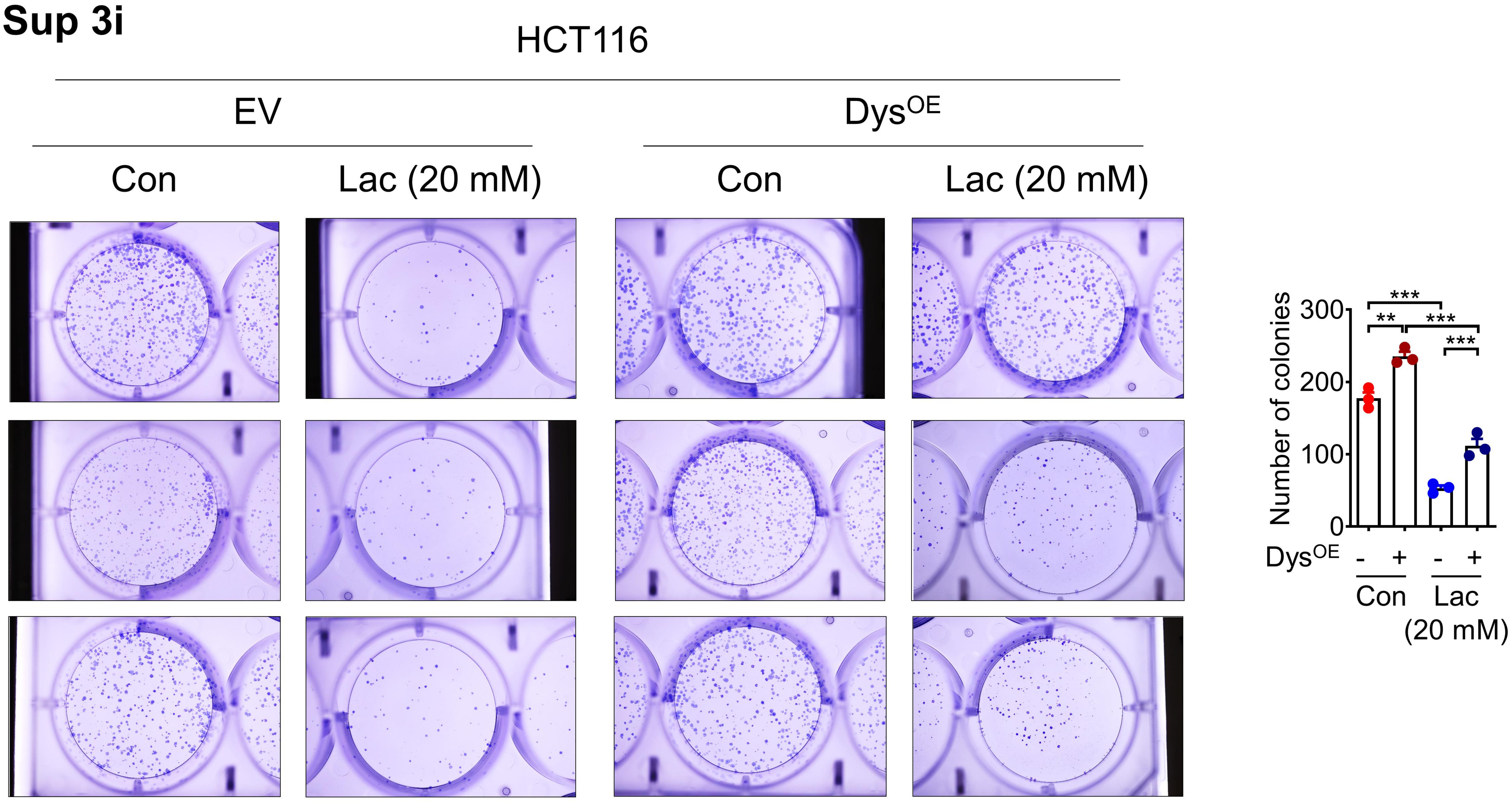
HCT116
EV
DysOE
Con
Lac (20 mM)
Con
Lac (20 mM)
***
**
***
***
300
200
100
0
Number of colonies
DysOE
- + - +
Con
Lac
(20 mM)

Sup 3j

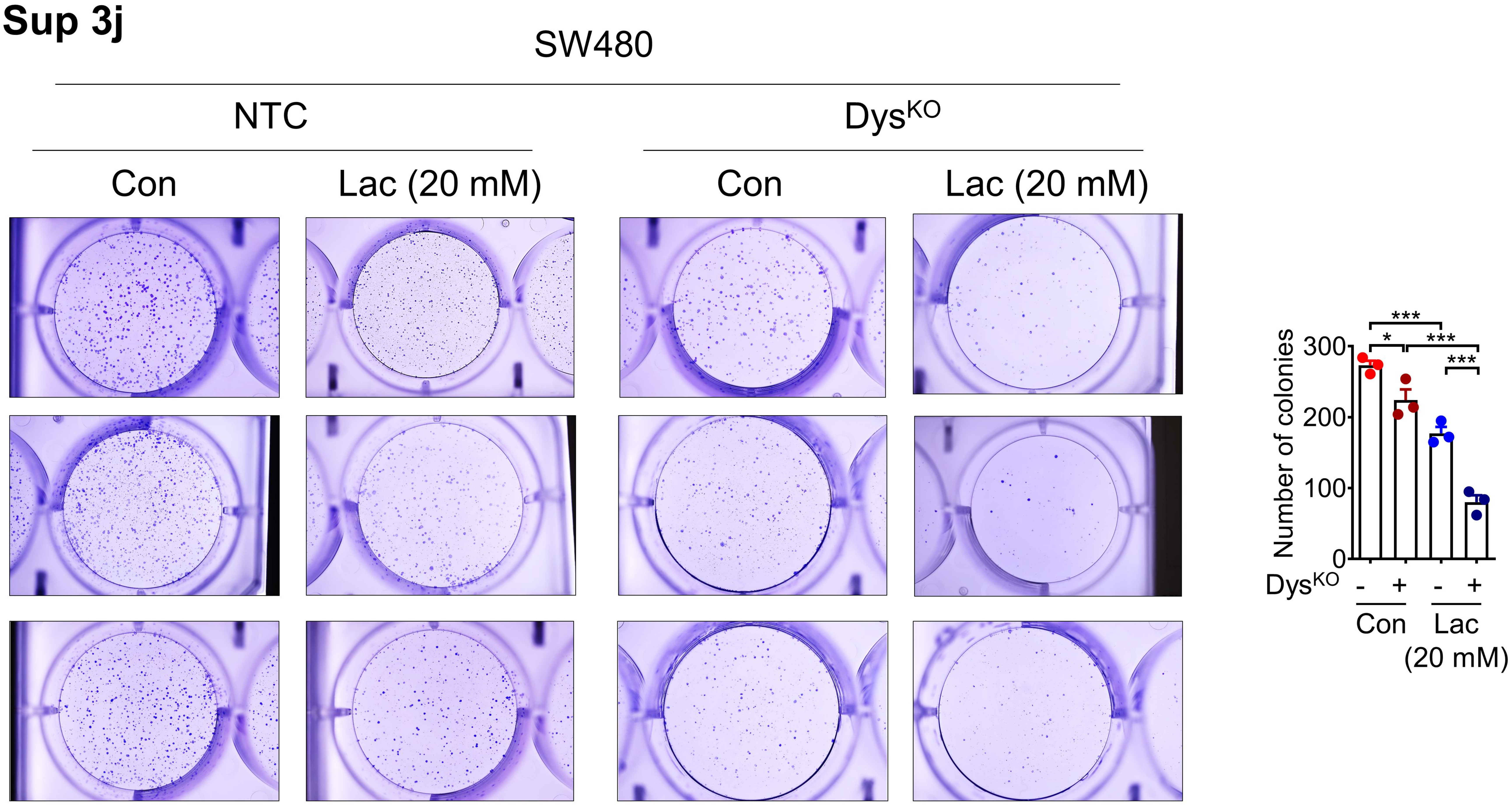
SW480
NTC
DysKO
Con
Lac (20 mM)
Con
Lac (20 mM)
***
*
***
***
300
200
100
0
Number of colonies
DysKO
- + - +
Con
Lac
(20 mM)

Fig 5a

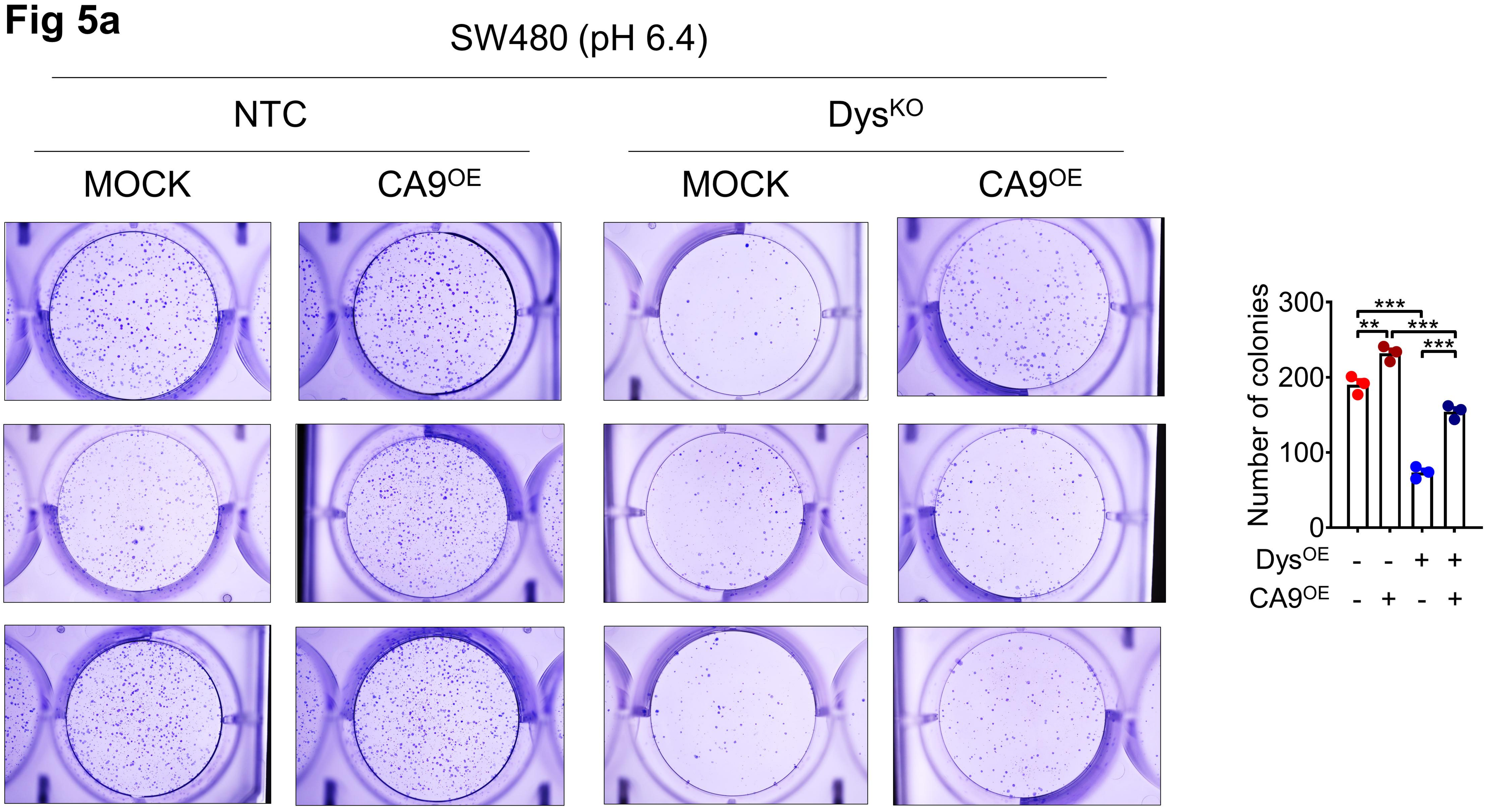

SW480 (pH 6.4)
NTC
DysKO
MOCK
CA9OE
MOCK
CA9OE
Number of colonies
300
200
100
0
***
**
***
***
DysOE - - + +
CA9OE - + - +

Fig 5g

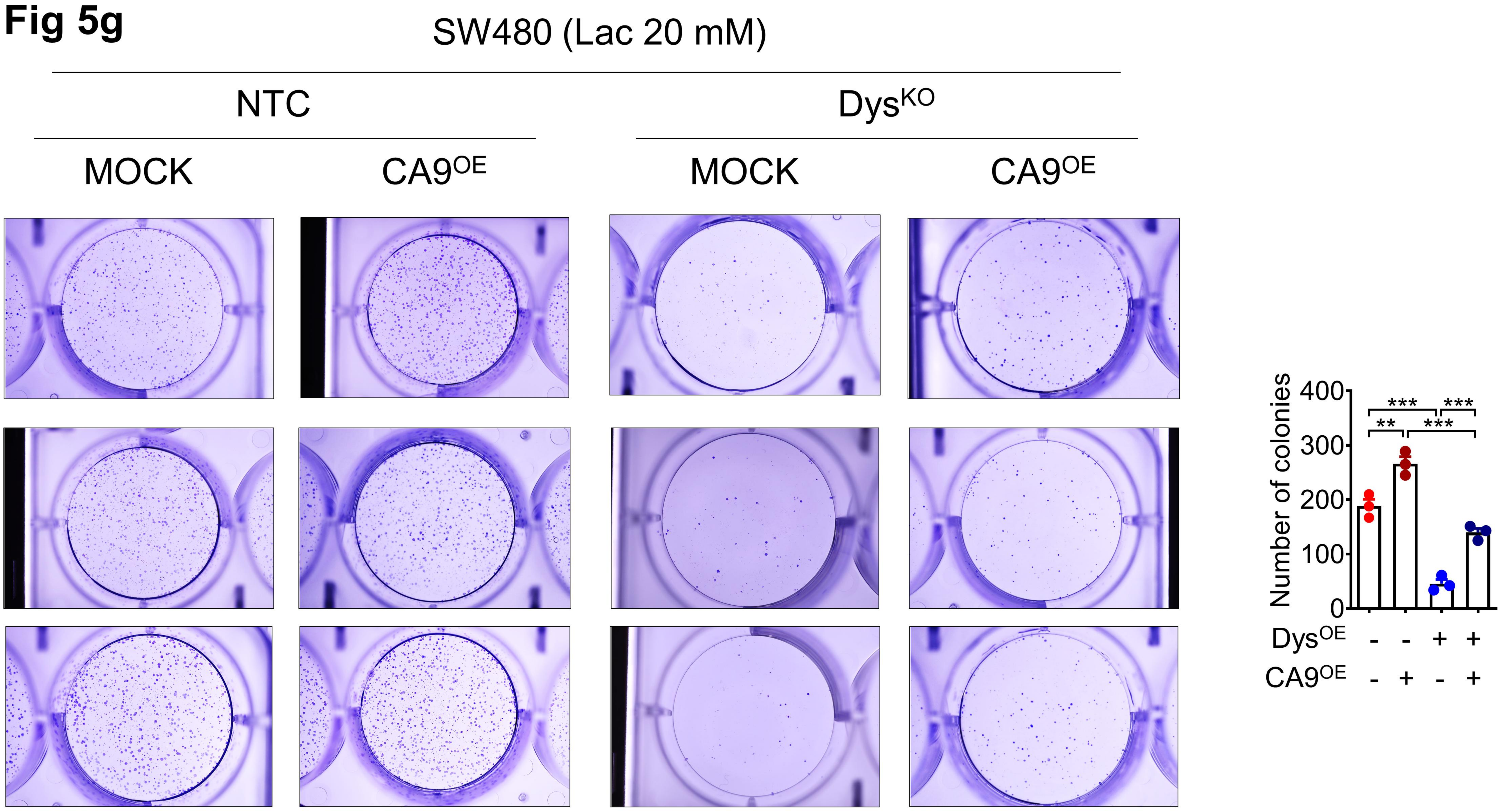

SW480 (Lac 20 mM)
NTC
DysKO
MOCK
CA9OE
MOCK
CA9OE
Number of colonies
400
300
200
100
0
***
***
**
***
DysOE - - + +
CA9OE - + - +

Fig 8a – Wound-healing

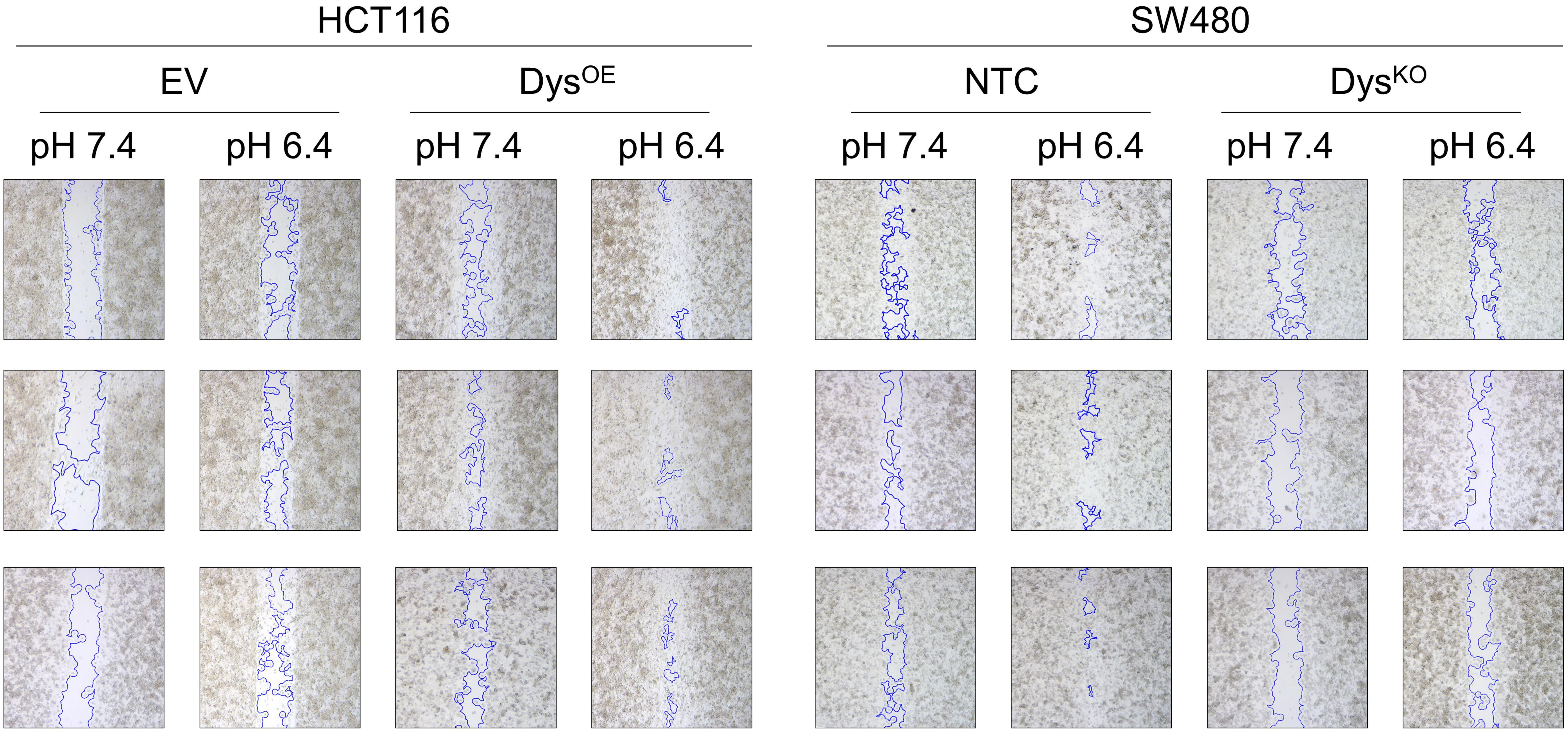

Fig. 8b – Wound-healing

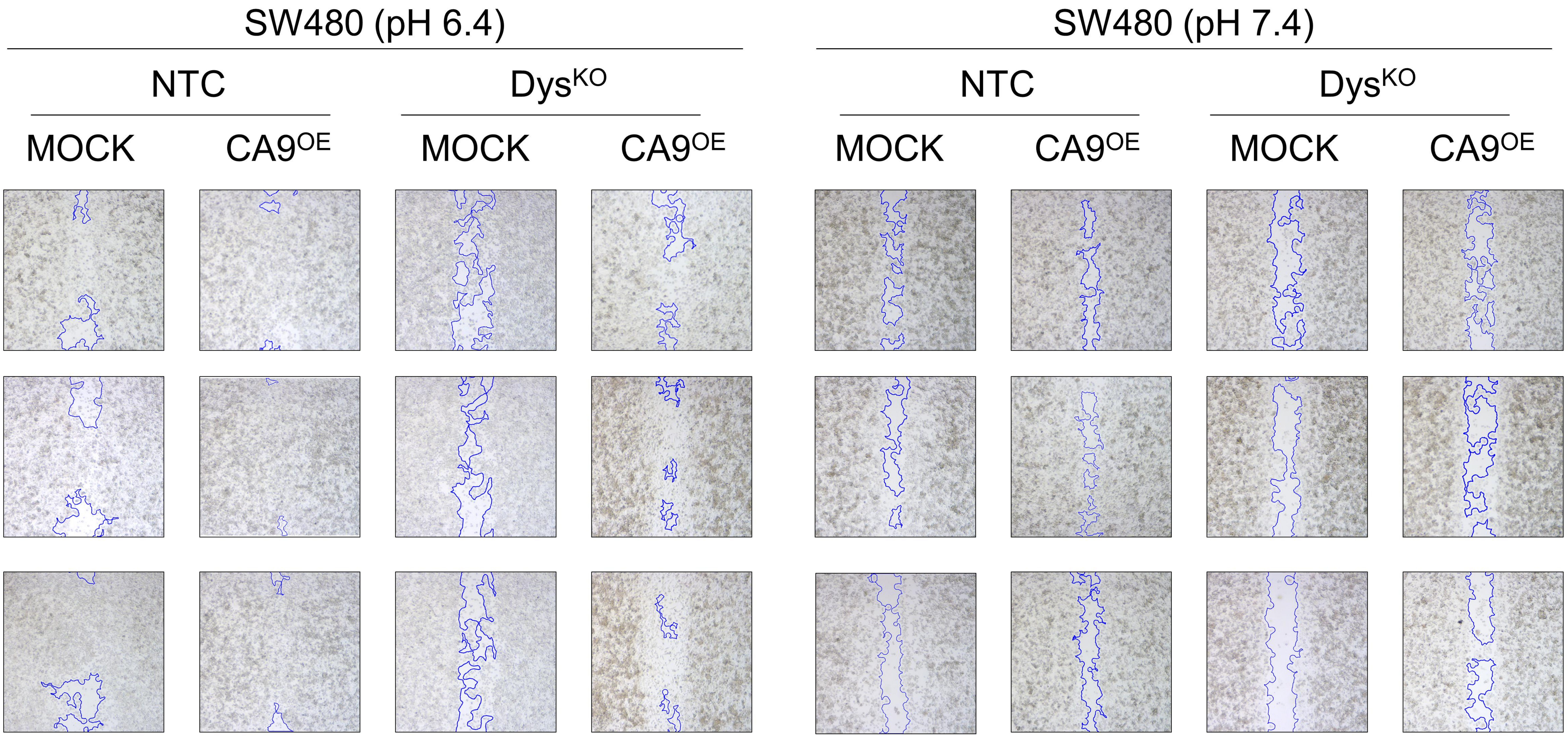

Fig 8a - Migration

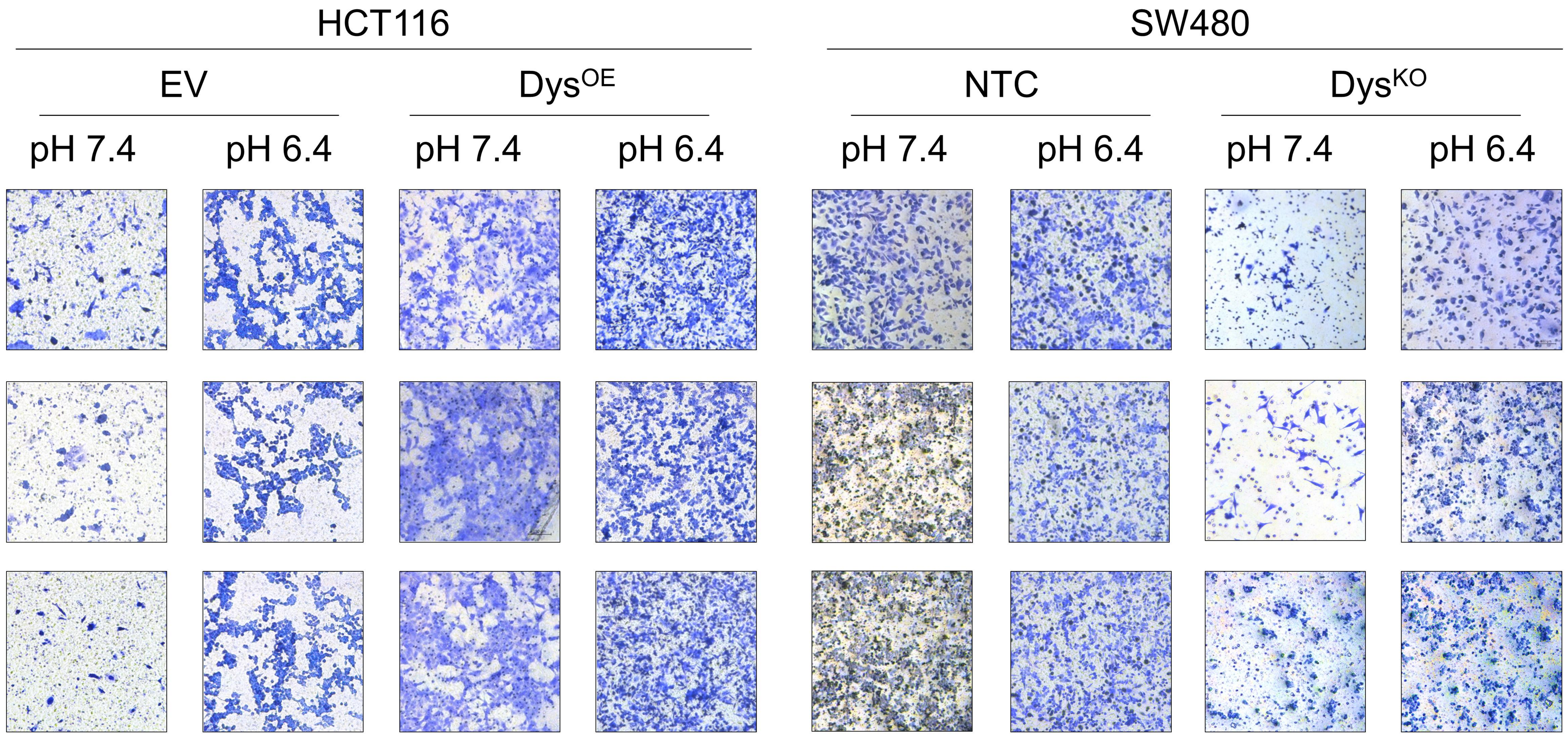

Fig. 8b - Migration

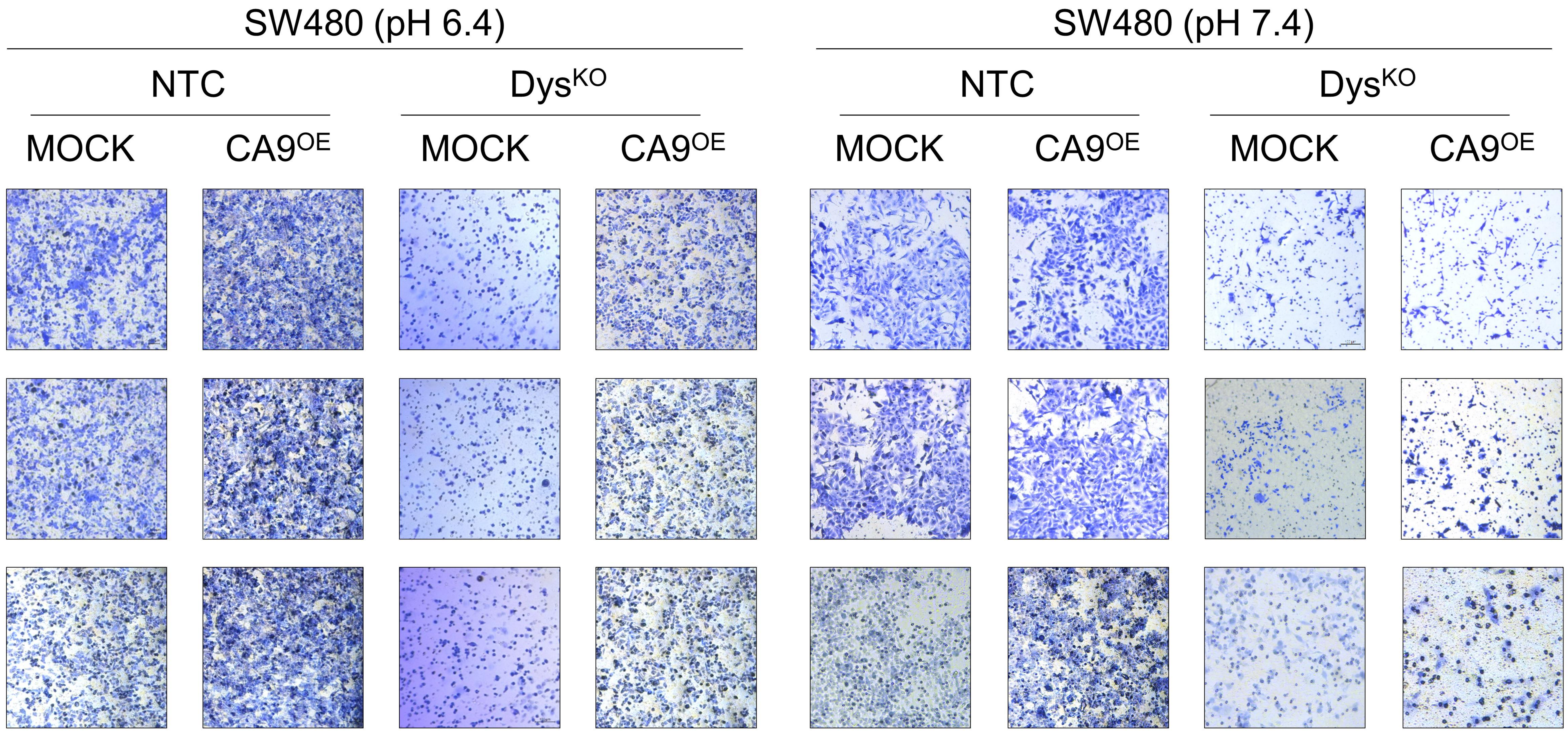

Fig 8a - Invasion

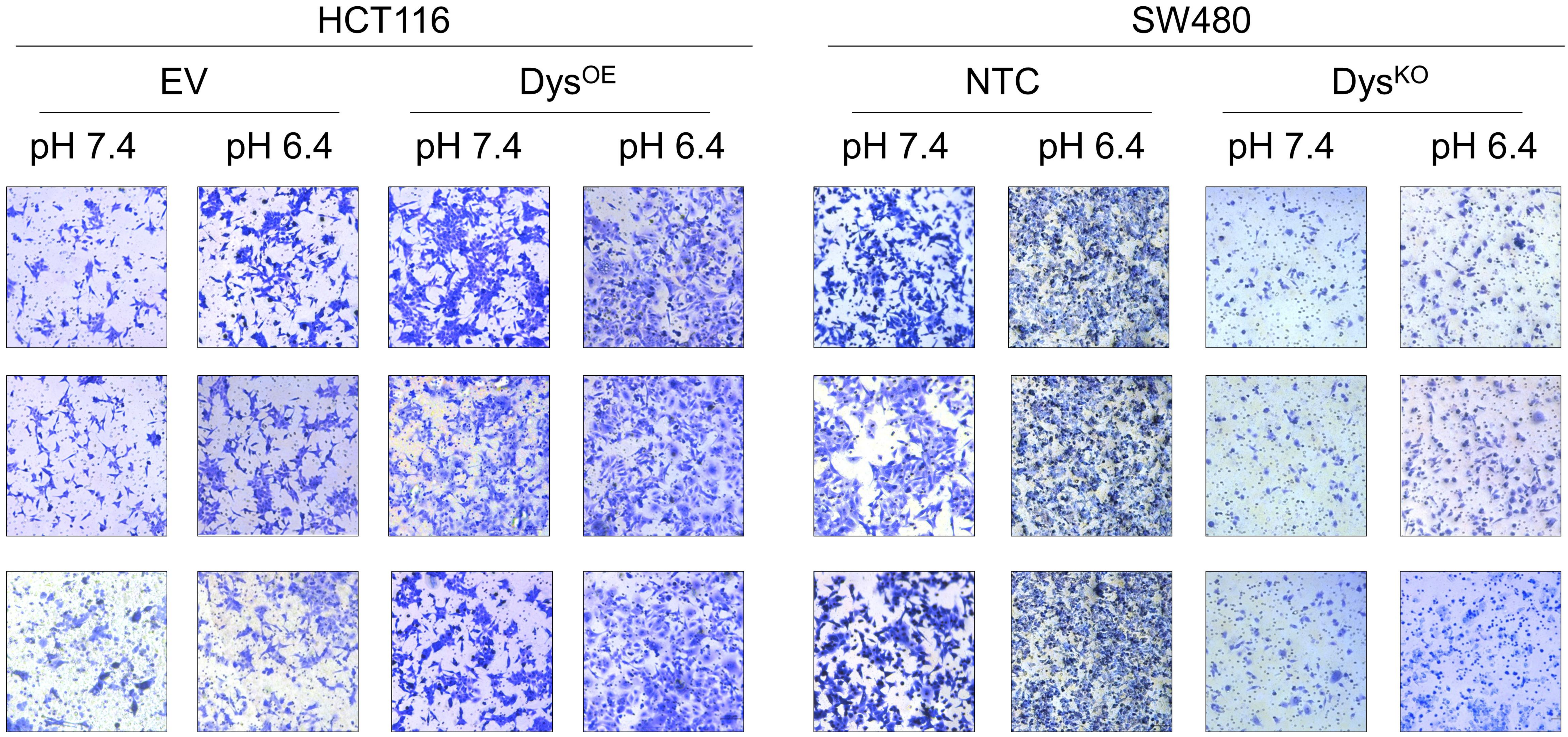

Fig. 8b - Invasion

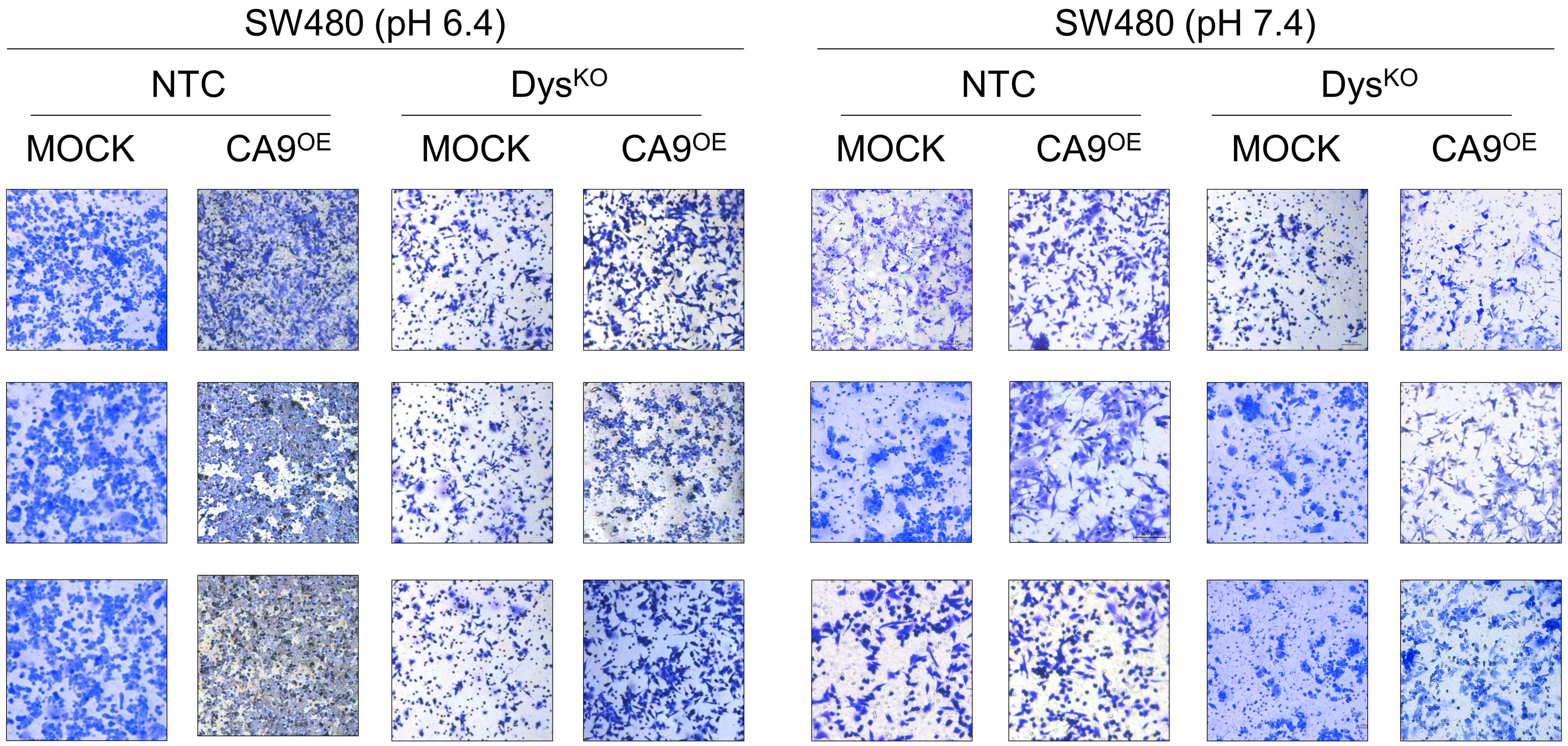

Supplement: Supplementary file 2 — Unprocessed Data [file 41392_2025_2543_MOESM2_ESM.pdf]
