# Supplementary material for: The dysadherin/carbonic anhydrase 9 axis shapes an acidic tumor microenvironment to promote colorectal cancer progression
Source: Signal Transduct Target Ther. 2026 Jan 15;11:19. doi: 10.1038/s41392-025-02543-x (PMC12804794; doi:10.1038/s41392-025-02543-x)

Figure 2e

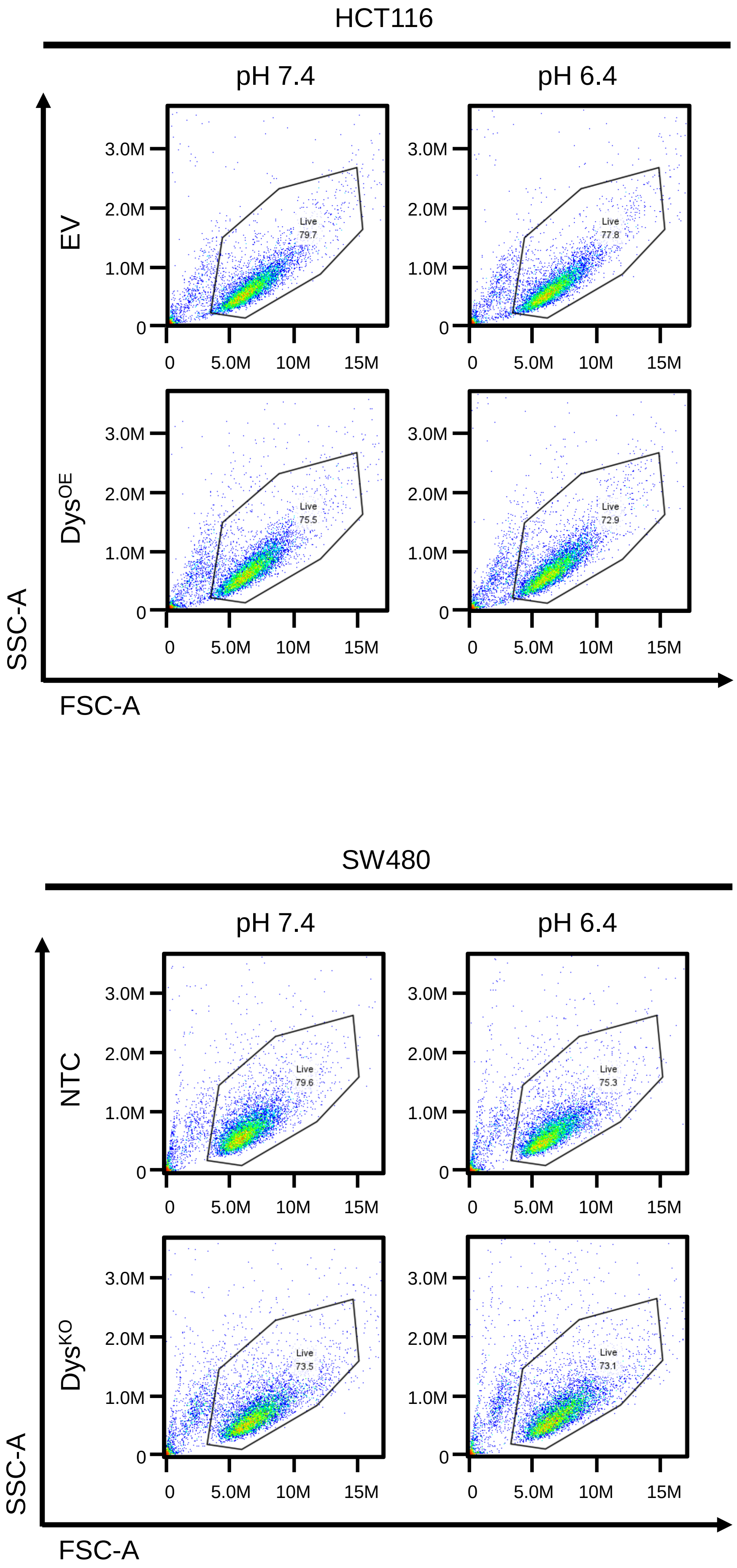
HCT116
pH 7.4
pH 6.4
EV
Dys^{OE}
Live 79.7
Live 77.8
Live 75.5
Live 72.9
3.0M
2.0M
1.0M
0
5.0M
10M
15M
SSC-A
FSC-A
SW480
pH 7.4
pH 6.4
NTC
Dys^{KO}
Live 79.6
Live 75.3
Live 73.5
Live 73.1
SSC-A
FSC-A

Supplementary Figure 2i

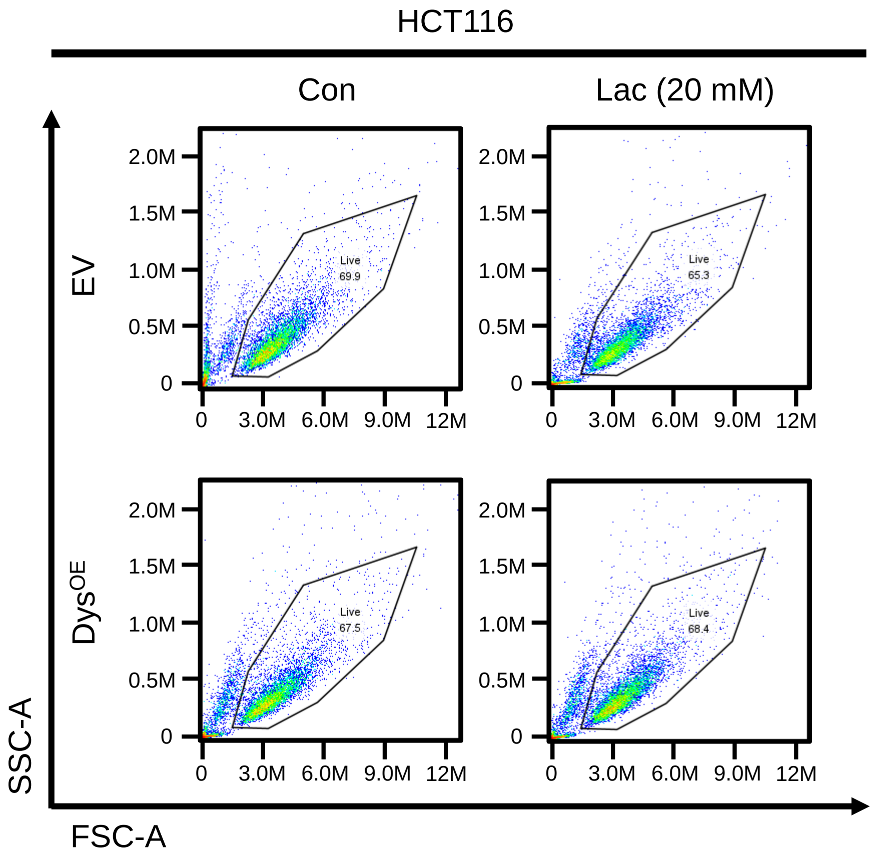

Supplementary Figure 2j

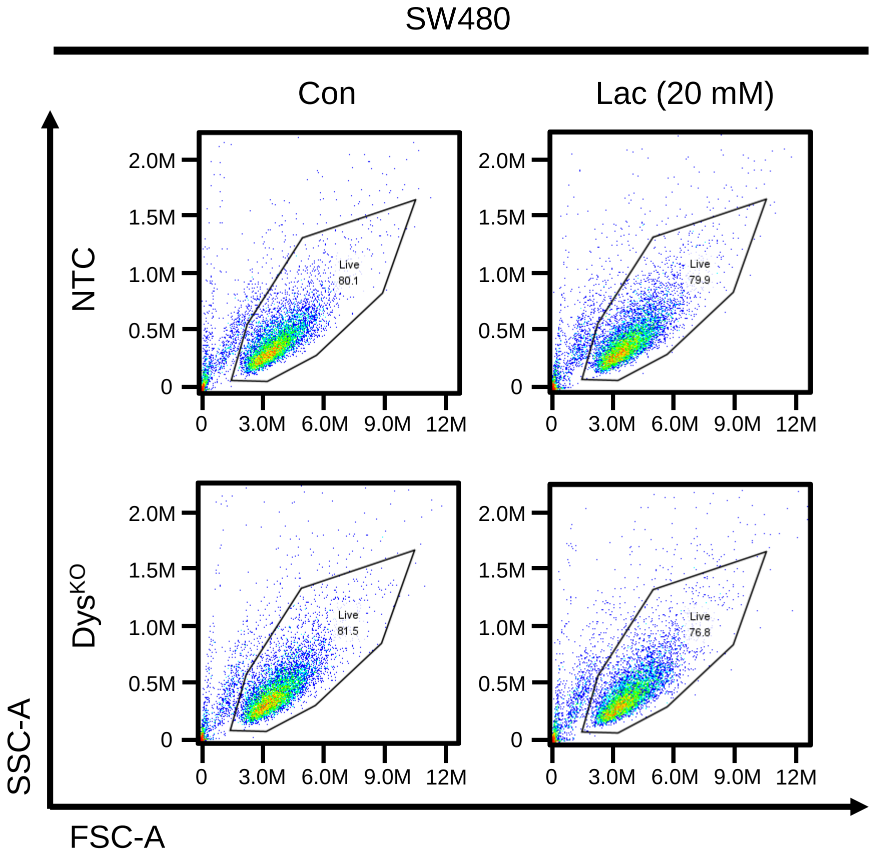

Figure 5d

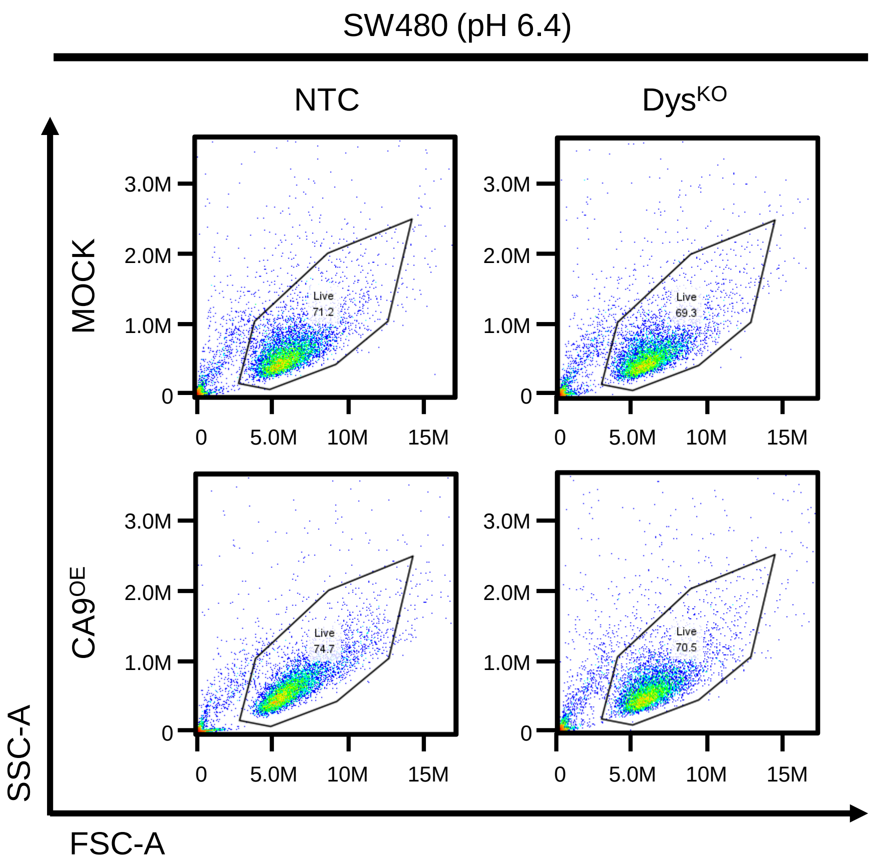

Figure 5i

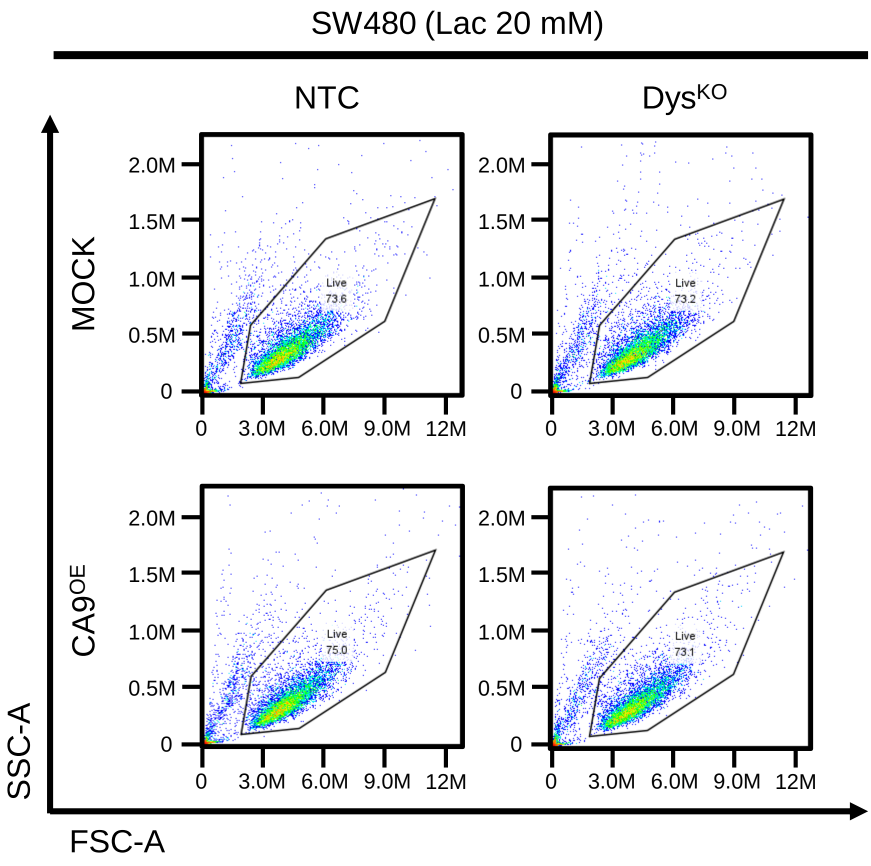

Supplementary Figure 5b - 1

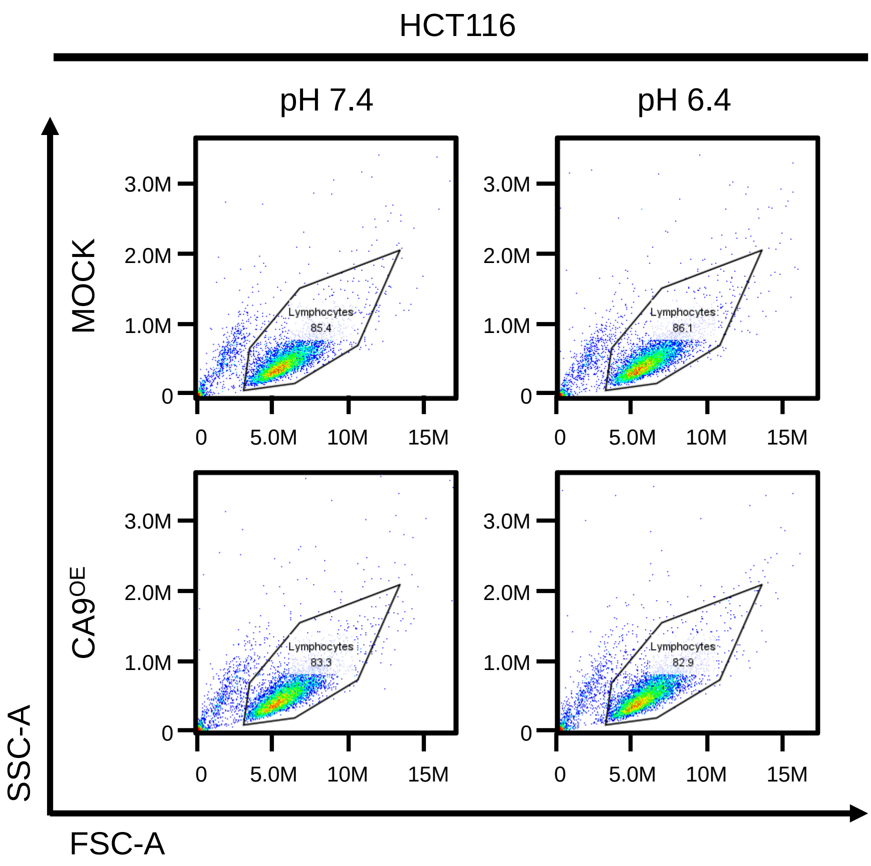

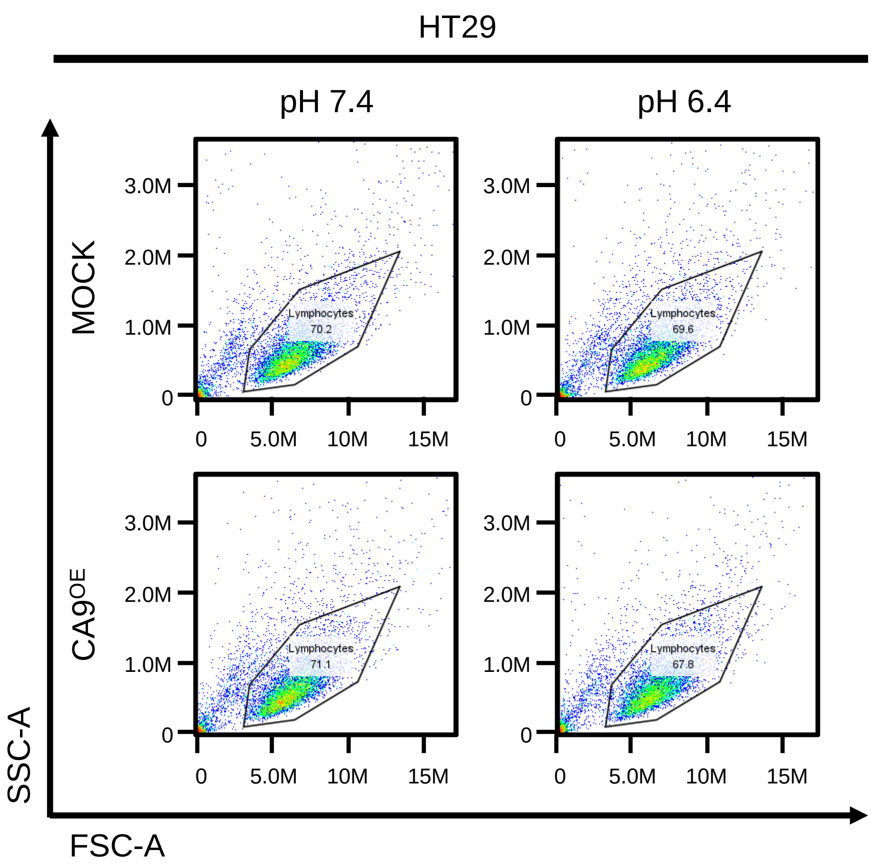

Supplementary Figure 5b - 2

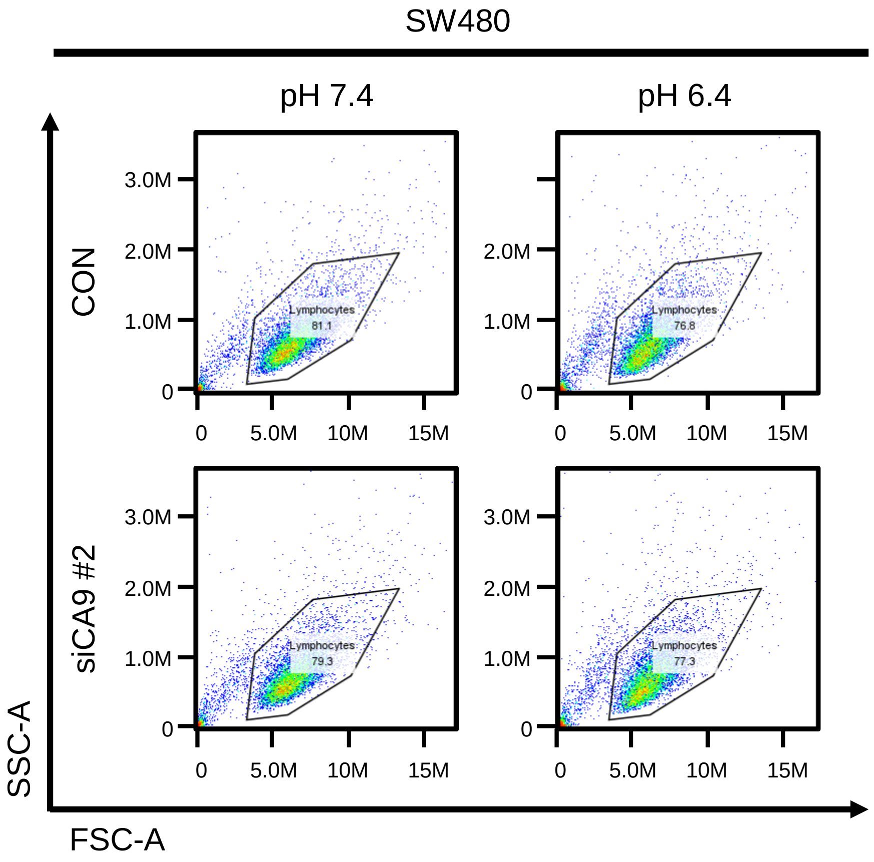

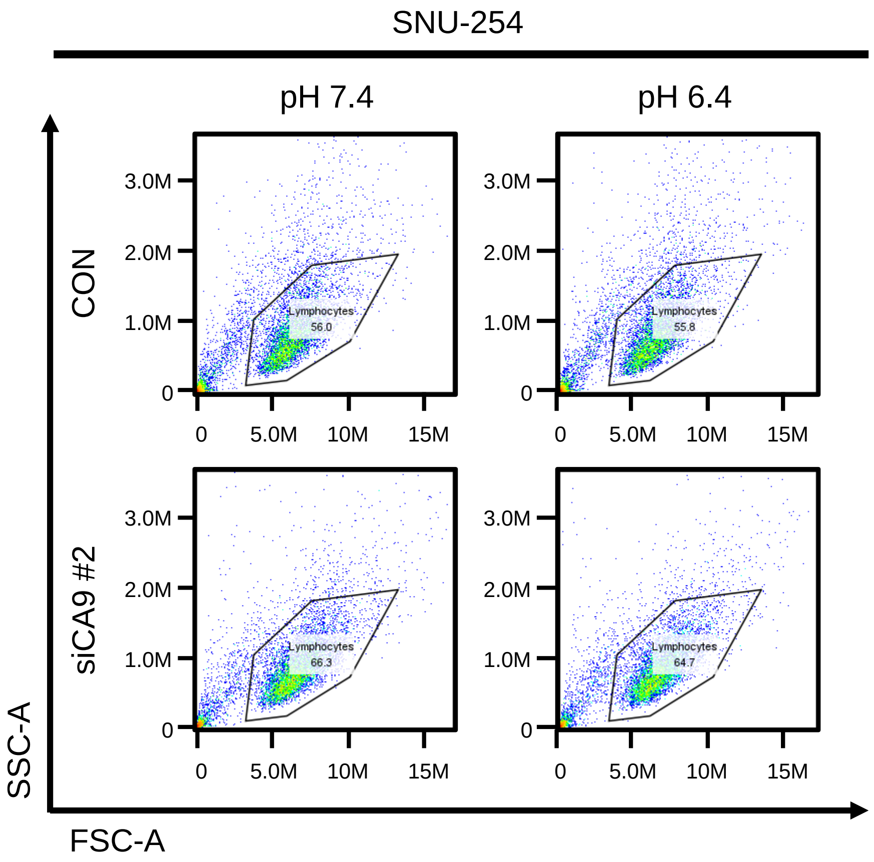

Supplementary Figure 5b - 3

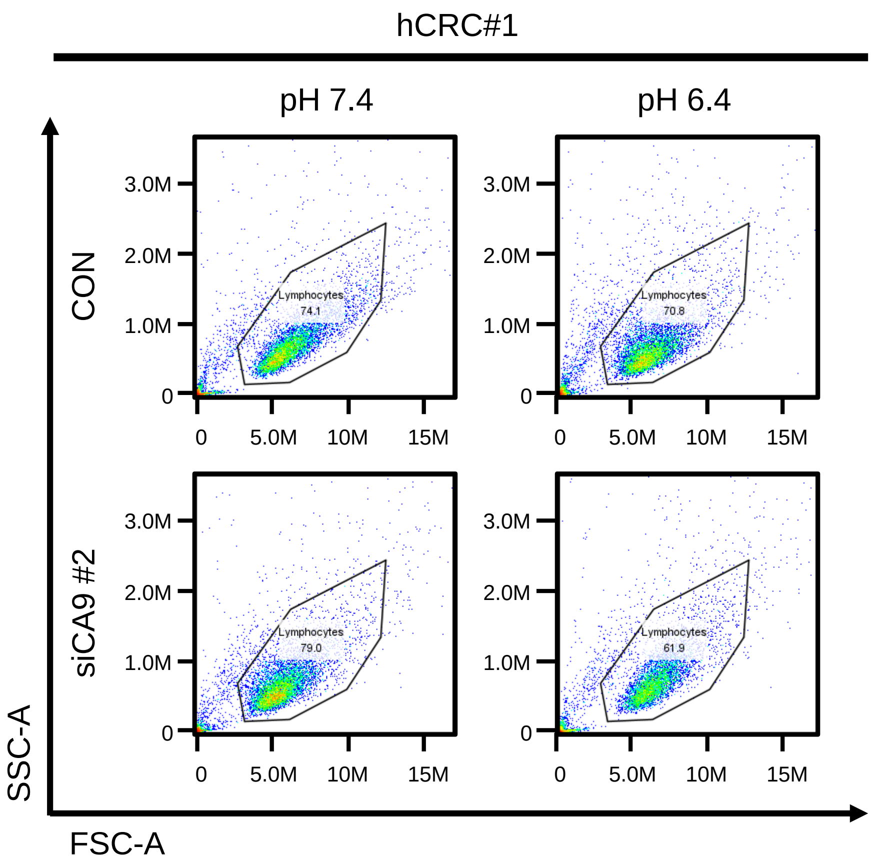

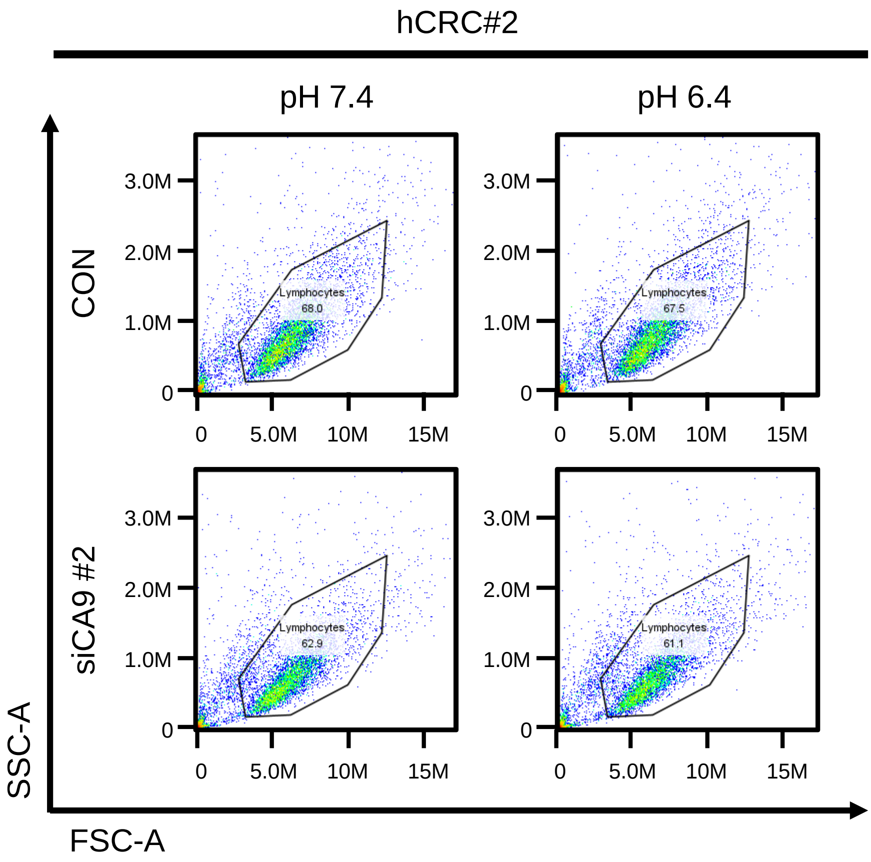

Supplementary Figure 5g

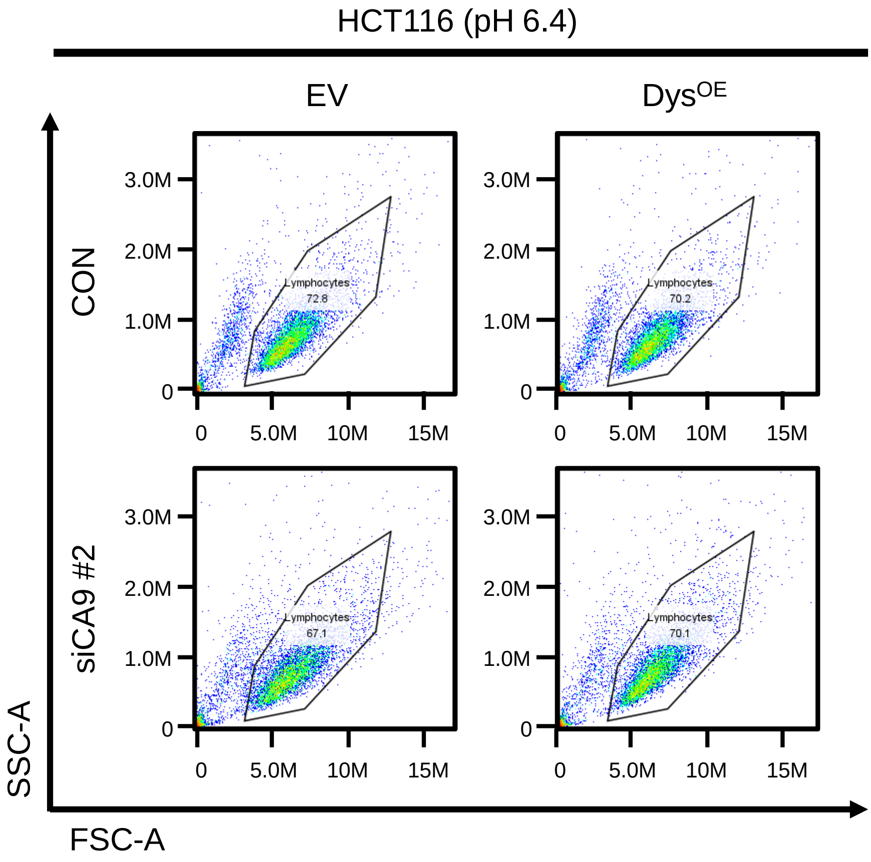

Supplementary Figure 5q

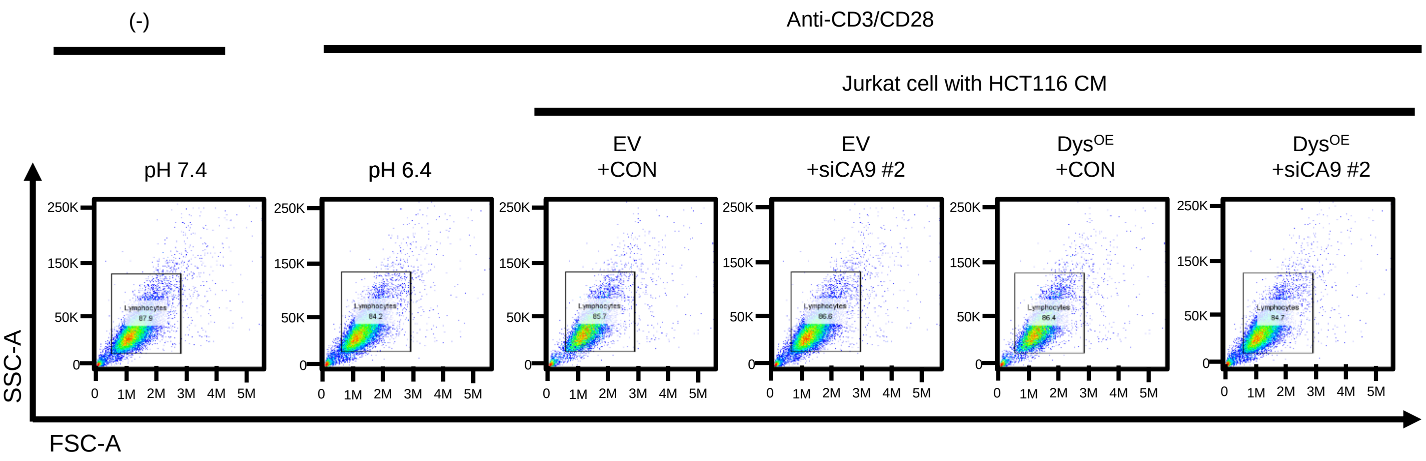

Supplementary Figure 5r

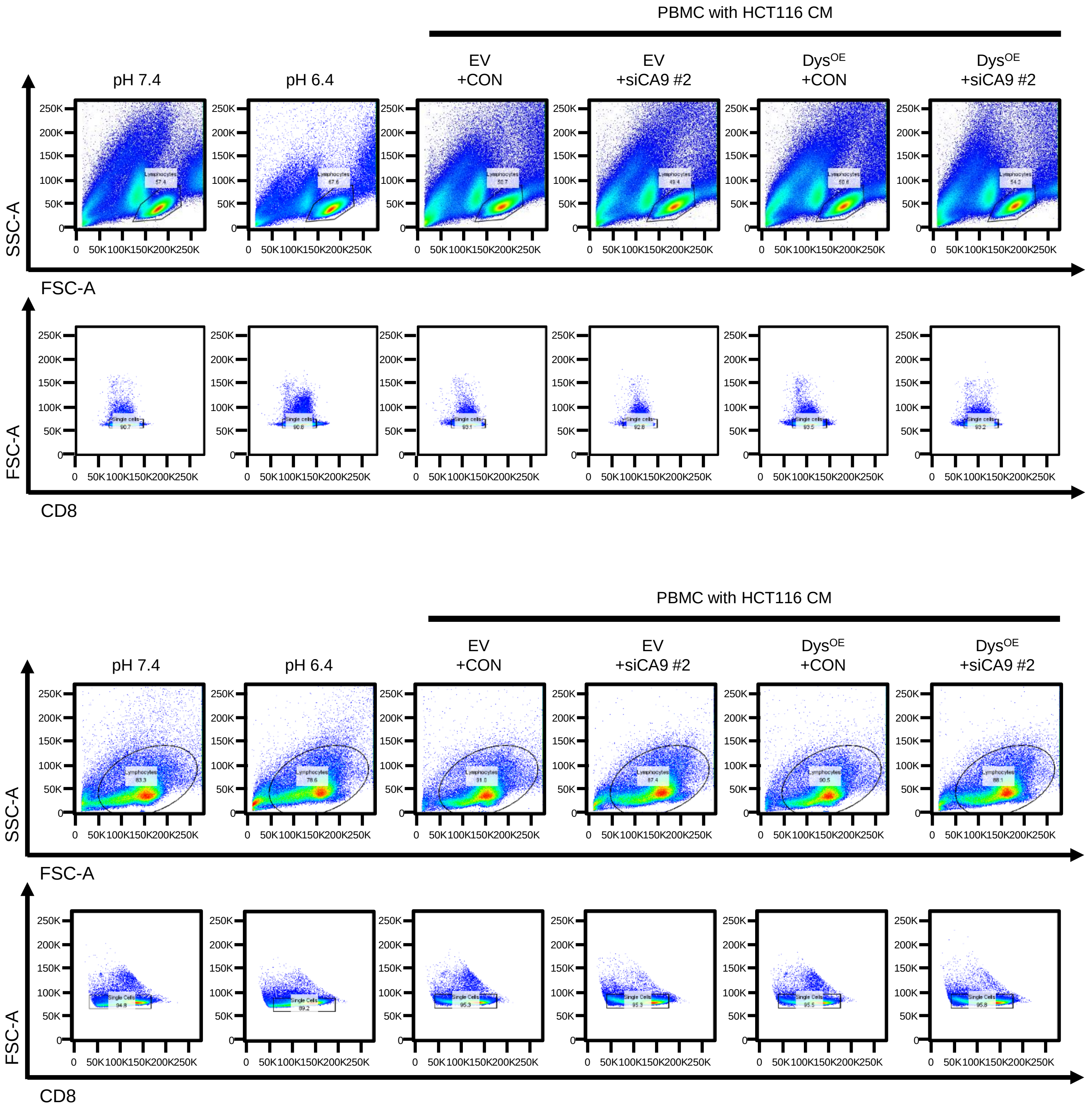

Supplement: Supplementary file 5 — FACS gating strategy [file 41392_2025_2543_MOESM5_ESM.pdf]
