# Supplementary material for: The dysadherin/carbonic anhydrase 9 axis shapes an acidic tumor microenvironment to promote colorectal cancer progression
Source: Signal Transduct Target Ther. 2026 Jan 15;11:19. doi: 10.1038/s41392-025-02543-x (PMC12804794; doi:10.1038/s41392-025-02543-x)
Supplement: Supplementary file 1 — Supplementary materials [file 41392_2025_2543_MOESM1_ESM.docx]

Supplementary Materials for

The dysadherin/carbonic anhydrase 9 axis shapes an acidic tumor microenvironment to promote colorectal cancer progression

Choong-Jae Lee^1#^, Hyeon-Ji Yun^1#^, Tae-Young Jang^1^, So-El Jeon^1^, Yeong-Hoon Cho^1^, Da-Ye Lim^1^, Eun-Ju Han^1^, Sun-Young Kong^2,3,4^, Jeong-Seok Nam^1*^.

^1^ Department of Life Sciences, Gwangju Institute of Science and Technology, Gwangju 61005, Republic of Korea

^2^ Targeted Therapy Branch, Research Institute, National Cancer Center, Goyang 10408, Republic of Korea

^3^ Cancer Biomedical Science, National Cancer Center, Goyang 10408, Republic of Korea

^4^ Department of Laboratory Medicine, National Cancer Center, Goyang 10408, Republic of Korea

\# These authors contributed equally to this work

* Correspondence to: namje@gist.ac.kr

**This PDF file includes:**

Materials and Methods

Figures. S1 to S12

Tables S2, S3, and S5 to S8
**Materials and Methods**

**Histological assessment**

For clinical analysis, tissues from 10 patients with liver metastasis and 10 patients with carcinoma *in situ* were embedded in optimal cutting temperature (OCT) compound (Leica Biosystems, Nussloch, Germany), cut at 10 μm using a cryostat (HM525 NX, Thermo Fisher Scientific, Waltham, MA, USA) and processed for immunofluorescence (IF) staining. The biospecimens and data used for this study were provided by the Biobank of Chonnam National University Hwasun Hospital, a member of the Korea Biobank Network.

For the mouse models, all liver tissue samples were embedded in OCT compound (Leica Biosystems). Frozen tissues were sectioned with a cryostat to obtain 10 μm thick sections and immunostained by IF. For evaluation of tissue morphology and quantify the metastatic burden at the microscopic level, cryosections were stained with hematoxylin (Dako, Carpinteria, CA, USA) and eosin (Millipore, Billerica, MA, USA) following the manufacturers' protocols.

In both clinical and mouse tissues, target proteins were visualized by IF. The target proteins were visualized using the specific secondary antibodies conjugated with the fluorescent dyes described in Table S6. All the nuclei were stained with 4′,6-diamidino-2-phenylindole (DAPI, Sigma‒Aldrich, St. Louis, Missouri, USA). The fluorescence signals were visualized using a research slide scanner VS200 (Olympus, Tokyo, Japan) or Axio Imager 2 (Carl Zeiss, Oberkochen, Germany) at a total magnification of 100x or 200x. Relative expression levels of the target protein on the basis of fluorescence intensity were automatically quantified with Image-Pro Premier 11 software (Media Cybernetics, Inc., Rockville, MD, USA) and normalized to the DAPI intensity.

**Clinical analysis**

Tissue microarray (TMA) slides from 178 patients with colorectal cancer (CRC) were immunostained to detect target proteins using the specific antibodies described in Table S6. Dysadherin was detected using a specific monoclonal antibody (M53) as previously reported ^1^. The TMA slides contained 3 tumor tissue cores and 2 matched normal tissue cores from each patient. After heat-induced epitope retrieval, the slides were permeabilized and incubated with primary antibodies (1:500) at 4 °C overnight. After repeated washing steps, the slides were incubated with a secondary antibody (1:1000) for 1 h at room temperature. All the nuclei were stained with DAPI. The fluorescence signals were visualized with a research slide scanner VS200 (Olympus). The expression of the target was automatically quantified with Image-Pro Premier 11 software (Media Cybernetics, Inc.) in a blinded manner without any clinical information. Three spots per core were randomly selected, and the intensity of the protein was calculated by multiplying the area and average density. Patients were divided into two groups according to the median dysadherin, LAMP2 or carbonic anhydrase 9 (CA9) level: low (< 50%, *n* = 61) and high (≥ 50%, *n* = 62). recurrence-free survival (RFS) was defined as the time from surgery to either recurrence or death, whichever occurred first, with patients who remained alive at the last follow-up being censored if neither event had occurred by the time of analysis. overall survival (OS) was measured from the time of disease diagnosis to death from any cause, with patients still alive at the last follow-up being censored at that point. Survival rates were estimated using the Kaplan‒Meier method, and comparisons between groups were performed using log-rank tests. All the statistical analyses were conducted using GraphPad Prism (GraphPad Software, Inc., San Diego, CA, USA).

***Apc*^Min/+^ mouse tumor-derived organoid culture**

Single cells were isolated from the intestinal tumors of 24-week-old *Apc*^Min/+^;*Fxyd5*^+/+^ and *Apc*^Min/+^;*Fxyd5*^-/-^ mice and cultured as described in a previous report with slight modifications ^2^. Briefly, mouse intestines containing tumors were incubated with ethylenediaminetetraacetic acid (EDTA) chelation buffer (EDTA 3mM, Dithiothreitol 50 μM in PBS) for 60 min on ice. After chelation, the detached normal intestinal epithelial cells were removed by centrifugation; tumor cells remained attached to the mesenchyme. Intestinal fragments with tumor cells were then dissociated with collagenase as described in the previous report ^2^. The isolated tumor cells were counted and pelleted, and a total of 2,000 cells were seeded in 96-well plates or mixed with 10 μL of Matrigel (Corning Matrigel® Growth Factor Reduced Basement Membrane Matrix, #356231, Corning, NY, USA). After the polymerization of Matrigel, 200 μL of IntestiCult™ Organoid Growth Medium (Mouse, #06005, STEMCELL Technology, Vancouver, BC, Canada) was added. Tumoroids were subsequently used for pHrodo staining and the HPTS assay.

**Bioinformatic analysis**

Gene set enrichment analysis (GSEA) was conducted as described in our previous study ^3^. In summary, a list of A list of differentially expressed genes (DEGs) was generated from the gene expression omnibus (GEO) database using the GSE106582 dataset, which compared normal tissues (*n* = 117) and CRC tumor tissues (*n* = 77) including 68 matched tumor-mucosa pairs. DEGs with a *p* value < 0.05 were used to create a ranked gene list. GSEA of the ranked gene list was conducted using the Java implementation of GSEA obtained from http://www.broadinstitue.org/gsea/(1,000 permutations; minimum term size: 15; maximum term size: 500; C2: all curated genes; C5: all ontology genes). The normalized enrichment score was used to adjust for gene set size variations, and a false discovery rate (q value) was applied as the significance cutoff.

For single cell RNA sequencing (scRNA-seq) data analysis, we reanalyzed the GSE144735 dataset using the Seurat package. This analysis included normalization, identification of variable features, data scaling, clustering, and generation of uniform manifold approximation and projection (UMAP) plots using default parameters. Cell type annotations were adopted from the original dataset, which classified epithelial cells as normal or tumor tissue. Gene classification was performed using the Enrichr web server (https://maayanlab.cloud/Enrichr/) via the Reactome 2023 ontology. For trajectory analysis, Monocle 3 was employed to reconstruct the cell differentiation trajectories. This algorithm maps cells along a trajectory corresponding to a biological process, such as cell differentiation, using an unsupervised approach that leverages the asynchronous progression of individual cells.

For the database for annotation, visualization, and integrated discovery (DAVID) bioinformatic analysis, we generated a list of DEGs using the GSE21510 dataset, which compared CA9-high patients (*n* = 78) and CA9-low patients (*n* = 78). DEGs with a *p* value < 0.05 were used to create a gene list. DAVID bioinformatics was conducted using a web-based functional annotation platform (https://davidbioinformatics.nih.gov/).

**RNA-seq and ingenuity pathway analysis (IPA)**

RNA-seq using dysadherin-knockout (KO) SW480 cells and control (non-targeting control, NTC) SW480 cells was performed as previously described ^3^. Briefly, samples were prepared in biological triplicates and RNA-seq analysis was conducted by LAS Science (Seoul, Republic of Korea). mRNA libraries were generated using the TruSeq Stranded mRNA Sample Preparation Kit and sequenced on the Illumina NextSeq platform.

The list of DEGs (*n* = 4,456, *p* < 0.05) between dysadherin KO SW480 cells and control cells was subjected to IPA (Qiagen, Redwood City, CA, USA) to identify potential diseases, functions, and upstream regulators that are significantly associated with dysadherin KO.

**Establishment of CA9-overexpressing (OE) cell lines**

For establishment of the CA9 OE cell line, NTC and dysadherin KO SW480 cells were transfected with a CA9 expression vector (pCMV-CA9) and selected with hygromycin B for 1 week. Dilution cloning was performed, and expression was confirmed by real-time reverse transcriptase quantitative polymerase chain reaction (RT‒qPCR) and immunoblot analysis.

**Gene expression modification via siRNA**

The cells were transfected with small interfering RNA (siRNAs) targeting dysadherin or CA9 (Bioneer, Daejeon, Republic of Korea) in Opti-MEM (Gibco, Billings, Montana, USA) with Lipofectamine^TM^ 2000 (Invitrogen, Carlsbad, MA, USA) according to the manufacturer’s instructions. The knockdown efficacy was confirmed by measuring mRNA expression via RT‒qPCR. The sequences of the siRNAs used are listed in Table S7.

**Proliferation (BrdU) assay**

A total of 3 × 10^3^ cells were seeded on 96-well plates and incubated at 37 °C and 5% CO_2_ for 24 h. After 24 h, the cells were treated with pH-adjusted media supplemented with 5% fetal bovine serum (FBS) and incubated at 37 °C in a CO_2_-free incubator for 24 h. Cell proliferation was measured using an enzyme-linked immunosorbent assay (ELISA) colorimetric BrdU kit (Roche, Mannheim, Germany) based on the incorporation of BrdU (20 μmol/L) for 2 h. The absorbance was read at a wavelength of 370 nm using an Epoch microplate reader (BioTek, Winooski, VT, USA).

**Clonogenicity assay**

A clonogenicity assay was performed as described in our previous report. Briefly, cells were seeded in 6-well plates (1000 cells/well) and incubated at 37 °C and 5% CO_2_ for 24 h. Once the cells were attached, they were treated with pH-adjusted media supplemented with 5% FBS and incubated at 37 °C in a CO_2_-free incubator for 24 h. After 24 h, the medium was exchanged for regular medium, and then, the cells were incubated in 5% CO_2_ conditions for 10 days. Colonies greater than 50 μm in size were counted after staining with crystal violet (*n* = 3/group).

**Apoptosis assay**

The proportion of apoptotic cells was quantitatively analyzed via apoptosis assays via an Annexin V−Fluorescein Isothiocyanate (FITC) Apoptosis Detection Kit I (BD Biosciences, Franklin Lakes, New Jersey, USA). The cells were collected and washed twice with PBS. Then, 100 μl of the suspension was transferred to a tube, to which 5 μl of FITC, annexin V, and propidium iodide were added. The mixture was incubated at room temperature for 15 min in the dark. After incubation, 400 μl of binding buffer was added, and the cells were analyzed by flow cytometry (BD Biosciences). Fluorescence-activated cell sorting (FACS) data were analyzed using FlowJo software (TreeStar, Ashland, OR, USA).

**Sphere formation assay and in vitro limiting dilution assay (LDA)**

For the sphere formation assay, cells were seeded at various cell densities and incubated under sphere culture conditions. The number of spheres was subsequently counted, and their size was measured. Sphere size was analyzed by measuring the sphere diameter using Image-Pro Premier 11 software (Media Cybernetics, Inc.). Growth was analyzed by Cell Titer-Blue quantification (Promega, Madison, WI, USA) (*n* = 3).

For analysis of their self-renewal ability, the cells were pretreated with pH-adjusted media for 24 h and seeded under sphere culture conditions (poly-HEMA-coated plates; Sigma‒Aldrich) at various cell densities of 10, 100, 200, 300, 400, and 500. After 7 days of culture, the number of wells with spheres was counted (*n* = 12) and analyzed by the extreme LDA webtool (http://bioinf.wehi.edu.au/software/elda). *p* values indicate the statistical significance of differences in stem cell frequencies between the two groups.

**Wound-healing assay**

Cells were seeded into culture inserts (Ibidi, GmbH, Martinsried, Germany) and subjected to a wound scratch assay. Following scratch induction, cells were incubated for 48 h (endpoint). Phase-contrast images were captured using a microscope equipped with a camera (Carl Zeiss) at 100× total magnification. The wound area at baseline (0 h) and at the endpoint was quantified using Image-Pro Premier 11 software (Media Cybernetics, Inc.). Wound closure was expressed as the percentage reduction relative to the initial wound area. To minimize the potential confounding effect of differential cell proliferation, cycloheximide, a protein synthesis inhibitor that blocks mitotic entry, was added to the culture medium during the wound closure period.

**Boyden chamber assay**

Migration and invasion assays were performed using transwell chambers with 8 μm pore size membranes (Corning). For the migration assay, 3 × 10^5^ cells were seeded into the upper chamber in serum-free medium. For the invasion assay, an equivalent number of cells were seeded into Matrigel-coated transwell inserts under the same serum-free conditions. The lower chamber was filled with medium containing 20% FBS as a chemoattractant. After 24 h of incubation at 37 °C, cells that had migrated or invaded through to the lower surface of the insert membrane were fixed, stained with crystal violet, and counted under a phase-contrast microscope (Carl Zeiss). All experiments were performed in biological triplicates.

**RT‒qPCR**

Total RNA was isolated using TRIzol reagent (TaKaRa Biomedicals, Kusatsu, Japan). The purity of the RNA was verified by measuring the 260/280 and 260/230 absorbance ratios. cDNA templates were synthesized from 0.5 μg of total RNA using the PrimeScript^TM^ 1st Strand cDNA Synthesis Kit (TaKaRa Biomedicals) with random primers. Power SYBR Green PCR Master Mix and Step-One Real-time PCR systems (Applied Biosystems, Foster City, CA, USA) were used for the PCR amplification of cDNAs. The primers used for RT‒qPCR are listed in Table S8.

**Protein Isolation and immunoblot analysis**

Tissues or cells were homogenized in Radio Immuno Precipitation Assay (RIPA) buffer for 20 min on ice. A bicinchoninic acid (BCA) assay kit was used to determine protein concentrations using a BCA assay (Thermo Fisher Scientific). Proteins were denatured with Sodium Dodecyl Sulfate (SDS) (Sigma‒Aldrich) by boiling at 95 °C for 5 min. Equal amounts of total protein (10 μg) were separated by 10% polyacrylamide gel electrophoresis (PAGE), and separated proteins were transferred to a polyvinylidene difluoride membrane (Millipore). The membranes were blocked with 10% bovine serum albumin (Sigma‒Aldrich) and incubated overnight at 4 °C with the indicated primary antibodies. The membrane was then treated with secondary antibodies conjugated to horseradish peroxidase (HRP). HRP chemiluminescence was detected with SuperSignal West Dura Extended Duration Substrate (Thermo Fisher Scientific) and a digital 8 imaging system (ProteinSimple, 3040 Oakmead Village Drive, Santa Clara). The antibodies used for immunoblot analyses are listed in Table S6.

**Thiazolyl blue tetrazolium bromide (MTT) assay for IC_50_ determination**

For determination of the IC_50_ values of volociximab, PND-1186, and Stattic, 5 × 10^3^ cells were seeded in 96-well plates and treated with various concentrations of volociximab, PND-1186, and Stattic. 48 h later, the cells were treated with MTT (Sigma‒Aldrich) in serum-free media for 1 h. Afterward, the MTT was removed, and dimethyl sulfoxide (DMSO; Sigma‒Aldrich) was added to each well. The cell viability was determined according to the absorbance at a wavelength of 420 nm, which was measured using an Epoch microplate reader (BioTek).

**Luciferase reporter assay**

The luciferase reporter assay was performed using a vector containing the CA9 promoter region (Genecopoeia, Maryland, USA). A mixture of vector, β-galactosidase, and Lipofectamine was made at a ratio of 1 μg:1 μg:2 μl in 300 μl of OptiMEM medium/well. 24 h later, the cells were transfected with the vector, and the cells were treated with PND-1186 or Stattic. Cell lysis buffer was added after 24 h of treatment. For measurement of luciferase activity, a Renilla luciferase assay system (Promega) and a SpectraMax L (Molecular Devices, San Jose, CA, USA) were used according to the manufacturer’s recommendation. Relative luciferase activity was calculated by normalizing the activity of the target reporter to that of the β-galactosidase reporter.

**Chromatin immunoprecipitation (ChIP) assay**

The EZ ChIP Kit (Millipore) was used according to the manufacturer’s instructions. For ChIP assays, 6 × 10^6^ cells in culture were fixed in 1% formaldehyde to crosslink DNA to bound proteins, and the reaction was quenched by the addition of 0.125 M glycine. The cells were washed with PBS and collected at 4 °C in the presence of a protease inhibitor cocktail (Sigma‒Aldrich). The collected cells were resuspended in 500 μl of ChIP sonication buffer (20% SDS and protease inhibitors), followed by sonication and centrifugation of fragments (200–700 bp long) at 2000 ×g. The fragmented chromatin supernatant was immunoprecipitated using an anti-STAT3 antibody at 4 °C overnight. Protein A agarose beads were added to the chromatin‒antibody complex and centrifuged to sediment the beads. The beads were washed with cold buffer, and the DNA was eluted with elution buffer. The DNA in the supernatant was precipitated using a high-salt method according to the manufacturer's recommendations. The extracted DNA was purified using a kit, and 10 μl of the purified DNA was used for PCR amplification with specific primers designed around the STAT3 binding site of the CA9 promoter. The binding site of STAT3 on the CA9 promoter was amplified, as shown in Table S8.

**Formation of 3D spheroid**

A total of 1 × 10^4^ cells in 50 μl of media were seeded into 96-well plates pre-coated with poly (2-hydroxyethyl methacrylate) (poly-HEMA). Subsequently, 50 μl of 10% Matrigel (Corning Matrigel® Basement Membrane Matrix, #354234, Corning) diluted in media was gently added on ice to prevent premature gelation. Then, the plate with cells and Matrigel incubated for 48 h at 37 °C with 5% CO_2._

**Extracellular Acidification Rate (ECAR) and Oxygen Consumption Rate (OCR) measurement**

A total of 3 × 10^4^ cells were seeded on cell culture microplates and incubated at 37 °C and 5% CO_2_ for 24 h. After 24 h, the cells were treated with pH-adjusted media supplemented with 5% FBS and incubated at 37 °C in a CO_2_-free incubator for 24 h. The ECAR was measured using Seahorse XF Glycolysis Stress Test Kit (Agilent, Santa Clara, CA, USA) and OCR was measured using Seahorse XF Cell Mito Stress Test Kit (Agilent). The assays were performed according to the manufacturer’s protocol. The ECAR and OCR were measured using a Seahorse XF HS mini analyzer (Seahorse Biosciences, North Billerica, MA, USA).

**Adenosine triphosphate (ATP) production measurement**

A total of 6 × 10^5^ cells were seeded on 6 well plates and incubated at 37 °C and 5% CO_2_ for 24 h. After 24 h, the cells were treated with pH-adjusted media supplemented with 5% FBS and incubated at 37 °C in a CO_2_-free incubator for 24 h. The ATP concentration was determined using an ATP assay kit (Abcam, Waltham, MA, USA) according to the manufacturer’s protocol. The absorbance was read at a wavelength of 570 nm using an Epoch microplate reader (BioTek).

**Intracellular reactive oxygen species (ROS) measurement**

A total of 6 × 10^5^ cells were seeded in 6-well plates and incubated at 37 °C and 5% CO_2_ for 24 h. After 24 h, the cells were treated with pH-adjusted media supplemented with 5% FBS and incubated at 37 °C in a CO_2_-free incubator for 24 h. Intracellular ROS levels were measured using a Total ROS Assay Kit 520 nm (Invitrogen) according to the manufacturer’s protocol. The fluorescence signals were quantified using flow cytometry analysis.

**Mitochondria ROS measurement**

A total of 8 × 10^3^ cells were seeded in 96-well plates and incubated at 37 °C and 5% CO_2_ for 24 h. After 24 h, the cells were treated with pH-adjusted media supplemented with 5% FBS and incubated at 37 °C in a CO_2_-free incubator for 24 h. Mitochondria ROS levels were measured using a MitoSOX™ Green Mitochondrial Superoxide Indicators (Invitrogen) according to the manufacturer’s protocol. The cells were incubated with MitoSOX™ Green, and the excitation/emission wavelengths were read at 488/510 nm using a Varioskan LUX Multimode Microplate Reader (Thermo Fisher Scientific).

**Indirect co-culture assay**

T-cell activation was evaluated using an indirect co-culture system with conditioned media (CM). To prepare the CM, HCT116 Empty-vector (EV) and dysadherin OE cells were transfected with siRNA targeting CA9. After 48 h, cell-free CM was collected by centrifugation and stored at –80 °C until use.

Jurkat cells (4 × 10⁵) were seeded in pH 7.4 or pH 6.4 media, or in CM, and incubated to stabilize for 1 h. T-cell activation was then induced by the addition of soluble anti-CD3(e-bioscience, San Diego, CA, USA), soluble anti-CD28 (eBioscience). After 24 h, Jurkat cells were harvested for analysis. To assess T-cell activation, cells were stained with PE-Cy7-conjugated anti-CD69 (BioLegend, San Diego, CA, USA) antibody in FACS buffer for at least 1 h at 4 °C in the dark. Stained cells were analyzed by BD Accuri^TM^ C6 flow cytometry (BD Biosciences), and data were processed using FlowJo software (TreeStar).

Peripheral blood mononuclear cells (PBMCs) were isolated from the peripheral blood of healthy volunteers using density gradient centrifugation with Ficoll-Paque (Cat#10771, Sigma-Aldrich). All volunteers provided written informed consent prior to sample collection. For activation, PBMCs were treated with phorbol 12-myristate 13-acetate (PMA) and ionomycin. To assess T-cell exhaustion, cells were stained with BD Horizon™ BV421 Mouse Anti-Human CD8 (BD Biosciences) and FITC-conjugated anti-PD-1 (BioLegend) antibodies for 30 min at room temperature. To evaluate T-cell cytotoxicity, cells were first stained with CD8 antibody, followed by fixation with 2 % formaldehyde and permeabilization using PBS containing 0.1 % Triton X-100. Subsequently, cells were stained with APC-conjugated anti-granzyme B (GZMB) antibody in FACS buffer for 30 min at room temperature. Stained cells were analyzed by FACSLyric (BD Biosciences) and data were processed using FlowJo software (TreeStar).

**Mouse T cell isolation**

T cell isolation from mouse spleens was performed according to the manufacturer’s instructions (STEMCELL Technologies). Briefly, spleens were harvested under aseptic conditions, mechanically dissociated to generate single-cell suspensions, and subjected to red blood cell lysis using Red Blood Cell Lysing Buffer Hybri-Max (Sigma‒Aldrich). After washing, CD3⁺ T cells were purified using the CD3ε MicroBead Kit (Miltenyi biotech, Bergisch Gladbach, Germany), in which T cells were labeled with biotinylated antibodies and isolated via magnetic separation.

**Direct co-culture assay**

Isolated mouse T-cell (1 × 10^4^) was activated with soluble anti-mouse CD3ε (BioLegend), soluble anti-mouse CD28 (BioLegend). Activated T cells were then co-cultured with MC38 cells (1 × 10^4^). After 48 h, T-cell activation was assessed by flow cytometry. Cells were stained with FITC-conjugated anti-CD25 (BioLegend) or APC-conjugated anti-CD69 (BioLegend) antibody in FACS buffer for at least 1 h at 4 °C in the dark. Stained cells were analyzed by BD Accuri^TM^ C6 flow cytometry (BD Biosciences), and data were processed using FlowJo software (TreeStar). To evaluate cytokine production, IL-2 and IFN-γ levels were quantified using ELISA kits specific for IL-2 and IFN-γ (Invitrogen), according to the manufacturer’s instructions. Absorbance was measured at 450 nm using a microplate reader. MC38 cell viability was assessed by MTT assay.

**Supplementary Fig. 1. The acidic tumor microenvironment (TME) is positively correlated with dysadherin expression.**

**a** DEGs between tumor and normal tissues (GSE18105, *p <* 0.001) were analyzed using curated and GO gene sets in GSEA to identify the associated gene signatures. **b** UMAP plot based on *FXYD5* expression (GSE144735). DEGs between dysadherin-high and -low cell groups were analyzed using GSEA. **c** Trajectory analysis of different cells performed with the R package Monocle2 relies on the malignant state of the cancer cells. The expression of tumor acidosis-related genes was evaluated along the trajectory. **d** Correlation between *FXYD5* and tumor acidosis-related genes as well as gene signature of the open-source dataset (GSE106582, GSE18105, GSE144735, TCGA). Here, and throughout, Pearson’s correlation coefficient (R) was used to assess the linear relationship. **e** Network analysis via IPA revealed a potential link between dysadherin and metabolic acidosis. **f** qPCR analysis and correlation of *FXYD5* and *LAMP2* expression in CRC patient tissues. Patients were divided into three groups according to tumor stage (S1, *n* =45; S2, *n* = 62; S3, *n* = 61; total, *n* = 168). **g** Hematoxylin and eosin (H&E)-stained CRC patient tissues, matched with Fig. 1c. **h** Kaplan‒Meier survival analysis of patients with stage II and III CRC. Patients were divided into two groups according to dysadherin or LAMP2 expression (high, *n* = 61; low, *n* = 62; total, *n* = 123). Statistical significance was determined by log-rank tests. **i** Correlation between dysadherin and LAMP2 expression and Kaplan‒Meier survival analysis of the GEO dataset (GSE106584). Patients were divided into two or three groups according to dysadherin and LAMP2 expression (high, *n* =78; low, *n* =78 or high, *n* =55; low, *n* =55; others, *n* =46; total, *n* =156). The data are presented as the means ± SEMs. *, **, and *** indicate *p* < 0.05, *p* < 0.01 and *p* < 0.001, respectively. Statistical significance was determined by one-way ANOVA with Dunnett’s multiple comparison tests for comparisons among three groups. Dys, dysadherin; S1, stage 1; S2, stage 2; S3, stage 3; dCt, delta Ct, difference in the Ct value between the target gene and housekeeping gene.

**Supplementary Fig. 2. Dysadherin deficiency suppresses intestinal tumorigenesis and enhances intracellular acidification but attenuates extracellular acidification in *Apc*^Min/+^ mice.**

**a** High-resolution IF images showing magnified views of dysadherin expression in tumor tissues and adjacent normal mucosa from CRC patient samples and *Apc*^Min/+^ mice. **b** Frequency of tumor formation in the intestines of *Apc*^Min/+^; *Fxyd5*^+/+^ and *Apc*^Min/+^; *Fxyd5*^-/-^ mice. The small intestine was divided into proximal, mid, and distal thirds, and the colon was laid out separately after removing the cecum. **c** H&E-stained mouse intestinal tissues, matched with those in Fig. 1f. **d** *In situ* calibration of 8-Hydroxypyrene-1,3,6-trisulfonic acid (HPTS) fluorescence in tumor tissue. Frozen sections were exposed to pH-buffered solutions (pH 6.2–7.4) and incubated with HPTS. Confocal imaging at 450 nm excitation revealed increased fluorescence at higher pH, confirming that HPTS intensity is inversely correlated with extracellular acidity. **e** H&E-stained mouse intestinal tissues, matched with those in Fig. 1g. The graphs show the intensities of pHrodo and HPTS in the indicated groups. **f** pHrodo and HPTS analysis in mouse intestinal tumor organoids from *Apc*^Min/+;^ *Fxyd5*^+/+^ and *Apc*^Min/+^; *Fxyd*5^-/-^ mice (*n* = 5/group). **g** pHrodo and HPTS staining in size-matched mouse intestinal tumor (diameter: 2-2.5 mm) (*n* = 5/group). **h** pHrodo and HPTS analysis in size-matched mouse intestinal tumor organoids from *Apc*^Min/+^; *Fxyd5*^+/+^ and *Apc*^Min/+^; *Fxyd5*^-/-^ mice (diameter: 200 μm) (*n* = 5/group). Graphs showed the intensity of pHrodo and HPTS in the indicated groups. The data are presented as the means ± SEMs. *, **, and *** indicate *p* < 0.05, *p* < 0.01 and *p* < 0.001, respectively. Statistical significance was determined by one-way ANOVA with Dunnett’s multiple comparison tests for comparisons among three groups.

**Supplementary Fig. 3. Dysadherin regulates the malignant phenotypes of CRC cells in the acidic TME.**

**a** Proliferation was measured by a BrdU assay using CRC cells under various pH conditions (*n* = 3/group). **b** qPCR and immunoblot analysis of dysadherin in dysadherin OE HCT116 and dysadherin KO SW480 cell lines. Here, and throughout, the numbers under each image are fold changes in band intensity relative to the initial condition, determined using ImageJ. **c, d** pHrodo (c) and HPTS assay (d) of dysadherin OE HCT116 cells and dysadherin KO SW480 cells at different time points after pH 6.4 treatment (*n* = 3/group). **e** Proliferative capacity of SW480 cells treated with dysadherin-inhibitory peptide under pH 7.4 or 6.4 conditions (*n* = 3/group). **f, g** pHrodo (f) and HPTS assay (g) of SW480 cells treated with dysadherin-inhibitory peptide at various time points following pH 6.4 treatment (*n* = 3/group). **h** The pH of media supplemented with lactic acid (0, 10, or 20 mM) was measured using a Mettler Toledo InLab Routine Pro electrode. **i, j** The survival potential, proliferative capacity, and apoptotic cell population were measured under lactate-induced acidic conditions (*n* = 3/group) in dysadherin OE HCT116 cells (i) or dysadherin KO SW480 cells (j). The data are presented as the means ± SEMs. *, **, and *** indicate *p* < 0.05, *p* < 0.01 and *p* < 0.001, respectively. Statistical significance was determined by unpaired two-tailed Student’s t-tests or two-way ANOVA with the Bonferroni multiple comparison test for comparisons between two groups and one-way ANOVA with Dunnett’s multiple comparison tests for comparisons among more than three groups.

**Supplementary Fig. 4. CA9 is a downstream target of dysadherin and drives tumor progression in the acidic TME.**

**a** Kaplan‒Meier survival analysis of potential downstream genes of dysadherin in the GEO dataset (GSE143985). Patients were divided into two groups according to gene expression (high, *n* = 45; low, *n* = 46; total, *n* = 91). **b** qPCR analysis of *CA9* and the correlation between *FXYD5* and *CA9*. Patients were divided into three groups according to tumor stage (S1, *n* =45; S2, *n* = 62; S3, *n* = 61; total, *n* = 168). **c** H&E-stained CRC patient tissues, matched with those in Fig. 3c. **d** High-resolution IF image showing magnified views of dysadherin and CA9 expression in CRC patient tissues. **e** Correlation analyses of *CA9* with *FXYD5* and with a tumor acidosis-related gene signature across open-source datasets (GSE106582, GSE18105, GSE144735, TCGA). **f** Kaplan‒Meier survival analysis of patients with stage II and III CRC. Patients were divided into two groups according to CA9 expression (high, *n* = 61; low, *n* = 62; total, *n* =123). Statistical significance was determined by log-rank tests. **g** Correlation between dysadherin and CA9 expression, and Kaplan‒Meier survival analysis using GSE143985 and TCGA datasets. Patients were divided into three groups according to dysadherin and CA9 expression (high, *n* = 23 / 105; low, *n* = 24 / 106; others, *n* = 38 / 216; total, *n* = 91 / 427). **h** Correlation between dysadherin and CA9 protein levels in human CRC cell lines. **i** The efficacy of three siRNAs targeting dysadherin in SNU-254 cells was evaluated by qPCR analysis and immunoblotting. **j, k** qPCR and immunoblot analysis of dysadherin and CA9 in SNU-254 cells and patient-derived CRC cells (hCRC #1, 2) upon dysadherin knockdown (KD) with siRNA. **l** Changes in *CA9* expression in liver cancer cells (SK-HEP-1) and breast cancer cells (MDA-MB-231) upon dysadherin KD with shRNA. **m** H&E-stained mouse intestinal tissues, matched with those in Fig. 3g. The data are presented as the means ± SEMs. *, **, and *** indicate *p* < 0.05, *p* < 0.01 and *p* < 0.001, respectively. Statistical significance was determined by unpaired two-tailed Student’s t tests for comparisons between two groups and one-way ANOVA with Dunnett’s multiple comparison tests for comparisons among more than three groups.

**Supplementary Fig. 5. Dysadherin stimulates the integrin/FAK/STAT3 signaling pathway, thereby increasing the expression of CA9.**

**a** Immunoblot analyses of dysadherin, p-FAK, t-FAK, p-STAT3, t-STAT3, HIF-1α, and CA9 in dysadherin OE HCT116, dysadherin KO SW480, and dysadherin KD SNU-254 cells. **b** IF analysis of dysadherin, active integrin β1, p-FAK, and p-STAT3 in intestinal tumor tissue from 24-week-old *Apc*^Min/+;^ *Fxyd5*^+/+^ and *Apc*^Min/+^; *Fxyd*5^-/-^ mice (*n* = 5/group). The antibody clones used for detecting active integrin β1 detection was 9EG7 which recognizes specific conformational epitopes of murine active integrin β1. **c** The IC_50_s of MK-0429, PND-1186, and Stattic were evaluated in HCT116 cells by the MTT assay (*n* = 3/group). **d** Evaluation of three siRNAs targeting STAT3 in HCT116 cells by qPCR and immunoblot analyses. **e** Immunoblot analyses of dysadherin, p-FAK, t-FAK, p-STAT3, t-STAT3, and CA9 in dysadherin OE HCT116 cells and dysadherin KO SW480 cells cultured with or without fibronectin coating (10 μg/ml), and treated with or without 1 μM PND-1186, or 1 μM Stattic. **f** Immunoblot analyses of p-STAT3, t-STAT3, and CA9 in dysadherin OE HCT116 cells following STAT3 KD with siRNA. **g** CA9 promoter activity in dysadherin OE HCT116 cells after STAT3 KD. **h** IF analysis of dysadherin, active integrin β1, p-FAK, and p-STAT3 in SW480 cells with or without the dysadherin-inhibitory peptide. **i** Immunoblot analyses of dysadherin, p-FAK, t-FAK, and CA9 in SW480 cells with or without the dysadherin-inhibitory peptide. **j, k** pHrodo (j) and HPTS assay (k) of dysadherin OE HCT116 cells treated with or without 0.1 µM MK-0429, 1 µM PND-1186 or 1 µM Stattic at different time points after pH 6.4 treatment (*n* = 3/group). **l** Proliferative capacity of dysadherin OE HCT116 cells treated with or without 0.1 µM MK-0429, 1 µM PND-1186 or 1 µM Stattic. **m** qPCR analysis of *FXYD5* and *CA9* expression in dysadherin OE HCT116 cells cultured under pH 7.4 or 6.4 conditions. **n** Immunoblot analyses of dysadherin, p-FAK, t-FAK, p-STAT3, t-STAT3, and CA9 in dysadherin OE HCT116 cells cultured under pH 7.4 or 6.4 conditions. Statistical significance was determined by unpaired two-tailed Student’s t tests for comparisons between two groups and one-way ANOVA with Dunnett’s multiple comparison tests for comparisons among more than three groups.

**Supplementary Fig. 6.** **The dysadherin/CA9 axis promotes CRC cell proliferation, survival, resistance to apoptosis, and 3D tumor growth in the acidic TME.**

**a** The efficacy of three siRNAs targeting *CA9* was evaluated by qPCR analysis and immunoblotting. **b** The proliferative capacity and apoptotic cell population were assessed in CRC cell lines (HCT116, HT29, SW480, SNU-254) and patient-derived CRC cells (hCRC #1 and #2) following incubation at pH 7.4 or 6.4, with either CA9 OE or KD (*n* = 3/group). **c** The efficacy of CA9 OE in NTC and dysadherin KO SW480 cells was evaluated by qPCR and immunoblotting. **d, e** pHrodo (d) and HPTS assay (e) of NTC or dysadherin-KO SW480 cells with CA9 OE at different time points after pH 6.4 treatment (*n* = 3/group). **f** Heatmap showing the relative expression levels of proton exporters in NTC and dysadherin-KO SW480 cells with or without CA9 OE. **g** The proliferative capacity and apoptotic cell population were assessed in EV or dysadherin OE HCT116 cells with CA9 KD at pH 6.4 (*n* = 3/group). **h** 3D tumor spheroid models were generated using SW480 cells and patient-derived primary CRC cells embedded in Matrigel. Spheroid size and growth were assessed using CellTiter-Blue fluorescence-based quantification. Intra- and extracellular acidity was evaluated using pHrodo and HPTS assays, respectively (*n* = 3/group). For size-matched pH analysis, spheroids with a diameter of 100 μm were selected. The data are presented as the means ± SEMs. *, **, and *** indicate *p* < 0.05, *p* < 0.01 and *p* < 0.001, respectively. Statistical significance was determined by one-way ANOVA with Dunnett’s multiple comparison test or two-way ANOVA with the Bonferroni multiple comparison test for comparisons among more than three groups.

**Supplementary Fig. 7.** **The dysadherin/CA9 axis induces metabolic reprogramming, CAF activation, and immune evasion in the acidic TME.**

**a** Heatmap showing the relative expression of energy metabolism-related gene signatures in NTC or dysadherin-KO SW480 cells with CA9 OE under pH 7.4 or 6.4 conditions. **b** ECAR and OCR profiles were monitored in NTC or dysadherin KO SW480 cells with CA9 OE at pH 7.4 or 6.4 (*n* = 3/group). **c** ECAR and OCR profiles were monitored in CRC cell lines (HCT116, SW480) incubated at pH 7.4 or 6.4, with either CA9 OE or CA9 KD (*n* = 3/group). **d** ECAR profiles and Immunoblot analyses were performed in HCT116 cells expressing EV or dysadherin OE, treated with or without 1 mM 2-deoxy-D-glucose (2-DG), 1 μM PND-1186, or 1 μM Stattic (*n* = 3/group). ECAR were monitored using a Seahorse extracellular flux analyzer. **e** Flow cytometric analysis of intracellular ROS production in NTC or dysadherin KO SW480 cells with CA9 OE at pH 6.4 (*n* = 3/group). **f** Mitochondria ROS levels in NTC or dysadherin KO SW480 cells with or without CA9 OE at pH 6.4. **h, i** Heatmap showing the relative expression of cancer-associated fibroblast (CAF)-associated gene signatures related to angiogenesis, ECM remodeling, and immunosuppression in CAFs cultured at pH 7.4 or 6.4 (h) or CM from EV or dysadherin OE HCT116 cells with CA9 KD (i) (*n* = 3/group). **j, k** Flow cytometry of CD69^+^ Jurkat cells (j) and CD8^+^ PD-1^+^ and CD8^+^ GZMB^+^ T cells (k) incubated under pH 6.4 or CM from EV or dysadherin OE HCT116 cells with CA9 KD (*n* = 3/group). The “EV + CON media” group indicates Jurkat cells or PBMCs cultured in CM collected from HCT116 cells (empty vector + control siRNA), and the “pH 7.4” group indicates cells cultured in unconditioned RPMI media buffered at pH 7.4. These differences are therefore more likely attributable to tumor-derived soluble factors present in the CM (e.g., cytokines, metabolic byproducts, extracellular vesicles) rather than extracellular pH differences. The data are presented as the means ± SEMs. *, **, and *** indicate *p* < 0.05, *p* < 0.01 and *p* < 0.001, respectively. Statistical significance was determined by one-way ANOVA with Dunnett’s multiple comparison test or two-way ANOVA with the Bonferroni multiple comparison test for comparisons among more than three groups.

**Supplementary Fig. 8. The dysadherin/CA9 axis enhances CRC cell migration and invasion under acidic TME conditions *in vitro*.**

**a** Migration and invasion of dysadherin OE HCT116 cells and dysadherin KO SW480 cells were assessed under pH 7.4 or 6.4 conditions (*n* = 3/group). **b** Migration and invasion of NTC or dysadherin-KO SW480 cells with CA9 OE were assessed under pH 7.4 or 6.4 conditions (*n* = 3/group). The data are presented as the means ± SEMs. *, **, and *** indicate *p* < 0.05, *p* < 0.01 and *p* < 0.001, respectively. Statistical significance was determined by one-way ANOVA with Dunnett’s multiple comparison test for comparisons among more than three groups.

**Supplementary Fig. 9. Dysadherin promotes hepatic metastasis of CRC through the integrin/FAK/STAT3/CA9 axis in an intrasplenic xenograft model.**

**a** Heatmap comparing the relative expression relative expression of proton exporters in metastatic nodules in the mouse liver. **b** Heatmap comparing the relative expression of genes related to proliferation, apoptosis, and metastasis in metastatic nodules. **c** IF analysis of dysadherin, active integrin β1, p-FAK, and p-STAT3 in metastatic nodules (*n* = 5/group). The antibody clones used for detecting active integrin β1 detection was 12G10 which recognizes specific conformational epitopes of human active integrin β1. **d** IF analysis of α-SMA and collagen Ⅰ in metastatic nodules (*n* = 5/group). **e** Graphs showing the intensity of dysadherin and CA9 expression in the carcinoma *in situ* and metastatic cancer tissues of CRC patients. The data are presented as the means ± SEMs. *, **, and *** indicate *p* < 0.05, *p* < 0.01 and *p* < 0.001, respectively. Statistical significance was determined by one-way ANOVA with Dunnett’s multiple comparison test for comparisons among more than three groups.

**Supplementary Fig. 10. Dysadherin deficiency does not affect T-cell activation.**

**a** Flow cytometry analysis of CD3⁺ CD69⁺ (left) and CD3⁺ CD25⁺ (right) T-cells isolated from wild-type and *Fxyd5*⁻/⁻ mice following CD3/CD28 stimulation (*n* = 3/group). **b** IL-2 and IFN-γ levels in culture supernatants following T-cell activation, measured by ELISA (*n* = 3/group). **c** Cell viability of MC38 cells after co-culture with activated T-cells derived from WT or *Fxyd5*^⁻/⁻^ mice (*n* = 3/group). The data are presented as the means ± SEMs. *, **, and *** indicate *p* < 0.05, *p* < 0.01 and *p* < 0.001, respectively. Statistical significance was determined by one-way ANOVA with Dunnett’s multiple comparison test for comparisons among more than three groups.

**Supplementary Fig. 11** Predicted HIF-1α and NF-κB binding sites in the FXYD5 promoter region, identified using eukaryotic promoter database (EPD) web tool.

**Supplementary Fig. 12** 3D tumor spheroid models were generated using SW480 cells and patient-derived primary CRC cells with or without the dysadherin-inhibitory peptide. *FXYD5* and *CA9* mRNA expression was measured by qPCR. Spheroid size and growth were assessed using CellTiter-Blue fluorescence-based quantification. Intra- and extracellular acidity was evaluated using pHrodo and HPTS assays, respectively (*n* = 3/group).

**Table S2. Clinical information of stage II and III CRC patient samples used for IF staining and survival analysis (*n* = 123)**

**- Clinical information**

| **# of**  **Patients** | **Site** | **Sex** | **TNM stage** | | | **Stage** |
| --- | --- | --- | --- | --- | --- | --- |
|  |  |  | **Tumor**  **depth** | **Lymph node**  **metastasis** | **Distant**  **metastasis** |  |
| 17116281 | colon | M | T3 | N0 | M0 | 2 |
| 166859652011 | colon | M | T3 | N0 | M0 | 2 |
| 17600077 | colon | M | T3 | N0 | M0 | 2 |
| 70971962 | rectum | M | T3 | N0 | M0 | 2 |
| 14705137 | colon | F | T3 | N0 | M0 | 2 |
| 70982286 | rectum | F | T3 | N0 | M0 | 2 |
| 70980798 | rectum | M | T3 | N0 | M0 | 2 |
| 10964811 | colon | M | T3 | N0 | M0 | 2 |
| 04849161 | colon | M | T3 | N0 | M0 | 2 |
| 70991558 | colon | M | T3 | N0 | M0 | 2 |
| 70991367 | colon | M | T3 | N0 | M0 | 2 |
| 17838878 | colon | M | T3 | N0 | M0 | 2 |
| 70987082 | colon | M | T4 | N0 | M0 | 2 |
| 12090503 | rectum | F | T3 | N0 | M0 | 2 |
| 14338558 | colon | M | T3 | N0 | M0 | 2 |
| 71003505 | colon | M | T3 | N0 | M0 | 2 |
| 71004283 | colon | M | T3 | N0 | M0 | 2 |
| 07060266 | colon | F | T3 | N0 | M0 | 2 |
| 71007414 | rectum | M | T3 | N0 | M0 | 2 |
| 71010972 | rectum | M | T3 | N0 | M0 | 2 |
| 71014363 | colon | M | T3 | N0 | M0 | 2 |
| 18328119 | rectum | M | T3 | N0 | M0 | 2 |
| 71016822 | colon | F | T3 | N0 | M0 | 2 |
| 17582691 | colon | M | T4 | N0 | M0 | 2 |
| 71029709 | colon | M | T3 | N0 | M0 | 2 |
| 01748856 | rectum | M | T4 | N0 | M0 | 2 |
| 71023619 | colon | M | T3 | N0 | M0 | 2 |
| 70471102 | colon | M | T3 | N0 | M0 | 2 |
| 18301077 | colon | F | T3 | N0 | M0 | 2 |
| 71025944 | colon | M | T3 | N0 | M0 | 2 |
| 71036347 | colon | M | T3 | N0 | M0 | 2 |
| 71037976 | rectum | M | T3 | N0 | M0 | 2 |
| 71040975 | colon | M | T3 | N0 | M0 | 2 |
| 71034747 | rectum | M | T3 | N0 | M0 | 2 |
| 71038481 | rectum | M | T3 | N0 | M0 | 2 |
| 15120242 | colon | F | T3 | N0 | M0 | 2 |
| 70327925 | colon | M | T4 | N0 | M0 | 2 |
| 71045602 | colon | F | T3 | N0 | M0 | 2 |
| 15709306 | colon | M | T3 | N0 | M0 | 2 |
| 16108804 | colon | M | T3 | N0 | M0 | 2 |
| 71003674 | rectum | F | T3 | N0 | M0 | 2 |
| 71056834 | rectum | M | T3 | N0 | M0 | 2 |
| 09733559 | colon | F | T3 | N0 | M0 | 2 |
| 12543257 | colon | F | T3 | N0 | M0 | 2 |
| 71055055 | rectum | F | T3 | N0 | M0 | 2 |
| 17450367 | rectum | M | T3 | N0 | M0 | 2 |
| 71053758 | rectum | F | T4 | N0 | M0 | 2 |
| 71060641 | colon | M | T3 | N0 | M0 | 2 |
| 06823194 | colon | M | T3 | N0 | M0 | 2 |
| 12651231 | colon | F | T3 | N0 | M0 | 2 |
| 08181158 | rectum | M | T3 | N0 | M0 | 2 |
| 71065438 | rectum | F | T3 | N0 | M0 | 2 |
| 71068634 | rectum | M | T3 | N0 | M0 | 2 |
| 71072815 | colon | M | T3 | N0 | M0 | 2 |
| 14501044 | colon | M | T3 | N0 | M0 | 2 |
| 04922839 | colon | F | T3 | N0 | M0 | 2 |
| 12935368 | colon | F | T3 | N0 | M0 | 2 |
| 71080523 | colon | M | T4 | N0 | M0 | 2 |
| 71077142 | rectum | M | T3 | N0 | M0 | 2 |
| 13006848 | rectum | M | T3 | N0 | M0 | 2 |
| 71078747 | rectum | M | T3 | N0 | M0 | 2 |
| 71082857 | colon | M | T3 | N0 | M0 | 2 |
| 71083657 | colon | M | T4 | N1 | M0 | 3 |
| 70970453 | colon | F | T3 | N1 | M0 | 3 |
| 04687158 | colon | F | T3 | N1 | M0 | 3 |
| 11689237 | colon | F | T2 | N1 | M0 | 3 |
| 70984418 | colon | F | T3 | N1 | M0 | 3 |
| 70986187 | colon | M | T4 | N1 | M0 | 3 |
| 70982692 | rectum | F | T3 | N2 | M0 | 3 |
| 16991806 | rectum | M | T4 | N2 | M0 | 3 |
| 70987759 | colon | M | T4 | N2 | M0 | 3 |
| 13312476 | colon | M | T4 | N2 | M0 | 3 |
| 08869579 | colon | M | T3 | N2 | M0 | 3 |
| 05875523 | colon | M | T3 | N1 | M0 | 3 |
| 70991326 | colon | M | T3 | N1 | M0 | 3 |
| 18612726 | colon | F | T3 | N1 | M0 | 3 |
| 70991852 | colon | M | T3 | N1 | M0 | 3 |
| 70991877 | colon | M | T3 | N1 | M0 | 3 |
| 17178937 | colon | M | T3 | N1 | M0 | 3 |
| 70990406 | colon | F | T3 | N1 | M0 | 3 |
| 70993739 | colon | M | T3 | N1 | M0 | 3 |
| 70989574 | colon | M | T3 | N1 | M0 | 3 |
| 70997623 | rectum | M | T3 | N2 | M0 | 3 |
| 71001722 | rectum | M | T3 | N1 | M0 | 3 |
| 04331933 | rectum | M | T3 | N2 | M0 | 3 |
| 18484754 | colon | F | T3 | N2 | M0 | 3 |
| 04798386 | rectum | F | T3 | N2 | M0 | 3 |
| 12824235 | rectum | M | T3 | N1 | M0 | 3 |
| 17141297 | colon | M | T3 | N1 | M0 | 3 |
| 10191663 | colon | F | T3 | N1 | M0 | 3 |
| 11183012 | colon | M | T4 | N2 | M0 | 3 |
| 71010907 | colon | M | T3 | N1 | M0 | 3 |
| 71000516 | colon | M | T4 | N1 | M0 | 3 |
| 05719973 | colon | F | T3 | N1 | M0 | 3 |
| 70995617 | colon | F | T3 | N1 | M0 | 3 |
| 71015147 | rectum | F | T3 | N2 | M0 | 3 |
| 70999861 | rectum | F | T2 | N1 | M0 | 3 |
| 71009333 | colon | M | T3 | N1 | M0 | 3 |
| 71023515 | colon | M | T3 | N1 | M0 | 3 |
| 06798461 | rectum | M | T3 | N1 | M0 | 3 |
| 71013045 | colon | M | T3 | N1 | M0 | 3 |
| 71013348 | rectum | M | T3 | N1 | M0 | 3 |
| 71030718 | rectum | M | T3 | N2 | M0 | 3 |
| 71022573 | rectum | M | T3 | N2 | M0 | 3 |
| 71033802 | colon | M | T3 | N1 | M0 | 3 |
| 04439445 | rectum | F | T3 | N2 | M0 | 3 |
| 18286847 | rectum | F | T2 | N1 | M0 | 3 |
| 12256386 | rectum | F | T3 | N2 | M0 | 3 |
| 71047123 | rectum | M | T3 | N2 | M0 | 3 |
| 11841258 | colon | F | T4 | N2 | M0 | 3 |
| 14847893 | colon | F | T3 | N1 | M0 | 3 |
| 17806612 | rectum | F | T3 | N1 | M0 | 3 |
| 13781706 | colon | F | T4 | N1 | M0 | 3 |
| 15129346 | colon | M | T4 | N2 | M0 | 3 |
| 71047699 | rectum | M | T3 | N2 | M0 | 3 |
| 14039298 | rectum | M | T3 | N1 | M0 | 3 |
| 70370712 | colon | F | T3 | N2 | M0 | 3 |
| 71062345 | colon | M | T3 | N1 | M0 | 3 |
| 11472615 | colon | M | T3 | N1 | M0 | 3 |
| 71077652 | colon | F | T3 | N1 | M0 | 3 |
| 70416859 | rectum | M | T3 | N2 | M0 | 3 |
| 08731985 | rectum | M | T3 | N2 | M0 | 3 |
| 03641161 | colon | M | T2 | N1 | M0 | 3 |

**- Associations of clinicopathological characteristics with resected CRC**

|  | Gene Expression | | | | |
| --- | --- | --- | --- | --- | --- |
| Variable | Dys^L^, LAMP2^L^  (*n* = 45) | Dys^H^, LAMP2^L^ and Dys^L^, LAMP2^H^  (*n* = 26) | | Dys^H^, LAMP2^H^  (*n* = 52) | *p* value |
| Age (mean ± SD) | 63.82 ± 9.38 | | 64.29 ± 9.32 | 66.84 ± 9.49 | 0.504 |
| Sex |  | |  |  | 0.362 |
| Male | 28 (62.2%) | | 15 (57.7%) | 25 (48.1%) |  |
| Female | 17 (37.8%) | | 11 (42.3%) | 27 (51.9%) |  |
| T stage |  | |  |  |  |
| T2 | 0 (0.0%) | | 2 (7.7%) | 2 (3.8%) | 0.172 |
| T3 | 42 (93.3%) | | 19 (73.1%) | 42 (80.8%) |  |
| T4 | 3 (6.7%) | | 5 (19.2%) | 8 (15.4%) |  |
| N stage |  | |  |  |  |
| N0 | 39 (86.7%) | | 15 (57.7%) | 8 (15.4%) | <0.0001 |
| N1 | 4 (8.9%) | | 7 (26.9%) | 28 (53.8%) |  |
| N2 | 2 (4.4%) | | 4 (15.4%) | 16 (30.8%) |  |
| Tumor differentiation |  | |  |  | 0.143 |
| Well | 18 (40.0%) | | 12 (46.2%) | 11 (21.2%) |  |
| Moderately | 24 (53.3%) | | 13 (50.0%) | 35 (67.3%) |  |
| Poorly | 3 (6.7%) | | 1 (3.8%) | 6 (11.5%) |  |

|  | Gene Expression | | | |
| --- | --- | --- | --- | --- |
| Variable | Dys^L^, CA9^L^  (*n* = 49) | Dys^H^, CA9^L^ and Dys^L^, CA9^H^  (*n* = 24) | Dys^H^, CA9^H^  (*n* = 50) | *p* value |
| Age (mean ± SD) | 65.33 ± 9.74 | 65.43 ± 9.51 | 65.79 ± 9.48 | 0.998 |
| Sex |  |  |  | 0.767 |
| Male | 29 (59.2%) | 13 (54.2%) | 26 (52.0%) |  |
| Female | 20 (40.8%) | 11 (45.8%) | 24 (48.0%) |  |
| T stage |  |  |  |  |
| T2 | 0 (0.0%) | 1 (4.2%) | 3 (6.0%) | 0.235 |
| T3 | 45 (91.8%) | 18 (75.0%) | 40 (80.0%) |  |
| T4 | 4 (8.2%) | 5 (20.8%) | 7 (14.0%) |  |
| N stage |  |  |  |  |
| N0 | 45 (91.8%) | 13 (54.2%) | 4 (8.0%) | <0.0001 |
| N1 | 3 (6.1%) | 6 (25.0%) | 30 (60.0%) |  |
| N2 | 1 (2.1%) | 5 (20.8%) | 16 (32.0%) |  |
| Tumor differentiation |  |  |  | 0.281 |
| Well | 21 (42.9%) | 8 (33.3%) | 12 (24.0%) |  |
| Moderately | 25 (51.0%) | 15 (62.5%) | 32 (64.0%) |  |
| Poorly | 3 (6.1%) | 1 (4.2%) | 6 (12.0%) |  |

**Table S3. Clinical information of CRC patient samples used for establishing patient-derived primary CRC cells**

| **Cell** | **# of Patients** | **Site** | **Sex** | **Age** | **Cell metaplasia** | **TNM Stage** | | **Metastasis** |
| --- | --- | --- | --- | --- | --- | --- | --- | --- |
|  |  |  |  |  |  | **Tumor depth** | **Lymph node metastasis** |  |
| hCRC #1 | 07674214 | colon | F | 82 | yes | T4a | N2b | Peritoneum, Liver |
| hCRC #2 | 07647259 | colon | M | 77 | yes | T3 | N1a | Retroperitoneal, Pelvic |

**Table S5. Clinical information regarding the CRC patient samples used for IF staining (*n* = 20)**

**- Clinical information**

| **# of Patients** | **Site** | **Sex** | **Age** | **Cell metaplasia** | **Pathologic diagnosis** | **TNM Stage** | |
| --- | --- | --- | --- | --- | --- | --- | --- |
|  |  |  |  |  |  | **Tumor depth** | **Lymph node metastasis** |
| 17129828 | colon | M | 67 | yes | Carcinoma *in situ* | T1 | N0 |
| 71010056 | colon | M | 69 | yes | Carcinoma *in situ* | T1 | N0 |
| 71030393 | rectum | F | 70 | yes | Carcinoma *in situ* | T2 | N0 |
| 09538529 | rectum | M | 60 | yes | Carcinoma *in situ* | Tis | N0 |
| 11647306 | rectum | F | 77 | yes | Carcinoma *in situ* | T2 | N0 |
| 71024165 | colon | M | 63 | yes | Carcinoma *in situ* | T3 | N0 |
| 71040258 | colon | F | 68 | yes | Carcinoma *in situ* | T2 | N0 |
| 71040067 | rectum | F | 69 | yes | Carcinoma *in situ* | T1 | N0 |
| 13524348 | colon | M | 60 | yes | Carcinoma *in situ* | T2 | N0 |
| 71048234 | colon | M | 67 | yes | Carcinoma *in situ* | T2 | N0 |
| 07506122 | colon | M | 64 | yes | Metastatic CRC | T3 | N0 |
| 07427312 | rectum | M | 68 | yes | Metastatic CRC | T4a | N1a |
| 07115576 | colon | F | 55 | yes | Metastatic CRC | T4a | N2b |
| 07432965 | rectum | M | 73 | yes | Metastatic CRC | T3 | N0 |
| 07076544 | colon | M | 59 | yes | Metastatic CRC | T3 | N0 |
| 07246138 | colon | F | 62 | yes | Metastatic CRC | T3 | N2b |
| 07593199 | colon | M | 64 | yes | Metastatic CRC | T3 | N1b |
| 07126837 | colon | M | 84 | yes | Metastatic CRC | T3 | N0 |
| 07620184 | colon | M | 86 | yes | Metastatic CRC | T3 | N1a |
| 07617821 | colon | F | 73 | yes | Metastatic CRC | T3 | N2a |

**- Associations of clinicopathological characteristics with resected CRC**

| Variable | Carcinoma in situ (n = 10) | Metastatic CRC (n = 10) | *p* value |
| --- | --- | --- | --- |
| Age (mean ± SD) | 67 ± 5.08 | 59.17 ± 9.56 | 0.624 |
| Sex |  |  | 0.639 |
| Male | 6 (60.0%) | 7 (70.0%) |  |
| Female | 4 (40.0%) | 3 (30.0%) |  |
| T stage |  |  |  |
| Tis | 1 (10.0%) | 0 (0.0%) | 0.098 |
| T1 | 3 (30.0%) | 0 (0.0%) |  |
| T2-3 | 6 (60.0%) | 8 (80.0%) |  |
| T4 | 0 (0.0%) | 2 (20.0%) |  |
| N stage |  |  |  |
| N0 | 10 (100.0%) | 4 (40.0%) | 0.014 |
| N1 | 0 (0.0%) | 3 (30.0%) |  |
| N2 | 0 (0.0%) | 3 (30.0%) |  |
| Tumor differentiation |  |  | 0.639 |
| Well | 4 (40.0%) | 3 (30%) |  |
| Moderately | 6 (60.0%) | 7 (70.0%) |  |
| Poorly | 0 (0.0%) | 0 (0.0%) |  |

**Table S6. List of antibodies**

**- IF staining**

| Target | Conjugate | Catalog # (company or provider) | Application |
| --- | --- | --- | --- |
| Human Dysadherin | - | M53/provided by Dr. Ino | IF |
| Human CA9 |  | PA1-16592/ ThermoFisher | IF |
| Human LAMP2 |  | ab13524/Abcam | IF |
| Human p-FAK | - | 3283/Cell Signaling Technology | IF |
| Human p-STAT3 | - | 9145/Cell Signaling Technology | IF |
| Human α-SMA | - | 19245/Cell Signaling Technology | IF |
| Human COL1A1 | - | 66948/Cell Signaling Technology | IF |
| Mouse Dysadherin | - | SC-377163/SantaCruz | IF |
| Mouse Dysadherin |  | 12166-1-AP/Proteintech | IF |
| Mouse CA9 | - | PA1-16592/ ThermoFisher | IF |
| Donkey anti-mouse | Alexa 488 | A21202/ThermoFisher | IF |
| Donkey anti-rabbit | Alexa 555 | A31572/ThermoFisher | IF |
| Donkey anti-rat | Alexa 647 | A21247/ ThermoFisher | IF |

**- Immunoblot analysis**

| Target | Conjugate | Catalog # (company or provider) | Application |
| --- | --- | --- | --- |
| Human Dysadherin | - | M53/provided by Dr. Ino | Immunoblot |
| Human CA9 |  | PA1-16592/ ThermoFisher | Immunoblot |
| Human p-FAK | - | 3283/Cell Signaling Technology | Immunoblot |
| Human t-FAK | - | 3285/Cell Signaling Technology | Immunoblot |
| Human p-STAT3 | - | 9145/Cell Signaling Technology | Immunoblot |
| Human t-STAT3 | - | 9139/Cell Signaling Technology | Immunoblot |
| Human PCNA | - | 13-3900/Invitrogen | Immunoblot |
| Human Cyclin D1 | - | Ab226977/Abcam | Immunoblot |
| Human PARP | - | 9532/Cell Signaling Technology | Immunoblot |
| Human Cleaved-PARP | - | 5625/Cell Signaling Technology | Immunoblot |
| Human Caspase 3 | - | 9662/Cell Signaling Technology | Immunoblot |
| Human Cleaved-Caspase 3 | - | 9661/Cell Signaling Technology | Immunoblot |
| Human GAPDH | - | 5174/Cell Signaling Technology | Immunoblot |
| Goat anti-mouse | HRP | 554002/BD Pharmigen™ | Immunoblot |
| Goat anti-rabbit | HRP | 554021/BD Pharmigen™ | Immunoblot |

**- ChIP assay**

| Target | Conjugate | Catalog # (company or provider) | Application |
| --- | --- | --- | --- |
| Human p-STAT3 | - | 9139/Cell Signaling Technology | ChIP |

**-FACS**

| Target | Conjugate | Catalog # (company or provider) | Application |
| --- | --- | --- | --- |
| Human CD69 | PE-Cy7 | 310911/BioLegend | FACS |
| Human CD8 | BD Horizon™ BV421 | 562428/BD Biosciences | FACS |
| Human CD279 | FITC | 135213/BioLegend | FACS |
| Human Granzyme B | APC | 372204/BioLegend | FACS |
| Mouse CD69 | APC | 104513/BioLegend | FACS |
| Mouse CD25 | FITC | 553072/BioLegend | FACS |

**Table S7. List of siRNAs**

| Target | | Sequence | |
| --- | --- | --- | --- |
|  |  | Sense | Anti-sense |
| Dysadherin | #1 | GUGUCUUCUCACCAUCGUU | AACGAUGGUGAGAAGACAC |
|  | #2 | CAGAAACAGGAGCUGACAA | UUGUCAGCACCUGUUUCUG |
|  | #3 | ACCCUCAAGCCAUCUGGUU | AACCAGAUGGCUUGAGGGU |
| CA9 | #1 | CUGACUUCAGCCGCUACUU | AAGUAGCGGCUGAAGUCAG |
|  | #2 | GAGUGAUUGAGGCCUCCUU | AAGGAGGCCUCAAUCACUC |
|  | #3 | CCAGUCCAGCUGAAUUCCU | AGGAAUUCAGCUGGACUGG |
| STAT3 | #1 | GACCGGCGUCCAGUUCACUACUAAA | UUUAGUAGUGAACUGGACGCCGGUC |
|  | #2 | CAGAGGAUCCCGGAAAUUUAACA | UGUUAAAUUUCCGGGAUCCUCUG |
|  | #3 | UGGAAAUAAUGGUGAAGGUGCUGAA | UUCAGCACCUUCACCAUUAUUUCCA |

**Table S8. List of primers**

**- real-time PCR**

| Target | Sequence | |
| --- | --- | --- |
|  | Forward | Reverse |
| Human FXYD5 | TCCCACTGATGACACCACGA | AAACCAGATGGCTTGAGGGT |
| Human  CA9 | TAGCCCTGGTTTTTGGCCTC | CTAGGCTCCAGTCTCGGCTA |
| Human  LAMP2 | CAACCCCAATACAACTCACTCC | TGCTGATGTTCACTTCCTTCAGA |
| Human  PPIA | TGCCATCGCCAAGGAGTAG | TGCACAGACGGTCACTCAAA |
| Human KRAS | GGTGGCGTAGGCAAGAGTG | TTGACCTGCTGTGTCGAGAA |
| Human KRT18 | GGCTCTGGTTCCCGGATCTC | GTCGTTCAGGCTTTGCATGG |
| Human  MCT1 | GAGCTTTCATTTCCATCGGCT | ACCTCCACCATACATGACAGC |
| Human  MCT3 | TGCAGTTCGAGGTGCTCATGGC | GTTCTTCAACACATCCACCAGGC |
| Human MCT4 | GCTGTTTCGTCATCACTGGC | GCGGACCTGTCCCGTAGA |
| Human  NHE1 | GGGCGGTGAGCAGATCAA | TCCCCAAAAACAAGGATGTGC |
| Human  NHE2 | GTTTCACCTCTCAGGCATCATGG | CAAGGTTTCGCTGACACTGCTC |
| Human  NHE3 | GTGGTGCTTCTGGATGGAGACA | CTCCTCTTCACCTTCAGCCACT |
| Human  NBCe1 | ATGGAGCAAGCCCCATGT | CCTGTCTCAATCTGATGGTCA |
| Human  NBCn1 | AGTTACCTGGTCCTGATGAAGAA | GACGCCGACGACTCTCTT |
| Human ATP6V0D1 | GGCTACTTGGAGGGACTGGT | CCTCGTTGGCCAGGAAGTTA |
| Human ATP6V1B1 | ATCCTACGAACTCCGGTGTCA | CTCGGGGTAGATGCGGGA |
| Human  ATP6V0A1 | TTTTTCTACAGTCAGAGGCTGCT | GCTTTCGATCCATTTCTTCACATCT |
| Human  ATP6V0A2 | AGGACTATTTGAGGCAAGTGCT | GACACCAGACCTCAGCAATGA |
| Human  ATP6V0A3 | CGGCTTCATCTACAACGAGTGC | TCCAGGGTAAGCATCGTGTGCT |
| Human  ATP6V0A4 | TTGGAGAGCGTCAATGTGAGGC | CTTCTGCACCTTGATGAGCCAG |
| Human  ATP6V1A | GTCATATCACTGGCGGAGACA | AGCTCCAAGACAACATCAGAGG |
| Human MYCN | CGTTCTTGGGACGCACAGT | GGCGTTCCTCCTCCAACA |
| Human CCND1 | ACAAACAGATCATCCGCAAACA | TGTTGGGGCTCCTCAGGTTC |
| Human  CCNE2 | TGTTGGCCACCTGTATTATCTGG | ATCTGGAGAAATCACTTGTTCCTATTTC |
| Human  E2F1 | GACGGCTTGAGGGGTTGAC | TGCTACGAAGGTCCTGACAC |
| Human  MYBL2 | CCCTGGTGAGGCAGTTTGG | CTGGTCAGTGCGGTTAGGG |
| Human  MYB | TGTTCCATACCCTGTAGCGTT | TCGCTTTTCCTTCTCAGGGTC |
| Human TFDP1 | AGCAGCTCTTGCCAAAAACC | GGCGTACCAATTACCACTTGC |
| Human MKI67 | GAAAGAGYGGCAACCTGCCTTC | GCACCAAGTTTTACTACATCTGCC |
| Human  Bcl-2 | GACTGAGTACCTGAACCGGC | GGGCCAAACTGAGCAGAGTC |
| Human  Bcl-XL | GCCACTTACCTGAATGACCACC | AACCAGCGGTTGAAGCGTTCCT |
| Human BIRC5 | CCTTTCTCAAGGACCACCGC | TGAAGCAGAAGAAACACTGGG |
| Human BIRC6 | AAGTCATCGACGGCACCTC | CATTAAGGTCCTTCCTACACC |
| Human  Bax | TCAGGATGCGTCCACCAAGAAG | TGTGTCCACGGCGGCAATCATC |
| Human  BAK1 | TCATCGGGGACGACATCAAC | CAAACAGGCTGGTGGCAATC |
| Human  PMAIP1 | GCGCAAGAACGCTCAACC | TCTGCCGGAAGTTCAGTTTGT |
| Human  BIM | AAGAGTTGCGGCGTATTGGA | CACACCAGGCGGACAATGTA |
| Human VEGF | CTGGTGGACATCTTCCAGGAGTA | TAATCTGCATGGTGSTGTTGGAC |
| Human PDGF | CGGATACCTCGCCCCATGTTC | TCCAAAGAATCCTCACTCCCTAC |
| Human  TNC | GGTTCCAGATATGGGAAACCTCAC | ACCGTGAGATTGTGAGCCTCT |
| Human  HGF | GGGGAGAGTTATCGAGGTCTCA | GCCATCGGGATTGCGGC |
| Human COL1A1 | GCGACAACGGCAAGGTGTT | GCCGGTGGTTTCTTGGTCG |
| Human COL1A3 | TTTCTGGGTTGGGGCAGTCT | AAGGAGGATGTTCCCATCTTG |
| Human MMP2 | AAGGATGGCAAGTACGGCTT | AAACTTGCAGGGCTGTCCTT |
| Human CXCL2 | AAGTTTGCCTTGACCCTGAA | AGGCACATCAGGTACGATCC |
| Human CXCL5 | GAGAGCTGCGTTGCGTTTG | GGATCAAGACAAATTTCCTTCCCG |
| Human CXCL10 | GGATGGCTGTCCTAGCTCTG | ATAACCCCTTGGGAAGATGG |
| Human CXCL12 | TGCCGATTCTTCGAAAGCCA | CCTGAATCCACTTTAGCTTCGG |
| Human  CXCR2 | ATGCTGTTACGGATCCTGCC | CCCCATGTGGGCCTTAAACA |
| Human  CXCR4 | AGGAACCCTGTTTCCGTGAAG | GCAGCCTGTACTTGTCCGTC |
| Human  MMP9 | CGTTCAGGGAGATGCCCATT | TCGTAGTCAGCTGTTGTGCT |
| Human  MMP7 | TCGGAGGAGATGCTCACTTCGA | GGATCAGAGGAATGTCCCATACC |
| Human  CDH-1 | GCCTCCTGAAAAGAGAGTGGAAG | TGGCAGTGTCTCTCCAAATCCG |
| Human  ZO-1 | AGAAGATAGCCCTGCAGC | AGTCCATAGGGAGATTCC |
| Human  CDH-2 | CCTCCAGAGTTTACTGCCATGAC | GTAGGATCTCCGCCACTGATTC |
| Human  TWIST | CCTGCGCAAGATCATCCCCA | GCTGCAGCTTGCCATCTTGGA |
| Human  SNAIL1 | CTGGGTGCCCTCAAGATGCA | CCGGACATGGCCTTGTAGCA |
| Human  SNAIL2 | TACCGCTGCTCCATTCCACG | CATGGGGGTCTGAAAGCTTGG |
| Human  ZEB1 | CCTACTCAGCCTCCTCCACTC | CCAGTAGCTGATGAAACAGAGGA |
| Human  VIM | ACCCGCACCAACGAGAAGGT | ATTCTGCTGCTCCAGGAAGCG |
| Human  PKM | ATGGCTGACACATTCCTGGAGC | CCTTCAACGTCTCCACTGATCG |
| Human  PFKM | AGGCAGTCATCGCCTTGCTAGA | ATCGCCTTCTGCACATCCTGAG |
| Human  ALDOA | GACACTCTACCAGAAGGCGGAT | GGTGGTAGTCTCGCCATTTGTC |
| Human  ENO1 | AGTCAACCAGATTGGCTCCGTG | CACAACCAGGTCAGCGATGAAG |
| Human  GPI | CTGGTAGACGGCAAGGATGTGA | TCCGTGATGGTCTTGCCTGTGT |
| Human  IDH | CTATGATGGTGACGTGCAGTCG | CCTCTGCTTCTACTGTCTTGCC |

**- ChIP assay**

| Target | Sequence | |
| --- | --- | --- |
|  | Forward | Reverse |
| CA9 promoter, ChIP#1 | CCTGCAATCTGTGCTCCATACTA | AGACCCTGTCTCAAAAAAGAAAAACA |
| CA9 promoter, ChIP#2 | CTTCAAGGACATGATCAAATTTGC | TGTAATGGCATGTGCCTGTAGTC |
| CA9 promoter, ChIP#3 | CCTCGGCTCAAACCATCATC | CTAGTTCTTGCTCCCTGAAATGC |
| CA9 promoter, ChIP#4 | CCACCTCAGCCTCCCAAAAT | CCTGCCACGATATGCACATT |
| CA9 promoter, ChIP#5 | GGGCAAGGTTTTGAAGGAAGT | TCTGGCTGAGAGGGAAAGCA |
| CA9 promoter, ChIP#6 | CTCCCACATACCCATTACTTAACTCA | TGCACAGGCAGAAGGTTATCG |
| CA9 promoter, ChIP#7 | GGCTGGCTGGCAAGCA | AGAGATGGAGCCAAAGTCTCACA |

**Reference**

1	Ino, Y., Gotoh, M., Sakamoto, M., Tsukagoshi, K. & Hirohashi, S. Dysadherin, a cancer-associated cell membrane glycoprotein, down-regulates E-cadherin and promotes metastasis. *Proc Natl Acad Sci U S A* **99**, 365-370 (2002).

2	Lee, C.-J. *et al.* The dysadherin/MMP9 axis modifies the extracellular matrix to accelerate colorectal cancer progression. *Nat Commun* **15**, 10422 (2024).

3	Park, S.-Y. *et al.* Dysadherin awakens mechanical forces and promotes colorectal cancer progression. *Theranostics* **12**, 4399 (2022).

4	Lee, Y. K. *et al.* Dysadherin expression promotes the motility and survival of human breast cancer cells by AKT activation. *Cancer Sci* **103**, 1280-1289 (2012).

5	Park, J.-R. *et al.* Dysadherin can enhance tumorigenesis by conferring properties of stem-like cells to hepatocellular carcinoma cells. *J Hepatol* **54**, 122-131 (2011).
